# Identification and characterization of aquaporin genes in *Arachis duranensis* and *Arachis ipaensis* genomes, the diploid progenitors of peanut

**DOI:** 10.1186/s12864-019-5606-4

**Published:** 2019-03-18

**Authors:** S. M. Shivaraj, Rupesh Deshmukh, Humira Sonah, Richard R. Bélanger

**Affiliations:** 1 Département de phytologie–Faculté des Sciences de l’agriculture et de l’alimentation, Université Laval, 2425 rue de l’Agriculture, Québec City, QC G1V 0A6 Canada; 2 National Agri-Food Biotechnology Institute (NABI), Mohali, India

**Keywords:** Aquaporin, *Arachis hypogea*, Comparative genomics, Solute transport

## Abstract

**Background:**

Aquaporins (AQPs) facilitate transport of water and small solutes across cell membranes and play an important role in different physiological processes in plants. Despite their importance, limited data is available about AQP distribution and function in the economically important oilseed crop peanut, *Arachis hypogea* (AABB). The present study reports the identification and structural and expression analysis of the AQPs found in the diploid progenitor genomes of *A. hypogea* i.e. *Arachis duranensis* (AA) and *Arachis ipaensis* (BB).

**Results:**

Genome-wide analysis revealed the presence of 32 and 36 AQPs in *A. duranensis* and *A. ipaensis*, respectively. Phylogenetic analysis showed similar numbers of AQPs clustered in five distinct subfamilies including the plasma membrane intrinsic proteins (PIPs), the tonoplast intrinsic proteins (TIPs), the nodulin 26-like intrinsic proteins (NIPs), the small basic intrinsic proteins (SIPs), and the uncharacterized intrinsic proteins (XIPs). A notable exception was the XIP subfamily where XIP1 group was observed only in *A. ipaensis* genome. Protein structure evaluation showed a hydrophilic aromatic/arginine (ar/R) selectivity filter (SF) in PIPs whereas other subfamilies mostly contained a hydrophobic ar/R SF. Both genomes contained one NIP2 with a GSGR SF indicating a conserved ability within the genus to uptake silicon. Analysis of RNA-seq data from *A. hypogea* revealed a similar expression pattern for the different AQP paralogs of AA and BB genomes. The TIP3s showed seed-specific expression while the NIP1s’ expression was confined to roots and root nodules.

**Conclusions:**

The identification and the phylogenetic analysis of AQPs in both *Arachis* species revealed the presence of all five sub-families of AQPs. Within the NIP subfamily, the presence of a NIP2 in both genomes supports a conserved ability to absorb Si within plants of the genus. The global expression profile of AQPs in *A. hypogea* revealed a similar pattern of AQP expression regardless of the subfamilies or the genomes. The tissue-specific expression of AQPs suggests an important role in the development and function of the respective organs. The AQPs identified in the present study will serve as a resource for further characterization and possible exploitation of AQPs to understand their physiological role in *A. hypogea*.

**Electronic supplementary material:**

The online version of this article (10.1186/s12864-019-5606-4) contains supplementary material, which is available to authorized users.

## Background

Aquaporins (AQPs) are small (21–34 kD) integral membrane proteins, which form channels facilitating movement of water and other small solutes across the cell membrane. Aquaporins are conspicuously present across all kingdoms of life including plants where they co-ordinate water transport from the soil to different plant parts [1–4]. Based on sequence similarity and subcelluar localization, five subfamilies of AQPs have been identified in seed plants: the plasma membrane intrinsic proteins (PIPs), the tonoplast intrinsic proteins (TIPs), the nodulin26-like intrinsic proteins (NIPs), the small basic intrinsic proteins (SIPs) and the uncategorized intrinsic proteins (XIPs) [5–8]. Variation in the number of AQP subfamilies specific to different plant species has been reported. Among the five subfamilies, XIPs are absent entirely from monocots and dicots like Brassicaceae [9–11]. In primitive land plants, two additional unique classes of AQPs, GlpF-like intrinsic protein (GIPs) and hybrid intrinsic proteins (HIPs) have been described and are presumed to have been lost in the course of evolution [12]. Among AQPs, TIPs and PIPs are specifically located in vacuolar and plasma membranes, respectively. Being the most abundant in plants, TIPs and PIPs play a central role in mediating water transport across the plant system. The SIPs were the first to be unraveled via genome sequence analysis and are generally localized in the endoplasmic reticulum (ER). NIPs are homologous to GmNod26, an abundantly expressed transcript in the peribacteroid membrane of nitrogen-fixing nodules of soybean roots [13] and are mostly found in the plasma membrane.

The general AQP structure resembles an hourglass formed by six transmembrane (TM) α helices (H1 to H6) joined by five inter-helical loops (A to E). At the center of the pore formed by the six TM domains, two different constricts are formed: one that harbors conserved NPA (Asn-Pro-Ala) motifs, and another one known as aromatic/arginine (ar/R) selectivity filter (SF) formed with four amino acids in the channel. Among the four amino acids, one is located in each of helix 2 (H2) and helix 5 (H5), and two residues are located in loop E (LE1 and LE2). These two constrictions predominantly determine solute specificity and permeability within a given AQP [9, 14, 15].

The availability of whole genome sequences in cultivated crop plants has accelerated the genome-wide identification and analysis of the AQP-encoding genes [16]. The genome-wide characterization of AQPs has revealed important properties such as their distribution, evolution and conserved structural features involved in solute transport [16]. In this context, identification and characterization of AQP genes is the first step to decipher their presence and role in regulating transport of water and other physiologically important molecules. Translating this information to crop plants carries important implications with regards to breeding or engineering plants with improved water and nutrient uptake.

Plant AQPs exhibit abundant diversity in comparison with AQPs from bacteria and animals. This assists plants to overcome their disadvantage of immobility as they encounter varied environmental and climatic conditions. While initially AQPs were largely known as water channel proteins, they are now recognized to transport a plethora of small solutes like urea, H_2_O_2_, silicon, boron, ammonia and CO_2_ [17]. The regulation of AQP genes in response to biotic and abiotic stresses has been reported in several crop plants [18–20]. Aquaporins also serve as key regulators modulating plant growth and development during various physiological and environmental states.

*Arachis hypogea* (L.), popularly known as peanut, is by far the most economically important species of the Arachis genus. It is an allotetraploid (2n = 4x = 40), thought to be derived from a single recent hybridization event between two wild ancestors, *Arachis duranensis* (AA) and *Arachis ipaensis* (BB) [21]. The crop is valued for the kernel, an important source of protein (28%), edible oil (42%), and numerous nutrients and minerals [22]. The production of the *A. hypogea* can be altered by different biotic and abiotic stresses causing significant yield losses annually. In recent years, weather fluctuations have caused severe water-deficit conditions threatening the sustainable production of *A. hypogea*. Drought causes tissue dehydration due to an imbalance between plant water uptake and transpiration [23]. These imbalances can be alleviated by AQPs, which play an important role in maintaining water balance and homeostasis under different environmental and stress conditions [24]. However, very little is known about the AQP distribution and function in *A. hypogea* (AABB), and how they could help efforts to develop more drought tolerant cultivars .

Recently the two progenitor genomes, *A. duranensis* and *A. ipensis* were sequenced to facilitate the study of the complete genome of cultivated *A. hypogea* [21]. In the present study, we took advantage of these available sequences to identify all AQPs in the diploid progenitor genomes of *A. hypogea.* Subsequently, we were able to characterize them according to their phylogenetic distribution, gene structure, conserved motifs and ar/R SF. Finally, we analyzed AQP expression in different tissues using available transcriptomic data from *A. hypogea*. This study brings novel and relevant information with regards to the many and specific functions AQPs play in *A. hypogea* and offers avenues to exploit this information to improve stress resistance in *A. hypogea*.

## Results

### Genome-wide identification and distribution of AQPs in *A. duranensis* and *A. ipaensis*

The homology based search performed in the *A. duranensis* and *A. ipaensis* genomes revealed the presence of 32 and 36 AQPs, respectively. Subsequent identification of conserved domains also confirmed all the predicted AQPs (Additional file 1). Interestingly, based on the recent release of *A. hypogea* genome, we observed 73 AQPs distributed among different subfamilies (Additional file 2). HiddenMarkov model-based prediction of transmembrane helices showed the presence of six signature transmembrane domains in 23 out of 32 AQPs in *A. duranensis* and 23 out of 36 in *A. ipaensis* (Additional file 3). Tertiary protein structure analysis of the AQPs confirmed the typical hourglass-like structure formed with six TM domains for all proteins analysed except AipNIP1–3 (Additional file 4). The *A. duranensis* and *A. ipaensis* AQPs were found to be distributed among nine out of 10 chromosomes. In *A. duranensis*, the highest number (six) of AQPs were found on chromosome 3, 9 and 10 **(**Table 1**)**. Similarly, in *A. ipaensis* the highest number (10) of AQPs was found on chromosome 3, while five AQPs each were found on chromosome 9 and 10 (Table 2).

**Table 1:** Description and distribution of aquaporins identified in *Arachis duranensis* genome

Chromosome									
Gene name	Gene ID	Gene length (bp)	Location	Start	End	Transcript length (bp)	CDS length (bp)	Protein length (aa)	Protein pI
AduNIP1–1	Aradu.JG488	3741	Chr.A04	43,643,515	43,647,255	862	816	271	7.50
AduNIP1–2	Aradu.VG4AF	4357	Chr.A03	134,601,617	134,605,973	1262	801	266	8.76
AduNIP1–3	Aradu.VW5KP	2204	Chr.A07	13,457,918	13,460,121	923	792	263	7.46
AduNIP1–4	Aradu.0NC5M	1757	Chr.A10	985,841	987,597	1255	792	263	8.98
AduNIP1–5	Aradu.HH10J	2106	Chr.A03	134,613,149	134,615,254	1596	837	278	9.82
AduNIP2–1	Aradu.0060C	3493	Chr.A04	117,725,696	117,729,188	1192	858	285	8.40
AduNIP3–1	Aradu.R2ERN	4104	Chr.A09	999,824	1,003,927	1153	933	310	7.46
AduNIP3–2	Aradu.KMP0N	10,326	Chr.A09	117,783,408	117,793,733	1579	924	307	7.87
AduPIP1–1	Aradu.16TP0	3065	Chr.A09	25,165,262	25,168,326	1102	867	288	8.91
AduPIP1–2	Aradu.A6YMT	3065	Chr.A02	80,296,959	80,300,023	1102	867	288	8.91
AduPIP1–3	Aradu.V8K6B	1315	Chr.A03	100,926,939	100,928,253	1147	648	215	9.49
AduPIP1–4	Aradu.7N61Y	1738	Chr.A07	11,079,819	11,081,556	935	897	298	9.75
AduPIP1–5	Aradu.8K8TN	3196	Chr.A10	32,763,521	32,766,716	1205	870	289	8.59
AduPIP2–1	Aradu.IIE2D	2801	Chr.A09	119,121,907	119,124,707	1035	864	287	9.04
AduPIP2–2	Aradu.7B5LR	3553	Chr.A05	108,814,107	108,817,659	1588	864	287	8.30
AduPIP2–3	Aradu.64FTH	1887	Chr.A03	1,498,572	1,500,458	840	840	279	8.30
AduPIP2–4	Aradu.6AI81	2224	Chr.A08	26,303,106	26,305,329	1650	870	289	8.30
AduSIP1–1	Aradu.NQ7IA	2526	Chr.A10	106,779,069	106,781,594	825	738	245	9.56
AduSIP1–2	Aradu.034KP	4259	Chr.A10	73,139,619	73,143,877	1538	750	249	9.83
AduSIP2–1	Aradu.FP96F	2038	Chr.A06	82,801,941	82,803,978	1296	819	272	9.97
AduTIP1–1	Aradu.KV3KX	1603	Chr.A06	53,146,526	53,148,128	1495	753	250	6.22
AduTIP1–2	Aradu.PDC3W	1076	Chr.A08	25,324,516	25,325,591	906	906	301	10.34
AduTIP1–3	Aradu.FDB38	650	Chr.A03	2,274,475	2,275,124	564	564	187	5.42
AduTIP1–4	Aradu.617EV	1395	Chr.A09	115,890,098	115,891,492	759	759	252	5.48
AduTIP2–1	Aradu.7MF1E	1424	Chr.A07	71,800,902	71,802,325	747	747	248	6.50
AduTIP2–2	Aradu.901R7	1925	Chr.A05	14,086,746	14,088,670	1248	747	248	4.64
AduTIP2–3	Aradu.LI70Z	1978	Chr.A09	3,013,042	3,015,019	774	711	236	5.95
AduTIP3–1	Aradu.DWL7L	2201	Chr.A05	84,098,086	84,100,286	1077	768	255	6.86
AduTIP4–1	Aradu.R0KJF	2604	Chr.A10	92,770,322	92,772,925	1044	744	247	6.29
AduTIP4–2	Aradu.R6IE0	1791	Chr.A10	92,690,046	92,691,836	777	777	258	6.41
AduTIP5–1	Aradu.YIH2Y	1173	Chr.A03	9,079,394	9,080,566	945	816	271	8.61
AduXIP2–1	Aradu.ERR96	3552	Chr.A06	27,359,954	27,363,505	1358	939	312	8.21

**Table 2:** Description and distribution of aquaporins identified in *Arachis ipaensis* genome

Chromosome									
Gene name	Gene ID	Gene length (bp)	Location	Start	End	Transcript length (bp)	CDS length (bp)	Protein length (aa)	Protein pI
AipNIP1–1	Araip.PA6GK	3727	Chr.B04	42,976,837	42,980,563	732	732	243	8.44
AipNIP1–2	Araip.RLY0Z	2241	Chr.B07	13,529,517	13,531,757	936	792	263	7.46
AipNIP1–3	Araip.M00I0	3437	Chr.B03	135,654,447	135,657,883	1154	783	260	8.77
AipNIP1–4	Araip.986AT	2112	Chr.B03	135,664,227	135,666,338	1425	825	274	9.81
AipNIP1–5	Araip.LMJ0Y	1402	Chr.B10	2,922,199	2,923,600	1117	729	242	8.45
AipNIP2–1	Araip.U0Y4C	5474	Chr.B04	127,659,314	127,664,787	1245	1014	337	9.43
AipNIP3–1	Araip.Y41GL	3969	Chr.B09	1,149,629	1,153,597	1173	933	310	8.22
AipNIP3–2	Araip.YMW6F	889	Chr.B03	10,093,940	10,094,828	729	729	242	10.45
AipNIP3–3	Araip.FXH2B	1536	Chr.B09	138,336,794	138,338,329	988	693	230	8.82
AipNIP4–1	Araip.21TCR	8695	Chr.B02	13,973,560	13,982,254	578	531	176	5.48
AipPIP1–1	Araip.2JP01	2055	Chr.B10	41,620,542	41,622,596	949	870	289	9.10
AipPIP1–2	Araip.5F544	1309	Chr.B05	15,347,969	15,349,277	1177	822	273	9.57
AipPIP1–3	Araip.8A339	1764	Chr.B03	103,283,242	103,285,005	1151	885	294	8.35
AipPIP1–4	Araip.64JV0	1499	Chr.B07	10,675,062	10,676,560	974	936	311	7.99
AipPIP1–5	Araip.435BM	3113	Chr.B02	92,122,120	92,125,232	1325	867	288	9.12
AipPIP2–1	Araip.V6V8W	4120	Chr.B05	149,319,342	149,323,461	1768	864	287	8.57
AipPIP2–2	Araip.AC9 T7	2847	Chr.B09	135,722,105	135,724,951	1069	864	287	8.82
AipPIP2–3	Araip.Y94H3	2432	Chr.B08	3,659,605	3,662,036	1884	870	289	8.30
AipPIP2–4	Araip.0PG5I	2298	Chr.B03	3,415,624	3,417,921	1062	942	313	8.20
AipSIP1–1	Araip.H4L5F	2636	Chr.B10	133,606,851	133,609,486	749	690	229	9.75
AipSIP1–2	Araip.GMD2H	3452	Chr.B06	113,603,174	113,606,625	1538	750	249	9.50
AipSIP2–1	Araip.191UR	2033	Chr.B06	101,940,885	101,942,917	1300	819	272	9.97
AipTIP1–1	Araip.F6TGS	1261	Chr.B08	2,832,835	2,834,095	821	756	251	7.66
AipTIP1–2	Araip.46726	1119	Chr.B03	4,308,838	4,309,956	720	594	197	6.33
AipTIP1–3	Araip.Y6XIR	1383	Chr.B09	141,147,203	141,148,585	759	759	252	6.07
AipTIP2–1	Araip.FP1A1	1992	Chr.B09	4,021,086	4,023,077	840	747	248	4.98
AipTIP2–2	Araip.U5 J78	1427	Chr.B08	596,570	597,996	747	747	248	6.50
AipTIP2–3	Araip.XJU6V	2020	Chr.B05	14,950,492	14,952,511	1333	747	248	4.64
AipTIP3–1	Araip.LJX8Z	1718	Chr.B05	144,096,215	144,097,932	849	849	282	7.14
AipTIP4–1	Araip.6ZS67	3509	Chr.B10	116,425,219	116,428,727	1030	744	247	6.50
AipTIP4–2	Araip.Y47XB	1406	Chr.B10	116,393,639	116,395,044	865	669	222	6.80
AipTIP5–1	Araip.3S8EX	1227	Chr.B03	12,294,179	12,295,405	951	816	271	8.61
AipXIP1–1	Araip.K65JZ	750	Chr.B03	10,309,339	10,310,088	740	681	226	8.26
AipXIP1–2	Araip.Y3AUB	2243	Chr.B03	10,323,465	10,325,707	992	762	253	8.87
AipXIP1–3	Araip.BE0YC	1942	Chr.B03	10,239,454	10,241,395	867	867	288	4.73
AipXIP2–1	Araip.UB1TB	4362	Chr.B06	35,671,253	35,675,614	1053	1053	350	7.66

### Phylogenetic and gene structure analysis of AQPs in *A. duranensis* and *A. ipaensis*

**Fig. 1:**
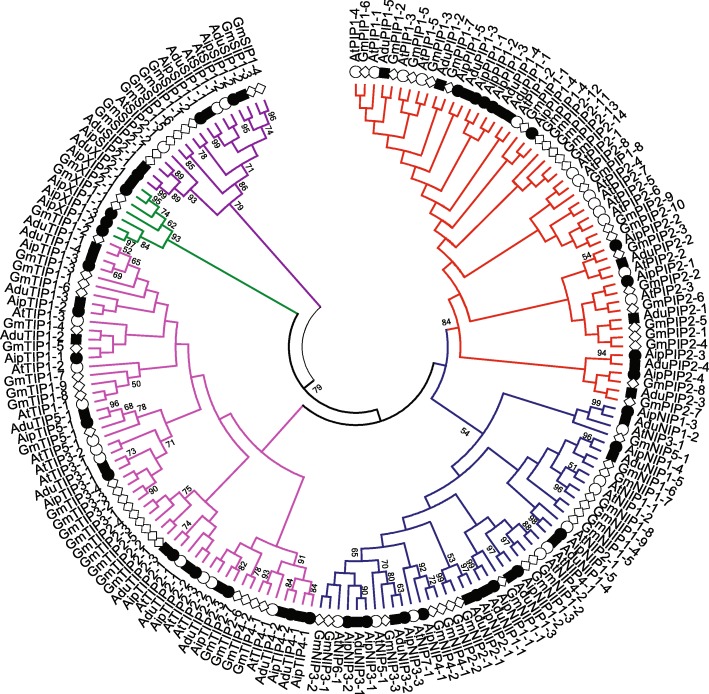
Phylogenetic tree of *Arachis duranensis* and *Arachis ipaensis* aquaporins along with *Arabidopsis thaliana* and *Glycine max***.** The analysis grouped aquaporins into five different clusters. The genes from *A. duranensis*, *A. ipaensis*, *A. thaliana* and *G.max* are preceded by the prefixes Adu, Aip, At, and Gm, respectively. The number next the branches represents bootstrap values ≥50% based on 1000 resamplings

Phylogenetic analysis of AQP candidates from *A. duranensis* and *A. ipaensis* along with known AQPs from *Arabidopsis thaliana* and *Glycine max* formed five distinct clusters representing different classes of AQPs (Fig. 1). The AQP candidates were classified according to their respective cluster. In *A. duranensis,* AQPs were grouped into nine PIPs, 11 TIPs, eight NIPs, three SIPs and one XIP. For their part, AQP candidates from *A. ipaensis* were grouped into nine PIPs, 10 TIPs, 10 NIPs, three SIPs and four XIPs. The AduPIPs and AipPIPs formed two major sub-groups of PIP1s and PIP2s comprising five and four members, respectively. Likewise, the TIP family classified into five subclusters containing a different number of TIPs in each subcluster. NIPs formed three (NIP1, NIP2 and NIP3) and four (NIP1, NIP2 NIP3 and NIP4) groups in *A. duranensis* and *A. ipaensis* respectively. The SIPs from both species formed two groups, SIP1 and SIP2, containing two and one members, respectively. Among XIPs, no XIP1 was found and only a single member of XIP2 (AduXIP2–1) was observed in *A. duranensis*. In *A. ipaensis,* XIP1s had three members (AipXIP1–1, AipXIP1–2and AipXIP1–3) and XIP2s (AipXIP2–1) had one member.

**Fig. 2:**
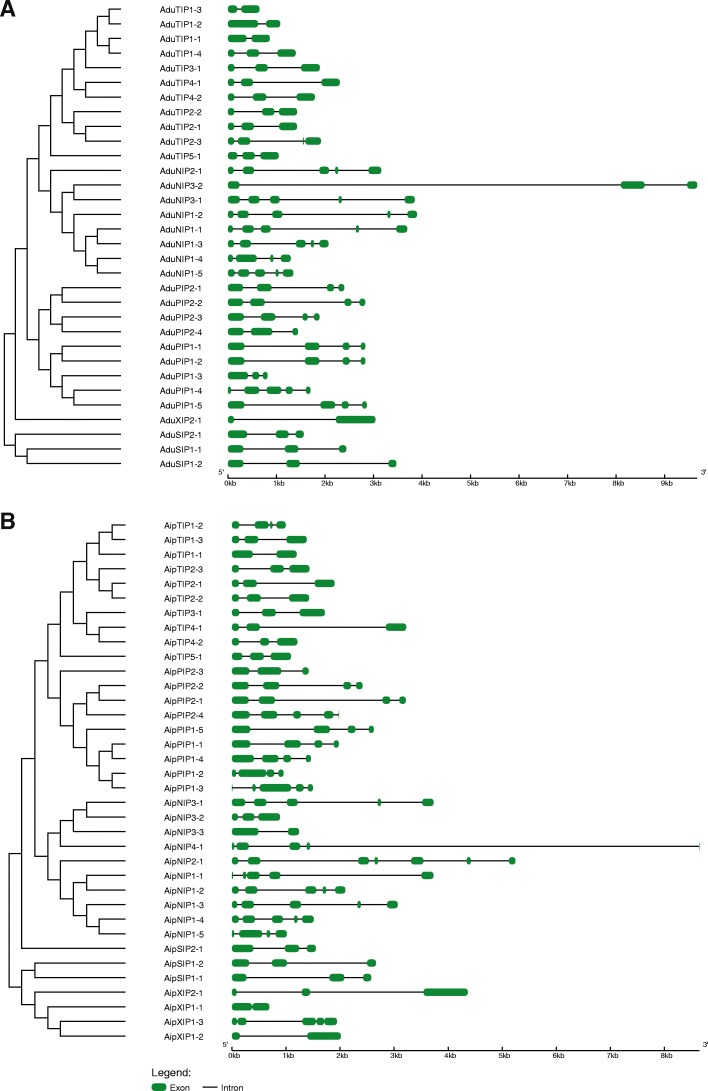
Exon–intron organization of aquaporin genes identified in genomes of (**a**) *Arachis duranensis* and (**b**) *Arachis ipaensis*. Graphical output of the gene model was obtained using Gene Structure Display Server (http://gsds.cbi.pku.edu.cn/). Exons are shown as geen boxes and introns are shown as black lines. The scale shown at the bottom reperesents gene length in base pairs

Aquaporins from both species displayed less variation in CDS length (*A. durensis*: 564 bp to 939 bp; *A. ipaensis*: 531 bp to 1053 bp) than in gene length (*A. durensis*: 650 bp to 10,326 bp; *A. ipaensis*: 750 bp to 8695 bp). Gene structure analysis revealed considerable variation in both number and length of introns and exons that resulted in gene length variation (Fig. 2). In *A. duranensis*, the number of introns varied from one (AduTIP1–1, AduTIP1–2, AduTIP1–3, AduTIP2–1) to 4 (AduNIP1–1, AduNIP1–2, AduNIP1–3, AduNIP1–5, AduNIP2–1, AduNIP3–1), while in *A. ipaensis*, this number varied from one (AipTIP1–1, AipXIP1–1, AipXIP1–2, AipNIP3–3) to six (AipNIP2–1).

### Characterization of NPA motif, transmembrane domains and sub-cellular localization of *A. duranensis* and *A. ipaensis* AQPs

Candidate *Arachis* AQPs were found to have differences in NPA motifs and residues at ar/R SF **(**Table 3 and Table 4**)**. In both species, all PIPs and TIPs displayed two conserved NPA motifs. Among NIPs, NIP1s and NIP2s showed two conserved NPA domains, while NIP3s showed variation. Among NIP3s the variation included a substitution of alanine by serine or valine and substitution of asparagine by lysine. Additionally, all the members of the XIP and SIP subfamilies had varying NPA domains. All PIP subfamily members from both studied species displayed conservation at the ar/R SF residues (Table 3 and Table 4) with phenylalanine at H2, histidine at H5, threonine at LE1 and arginine at LE2 (Table 3 and Table 4). Most of the TIP subfamily members showed group specific conservation of ar/R SF. For instance, all TIP1s contained Histidine-Isoleucine-Alanine-Valine, while all TIP2s comprised of Histidine-Isoleucine-Glycine-Arginine in both the species. Similarly, NIPs also displayed subgroup specific conservation of ar/R SF except NIP3s, which displayed variation in the SF.

**Table 3:** Conserved domains, selectivity filter and amino acid residues of aquaporins in *Arachis duranensis* genome

Loci	NPA (LB)	NPA (LE)	ar/R selectivity filters			
			H2	H5	LE1	LE2
Plasma membrane intrinsic proteins (PIPs)						
AduPIP1–1	NPA	NPA	F	H	T	R
AduPIP1–2	NPA	NPA	F	H	T	R
AduPIP1–3	NPA	NPA	F	H	T	R
AduPIP1–4	NPA	NPA	F	H	T	R
AduPIP1–5	NPA	NPA	F	H	T	R
AduPIP2–1	NPA	NPA	F	H	T	R
AduPIP2–2	NPA	NPA	F	H	T	R
AduPIP2–3	NPA	NPA	F	H	T	R
AduPIP2–4	NPA	NPA	F	H	T	R
Tonoplast intrinsic proteins (TIPs)						
AduTIP1–1	NPA	NPA	H	I	A	V
AduTIP1–2	NPA	NPA	H	I	A	V
AduTIP1–3	NPA	NPA	–	I	A	V
AduTIP1–4	NPA	NPA	H	I	A	V
AduTIP2–1	NPA	NPA	H	I	G	R
AduTIP2–2	NPA	NPA	H	I	G	R
AduTIP2–3	NPA	NPA	H	I	G	R
AduTIP3–1	NPA	NPA	H	I	A	L
AduTIP4–1	NPA	NPA	S	I	V	R
AduTIP4–2	NPA	NPA	H	I	A	R
AduTIP5–1	NPA	NPA	N	V	G	C
Nodulin-26 like intrisic proteins (NIPs)						
AduNIP1–1	NPA	NPA	W	V	A	R
AduNIP1–2	NPA	NPA	W	V	A	R
AduNIP1–3	NPA	NPA	W	V	A	R
AduNIP1–4	NPA	NPA	W	V	A	R
AduNIP1–5	NPA	NPA	W	V	A	R
AduNIP2–1	NPA	NPA	G	S	G	R
AduNIP3–1	NPA	NPV	T	I	G	R
AduNIP3–2	NPS	NPV	A	I	G	R
Small basic intrinsic proteins (SIPs)						
AduSIP1–1	NPT	NPA	F	V	P	I
AduSIP1–2	NPT	NPA	V	V	P	N
AduSIP2–1	NPL	NPA	S	H	G	S
Uncharacterized intrinsic proteins (XIPs)						
AduXIP2–1	SPV	NPA	V	V	V	R

**Table 4:** Conserved domains, selectivity filter and amino acid residues of aquaporins in *Arachis ipaensis* genome

Loci	NPA (LB)	NPA (LE)	ar/R selectivity filters			
			H2	H5	LE1	LE2
Plasma membrane intrinsic proteins (PIPs)						
AipPIP1–1	NPA	NPA	F	H	T	R
AipPIP1–2	NPA	NPA	F	H	T	R
AipPIP1–3	NPA	NPA	F	H	T	R
AipPIP1–4	NPA	NPA	F	H	T	R
AipPIP1–5	NPA	NPA	F	H	T	R
AipPIP2–1	NPA	NPA	F	H	T	R
AipPIP2–2	NPA	NPA	F	H	T	R
AipPIP2–3	NPA	NPA	F	H	T	R
AipPIP2–4	NPA	NPA	F	H	T	R
Tonoplast intrinsic proteins (TIPs)						
AipTIP1–1	NPA	NPA	H	I	A	V
AipTIP1–2	NPA	DTA	H	–	A	V
AipTIP1–3	NPA	NPA	H	I	A	V
AipTIP2–1	NPA	NPA	H	I	G	R
AipTIP2–2	NPA	NPA	H	I	G	R
AipTIP2–3	NPA	NPA	H	I	G	R
AipTIP3–1	NPA	NPA	H	I	A	L
AipTIP4–1	NPA	NPA	S	I	A	R
AipTIP4–2	NPA	NPA	H	I	A	R
AipTIP5–1	NPA	NPA	N	V	G	C
Nodulin-26 like intrisic proteins (NIPs)						
AipNIP1–1	NPA	NPA	W	–	A	R
AipNIP1–2	NPA	NPA	W	V	A	R
AipNIP1–3	NPA	NPA	W	V	A	R
AipNIP1–4	NPA	NPA	W	V	A	R
AipNIP1–5	NPA	NPA	W	V	A	R
AipNIP2–1	NPA	NPA	G	S	G	R
AipNIP3–1	NPA	NPV	T	I	G	R
AipNIP3–2	NPA	KPV	C	Y	R	M
AipNIP3–3	NPS	NPV	A	I	G	R
AipNIP4–1	NPA	–	A	V	–	–
Small basic intrinsic proteins (SIPs)						
AipSIP1–1	NPT	NPA	F	L	P	I
AipSIP1–2	NPT	NPA	V	V	P	N
AipSIP2–1	NPL	NPA	S	H	G	S
Uncharacterized intrinsic proteins (XIPs)						
AipXIP1–1	SPT	NPA	–	I	V	R
AipXIP1–2	SST	NPT	I	V	V	S
AipXIP1–3	SST	NPT	I	V	V	R
AipXIP2–1	SPV	NPA	V	V	V	R

To characterize spatial expression of AQPs from both species, their subcellular localizations were predicted in silico (Additional file 5). In *A. duranensis* and *A. ipeanensis*, the majority of the PIP homologs were predicted to be localized in the plasma membrane but AduPIP1–4 that was predicted to be localized in the mitochondria. Expectedly, most TIP sub-family members were predicted to be in the vacuole. However, a few family members were predicted to be localized in the cytoplasm (AduTIP1–1, AipTIP3–1), the chloroplast (AduTIP1–2, AduTIP5–1, AipTIP1–3, AipTIP5–1), Mitochondria (AduTIP3–1) and the plasma membrane (AipTIP2–2). Most NIPs from both species were expected to be found in the plasma membrane. The SIP family members had candidates in different sites including the plasma membrane (AduSIP1–1, AipSIP1–1, AipSIP1–2), the chloroplast (AduSIP1–2) and the cytoplasm (AduSIP2–1, AipSIP2–1). The members of XIPs were found to be likely localized in the cytoplasm, chloroplast, or nucleus.

### Aquaporin expression profiling across different tissues in *A. hypogea*

**Fig. 3:**
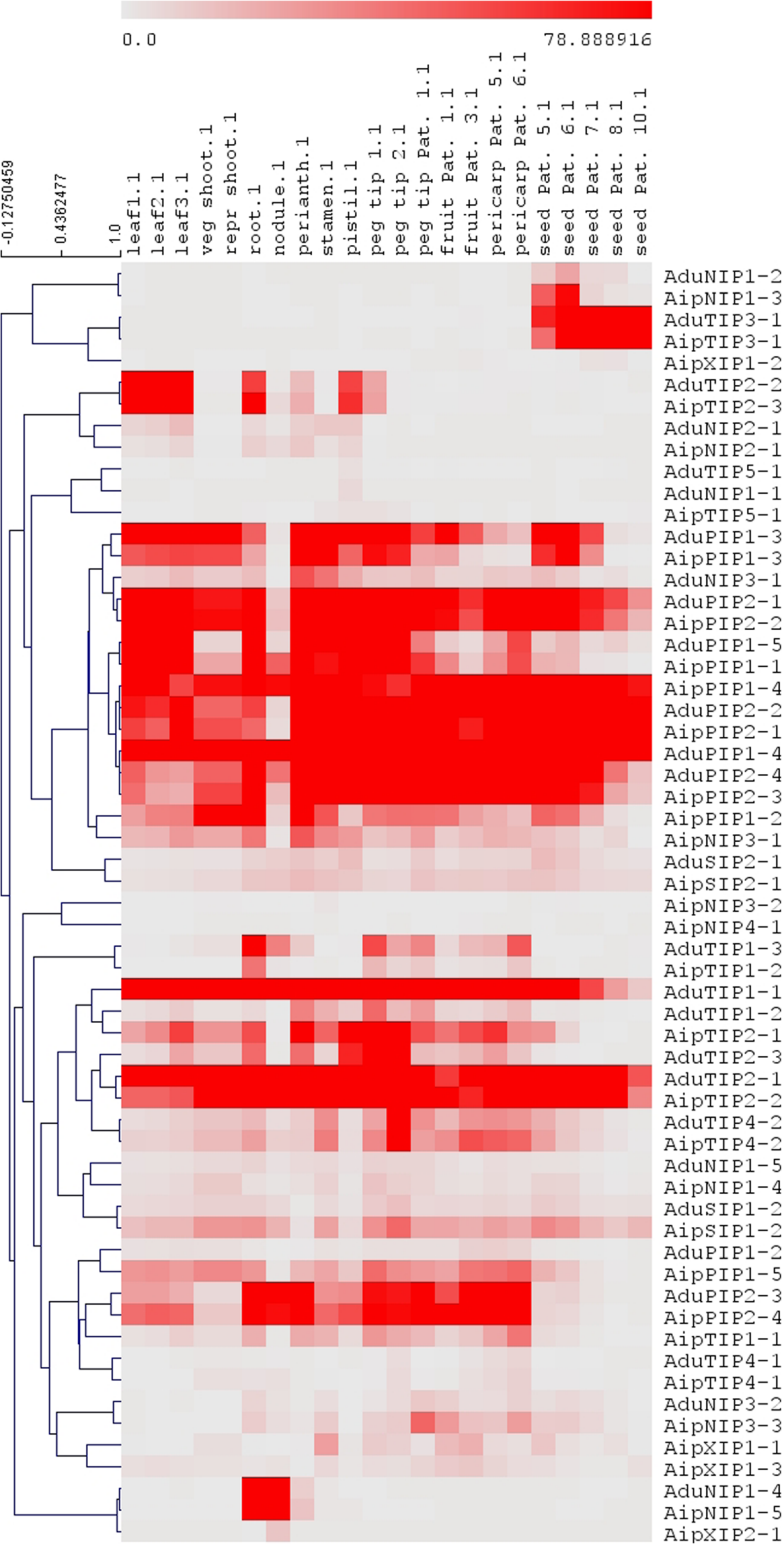
Analysis of the expression of *Arachis hypogea* aquaporins in different tissues using RNA-seq data (PRJNA291488, BioProject). Normalized expression of aquaporins in terms of reads per kilobase of transcript per million mapped reads (RPKM) showing higher levels of PIP and TIP expression compared to NIP and XIP expression across the different tissues analyzed

Analysis of RNA-seq data from *A. hypogea* showed expression of 27 out of 32, and 32 out of 36 identified AQPs from *A. duranensis* and *A. ipeanensis*, respectively. Similar patterns of expression for members of different AQP subfamilies from both AA and BB genomes were observed. Analysis of expression of AQPs at different developmental stages revealed a higher expression of PIPs followed by TIPs, SIPs, NIPs and XIPs (Fig. 3). In general, PIPs showed higher expressions across all tissues analyzed. Among TIPs, TIP2s (AduTIP2–2 and AipTIP2–3) showed higher expression in the roots, pistil and leaves. Higher expression of TIP3s (AduTIP3–1 and AipTIP3–1) was observed in all the five different developmental stages of seeds. Similarly, few NIPs (AduNIP1–2, AipNIP1–3) showed high to moderate expression in four out of five different developmental stages of seeds. The AduNIP1–4 and AipNIP1–5 showed strong expression in root nodules. Among XIPs, no expression of the unique XIP member specific to the AA genome (AduXIP2–1) was observed. However, the BB genome specific XIP members, AipXIP2–1 accumulated at higher levels in nodules of *A. hypogea.*

## Discussion

In this study, we exploited the availability of the whole genome sequence of *A. duranensis* and *A. ipeansis*, the progenitors of cultivated *A. hypogea*, [21] to provide an exhaustive identification and characterization of *A. hypogea* AQPs as a way to facilitate a better understanding of their roles in the development of the plant. Although the full genome of *A. hypogea* has recently been made available, genome-wide study of AQPs in true diploid progenitor species provides some advantages over its analysis in a highly complex and polyploid genome like peanut. Indeed, it is more informative about the relative importance and function of each aquaporin in its respective diploid progenitor, and about the impact of genome polyploidization on AQP gene structure, function and dosage-dependence on gene expression pattern. The advent of next generation sequencing platforms has enabled the decoding of AQPs in many plant species [25–27] and has highlighted their many functions in metabolism regulation, namely in the case of biotic and abiotic stresses [28], information that can have many positive implications in developing new varieties better adapted to stress conditions.

The number of AQPs identified in *A. duranensis* and *A. ipaensis*, 32 and 36, was found to be fairly proportional to their respective genome size of 1.25 and 1.56 Gb [21]. By comparison, many dicots such as Arabidopsis (35) [7], *Phaseolus* (41) [29], and pigeon pea (40) [30] bear similar numbers. On the other hand, some plant species such as canola which evolved with polyploidization contains as many as 120 AQPs [28]. However, notwithstanding this lower number of AQP candidates, the phylograms of both species showed homologs representing all five subfamilies (PIPs, TIPs, NIPs, SIPs and XIPs) as observed in most higher plant species. The presence of XIPs is particularly interesting since all monocots and dicots belonging to the *Brassicaceae* family are characterized by a complete absence [6, 7, 27]. The analysis of *A. hypogea* genome revealed the presence of 73 AQPs representing homologs of most of the AQPs identified from its progenitor genomes, *A. duranensis* (32) and *A. ipaensis* (36). The difference in the number of aquaporin genes in *A. hypogea* can be attributed to gene duplication and loss specific to different subfamilies of AQPs over the course of evolution. Similar observations of gene expansion have been reported in *LEA* and *SWEET* genes in *Brassica napus* (AACC) compared to its progenitors, *Brassica rapa* (AA) and *Brassica oleracea* (CC) [31, 32]. The exon-intron structure observed in the two *Arachis* species was found conserved and does correlate well with their phylogenetic distribution. Since the exon-intron structure in AQP subfamilies was similar in both species, this indicates that a diversification of AQPs preceded the evolution of the genus *Arachis*. The similar gene structure also points to a conserved function of AQPs within the genus. The intron number was reported to be correlated to gene expression, duplication, and diversification in plants [33]. For instance, the high intron number variation in NIPs is correlated with their vulnerability to evolution.

The two conserved NPA motifs along with the four amino acids that form the ar/R SF largely determine solute specificity and transport of the substrate across AQPs [9, 14, 15]. Based on our analyses, the respective members of each AQP subfamily in both *Arachis* species showed conserved NPA motifs and similar ar/R SF. Indeed, all PIPs were found to harbor the characteristic double NPA motif and a hydrophilic ar/R SF (F/H/T/R) as observed in AqpZ [34], confirming their affinity to transport water. The same filter was found conserved for PIP members from other plant species such as *Zea mays* [5], *Solanum lycopersicum* [35], *A. thaliana* [7], *G. max* [30], and *Phaseolus vulgaris* [29]. PIP members are known to regulate water transport in several plant species and play an instrumental role in maintaining root and leaf hydraulics [29]. PIPs have also been shown to regulate photosynthesis in *A. thaliana*, *Hordeum vulgare* and *Nicotiana tabaccum* by facilitating CO_2_ diffusion in mesophyll tissues [29]. Therefore, the conserved features of PIPs suggest similar functions in *Arachis*, a conclusion reinforced by RNA-seq analyses that showed a higher expression of PIPs across different tissues analyzed.

Among TIPs, NPA motifs were conserved and, TIP1s and TIP3s showed more hydrophobic residues than TIP2s, TIP4s and TIP5s. Generally, TIPs are located in vacuolar membranes and act as transporters of water and small solutes like ammonia, hydrogen peroxide_,_ boron and urea [36–39]. The residues found in the ar/R SF in TIP subfamilies were similar to TIPs from other plant species pointing to a similar conserved role in *Arachis* species. A high accumulation of TIP3s was observed in seeds from different developing stages of *A. hypogea*, a phenomenon observed in *A. thaliana* [40, 41], *H. vulgare* [42] and *G. max* [30] and reported as a role in seed desiccation processes. The TIP3s are involved in maturation of the vacuolar apparatus and allow optimal water uptake during embryo development and seed germination [43]. Recently, *BvCOLD1*, a boron transport TIP was found to be involved in cold tolerance in sugar beet [39]. Further studies on TIP regulation could help better understand why cold stress represents a major limitation for peanut cultivation. Interestingly, in a recent study, Devi et al. [44] suggested that putative TIPs and PIPs in *A. hypogea* played a role in regulating drought tolerance.

Among the NIPs, NIP1s showed a selectivity filter with more hydrophobicity (WVAR) compared to NIP2s and NIP3s. In the present study, a single NIP2 gene containing a GSGR selectivity filter was observed in both *Arachis* species. NIP2s with a GSGR selectivity filter play a unique role in plants by allowing influx of silicon (Si) [9, 30]. In turn, Si accumulation has been shown to protect plants against a wide variety of biotic and abiotic stresses [45]. Interestingly, the presence of functional NIP2s for Si permeability vary greatly among plant species and our results bring the first evidence that *A. hypogea* has the appropriate channel to benefit from Si fertilization. In general, NIPs will display lower expression than PIPs or TIPs, and our results confirmed this trend whereAduNIP1–4 and AipNIP1–5 were found specifically expressed in roots and root nodules. A similar specificity of NIP expression was observed in *G. max* [46] and *Medicago truncatula* [47]. Additionally, in *M. truncatula*, NIPs were found to be expressed from the early to late stage of nodule development, which indicates their importance in nodule organogenesis [47].

In the XIP subfamily, a single XIP2 member was found in *A. duranensis*, while members of both XIP1s and XIP2s were observed in *A. ipaensis*. This suggests that *A. duranensis* lost XIP1s during the course of evolution, an observation reported in many other species including all monocots, which raises the question of their importance or role in plants. When they are present, the hydrophobic nature of their selectivity filter is believed to facilitate the transport of hydrophobic and bulky molecules such as urea, glycerol, and boric acid in plants [48]. Interestingly, the expression data showed that the only *A. ipaensis* XIP member, AipXIP2–1, accumulated at higher levels in the nodules of *A. hypogea* supporting its involvement in nodule development. Several studies have established the role of AQPs in key developmental processes [49, 50]. For instance, it has been reported that the increased abundance of TIPs facilitates the development of new lateral root primordia in *A. thaliana* [51]. Nevertheless, XIPs deserve further studies since their exact role and the consequences of their loss in some species, remain poorly understood.

## Conclusions

Genome-wide analysis and characterization of the AQP gene family were performed in *A. duranensis* (AA) and *A. ipaensis* (BB) the probable progenitor genomes of *A. hypogea* (AABB). The identification and the phylogenetic analysis of AQPs in both species revealed the presence of all five sub-families of AQPs. Within the XIP subfamily, the loss of XIP1s from the AA genome was observed, while the presence of a NIP2 in both genomes support a conserved ability to absorb Si within plants of the genus. The global expression profile of AQPs in *A. hypogea* through RNA-seq data analysis revealed a similar pattern of expression of AQPs regardless of the subfamilies or the genomes. A higher expression of TIP3s was observed in different stages of seed development in *A. hypogea* supporting a critical physiological role of TIP3s in seed development. The high accumulation of the BB genome specific-AipXIP2–1 in nodules of *A. hypogea* suggests a novel role in nodule development for the elusive XIPs. The AQPs identified in the present study will serve as a resource for further characterization and possible exploitation of AQPs to understand their physiological role in *A. hypogea*.

## Methods

### Genome-wide identification and distribution of AQPs in *A. duranensis* and *A. ipaensis*

The genome sequences of *A. duranensis A. ipaensis* and *A. hypogea* were retrieved from the PeanutBase (https://peanutbase.org/) [52]. Predicted protein sequences were used to create a local database using BioEdit ver. 7.2.5 [53]. Homologs of the AQPs coding genes were identified by BLASTp search performed against the local database using AQPs from *A. thaliana*, *Oryza sativa* and *G. max* (Additional file 6). An e-value of 10^− 5^ was kept as an initial cut-off to identify high scoring pairs (HSPs). The blast output was tabulated, and the HSPs having greater than 100-bit score were selected. Finally, redundant hits were removed to select unique sequences for further analysis.

### Structural characterization of *A. duranensis* and *A. ipaensis* AQPs

The genomic and cDNA sequences of AQPs identified in *A. duranensis* and *A.ipaensis* were retrieved from PeanutBase. Structural annotations of the gene models (in gff3 format) were also retrieved from PeanutBase. The gene structure of AQPs was analyzed using GSDS ver. 2.0 [54].

### Identification of functional motif and transmembrane domains and estimation of isoelectric point (pI) for AQPs

The NPA motifs were identified in predicted protein sequences using conserved domain database (CDD, www.ncbi.nlm.nih.gov/Structure/cdd/cdd.shtml) [55]. Missing NPA motifs in few AQP sequences were confirmed with a manual examination. Transmembrane domains in the genes were identified using TMHMM (http://www.cbs.dtu.dk/services/TMHMM/) [56] and SOSUI online software tools [57]. The transmembrane domains were analyzed manually to confirm alterations or complete loss. The isoelectric point (pI) of AQP protein sequences were calculated using the online tool Sequence Manipulation Suite version 2 (http://www.bioinformatics.org/sms2/protein_iep.html) [58].

### Phylogenetic analysis of AQPs in *A. duranensis* and *A. ipaensis*

The predicted AQP protein sequences were aligned using CLUSTALW alignment tool in MEGA6 [59]. The phylogenetic tree was constructed by the maximum likelihood method, and the stability of the branch node was analysed by performing 1000 bootstraps. The AQP subfamilies, PIP, SIP, TIP, NIP, and XIP, were classified according to the nomenclature used for *A. thaliana*, and *G. max* [6, 9].

### Tertiary protein structure prediction

The tertiary (3D) protein structure of *A. duranensis* and *A. ipaensis* AQPs were generated using the Phyre2 protein-modeling server (http://www.sbg.bio.ic.ac.uk/~phyre2) [60] with extensive mode. Identification of transmembrane pore, pore lining residues, pore morphology and constricts in the 3D protein structures were performed using PoreWalker server (http://www.ebi.ac.uk/thornton-srv/software/PoreWalker/) [61].

### Expression profiling of *A. hypogea* AQPs

RNA-seq data derived from 22 different tissues of cultivated peanut available in PeanutBase (Genbank BioProject PRJNA291488) were used for expression analysis. The transcriptome assembly and expression value estimation were done as described in Clevenger et al. [62]. Briefly, de novo assembly was carried out by a genome-guided approach using assembly pipeline from Trinity [63]. Total reads were mapped to the transcript assembly from 58 libraries using Bowtie [64], allowing two mismatches within a particular 25 bp seed. Uniquely mapped raw read counts per gene were normalized using the formula of Reads Per Kilobase of transcript per Million mapped reads (RPKM) = Number of Reads / (Gene Length/1000 * Total Number of Reads/1,000,000). The RPKM values for AQPs were extracted and used for heat map preparation. A heat map was constructed using TIGR Multi Experiment Viewer (MeV,http://mev.tm4.org). Hierarchical clustering with average linkage method was performed to cluster the AQPs.

## Additional Files

Additional file 1: Conserved domain analysis of aquaporins identified in *Arachis duranensis* and *Arachis ipaensis* using CDD tool from NCBI (DOCX 19 kb)

Additional file 2: Number of aquaporins identified in *Arachis duranensis*, *Arachis ipaensis* and *Arachis hypogea* genome. (DOCX 13 kb)

Additional file 3: Transmembrane domains in aquaporins identified in *Arachis duranensis* and *Arachis ipaensis* using TMHMM and SOSUI servers (DOCX 22 kb)

Additional file 4: Predicted tertiary (3D) protein structure of *Arachis duranensis* and *Arachis ipaensis* aquaporins (DOCX 7066 kb)

Additional file 5: Details of predicted sub-cellular location of *Arachis duranensis* aquaporins identified by using Wolfpsort server (DOCX 16 kb)

Additional file 6: Amino acid sequences of aquaporins from *Arabidopsis thaliana*, *Glycine max* and *Oryza sativa* used for BLASTp search. Sequence names of *A. thaliana*, *G. max* and *O. sativa* are preceded by the prefixes At, Gm and Os respectively. (DOCX 27 kb)

## Abbreviations

AQP: Aquaporin
ar/R: Aromatic/arginine
NIP: Nodulin26-like Intrinsic Proteins
NPA: Asparagine-proline-alanine
PIP: Plasma membrane Intrinsic Protein
SF: Selectivity Filter
SIP: Small basic Intrinsic Proteins
TIP: Tonoplast Intrinsic Protein
TM: Transmembrane helix
XIP: X Intrinsic Proteins
