# Supplementary material for: Identification and characterization of aquaporin genes in Arachis duranensis and Arachis ipaensis genomes, the diploid progenitors of peanut
Source: BMC Genomics. 2019 Mar 18;20:222. doi: 10.1186/s12864-019-5606-4 (PMC6423786; doi:10.1186/s12864-019-5606-4)
Supplement: Supplementary file 1 — Conserved domain analysis of aquaporins identified in Arachis duranensis and Arachis ipaensis using CDD tool from NCBI (DOCX 19 kb) [file 12864_2019_5606_MOESM1_ESM.docx]

**Additional file 1**

Conserved domain analysis of aquaporins identified in *Arachis duranensis* using CDD tool from NCBI

| **Query** | **PSSM-ID** | **E-Value** | **Bitscore** | **Accession** | **Short name** | **Superfamily** |
| --- | --- | --- | --- | --- | --- | --- |
| AduPIP1-1 | 278651 | 2.34E-98 | 287.677 | pfam00230 | MIP | cl00200 |
| AduPIP1-2 | 278651 | 2.34E-98 | 287.677 | pfam00230 | MIP | cl00200 |
| AduPIP1-3 | 278651 | 2.24E-79 | 236.445 | pfam00230 | MIP | cl00200 |
| AduPIP1-4 | 278651 | 2.30E-98 | 288.062 | pfam00230 | MIP | cl00200 |
| AduPIP1-5 | 278651 | 8.26E-98 | 286.521 | pfam00230 | MIP | cl00200 |
| AduPIP2-1 | 278651 | 8.47E-98 | 286.136 | pfam00230 | MIP | cl00200 |
| AduPIP2-2 | 278651 | 3.11E-97 | 284.98 | pfam00230 | MIP | cl00200 |
| AduPIP2-3 | 278651 | 2.65E-90 | 266.876 | pfam00230 | MIP | cl00200 |
| AduPIP2-4 | 278651 | 7.12E-95 | 278.817 | pfam00230 | MIP | cl00200 |
| AduTIP1-1 | 177664 | 5.27E-138 | 387.605 | PLN00027 | PLN00027 | cl00200 |
| AduTIP1-2 | 177664 | 1.44E-124 | 356.018 | PLN00027 | PLN00027 | cl00200 |
| AduTIP1-3 | 177664 | 2.87E-109 | 312.106 | PLN00027 | PLN00027 | cl00200 |
| AduTIP1-4 | 177664 | 8.99E-142 | 397.235 | PLN00027 | PLN00027 | cl00200 |
| AduTIP2-1 | 294134 | 2.18E-103 | 299.93 | cl00200 | MIP superfamily | - |
| AduTIP2-2 | 294134 | 2.05E-120 | 343.072 | cl00200 | MIP superfamily | - |
| AduTIP2-3 | 294134 | 5.91E-110 | 316.108 | cl00200 | MIP superfamily | - |
| AduTIP3-1 | 294134 | 6.91E-101 | 294.001 | cl00200 | MIP superfamily | - |
| AduTIP4-1 | 294134 | 4.00E-78 | 235.836 | cl00200 | MIP superfamily | - |
| AduTIP4-2 | 294134 | 2.09E-78 | 236.992 | cl00200 | MIP superfamily | - |
| AduTIP5-1 | 294134 | 3.08E-125 | 356.52 | cl00200 | MIP superfamily | - |
| AduNIP1-1 | 294134 | 4.41E-123 | 351.904 | cl00200 | MIP superfamily | - |
| AduNIP1-2 | 294134 | 5.78E-104 | 303.866 | cl00200 | MIP superfamily | - |
| AduNIP1-3 | 294134 | 2.28E-130 | 370.008 | cl00200 | MIP superfamily | - |
| AduNIP1-4 | 294134 | 6.16E-123 | 352.016 | cl00200 | MIP superfamily | - |
| AduNIP1-5 | 294134 | 1.16E-111 | 323.897 | cl00200 | MIP superfamily | - |
| AduNIP2-1 | 294134 | 1.32E-70 | 219.734 | cl00200 | MIP superfamily | - |
| AduNIP3-1 | 177663 | 7.78E-170 | 472.81 | PLN00026 | PLN00026 | cl00200 |
| AduNIP3-2 | 177663 | 2.05E-157 | 441.223 | PLN00026 | PLN00026 | cl00200 |
| AduSIP1-1 | 294134 | 2.93E-16 | 74.9826 | cl00200 | MIP superfamily | - |
| AduSIP1-2 | 294134 | 1.53E-11 | 61.8858 | cl00200 | MIP superfamily | - |
| AduSIP2-1 | 294134 | 1.70E-07 | 50.32 | cl00200 | MIP superfamily | - |
| AduXIP2-1 | 294134 | 1.48E-22 | 93.1508 | cl00200 | MIP superfamily | - |

Conserved domain analysis of aquaporins identified in *Arachis ipaensis* using CDD tool from NCBI

| **Query** | **PSSM-ID** | **E-Value** | **Bitscore** | **Accession** | **Short name** | **Superfamily** |
| --- | --- | --- | --- | --- | --- | --- |
| AipPIP1-1 | 278651 | 8.33E-99 | 288.832 | pfam00230 | MIP | cl00200 |
| AipPIP1-2 | 278651 | 4.90E-94 | 276.121 | pfam00230 | MIP | cl00200 |
| AipPIP1-3 | 278651 | 5.90E-100 | 291.914 | pfam00230 | MIP | cl00200 |
| AipPIP1-4 | 278651 | 1.30E-98 | 288.447 | pfam00230 | MIP | cl00200 |
| AipPIP1-5 | 278651 | 8.76E-99 | 288.832 | pfam00230 | MIP | cl00200 |
| AipPIP2-1 | 278651 | 6.24E-98 | 286.521 | pfam00230 | MIP | cl00200 |
| AipPIP2-2 | 278651 | 1.02E-97 | 286.136 | pfam00230 | MIP | cl00200 |
| AipPIP2-3 | 278651 | 9.46E-95 | 278.432 | pfam00230 | MIP | cl00200 |
| AipPIP2-4 | 278651 | 3.69E-100 | 293.069 | pfam00230 | MIP | cl00200 |
| AipTIP1-1 | 177664 | 3.05E-141 | 396.079 | PLN00027 | PLN00027 | cl00200 |
| AipTIP1-2 | 294134 | 2.96E-99 | 287.453 | cl00200 | MIP superfamily | - |
| AipTIP1-3 | 177664 | 3.87E-142 | 398.39 | PLN00027 | PLN00027 | cl00200 |
| AipTIP2-1 | 294134 | 5.03E-117 | 334.598 | cl00200 | MIP superfamily | - |
| AipTIP2-2 | 294134 | 2.69E-104 | 302.241 | cl00200 | MIP superfamily | - |
| AipTIP2-3 | 294134 | 1.18E-121 | 346.154 | cl00200 | MIP superfamily | - |
| AipTIP3-1 | 177664 | 5.38E-102 | 297.853 | PLN00027 | PLN00027 | cl00200 |
| AipTIP4-1 | 294134 | 1.80E-77 | 234.295 | cl00200 | MIP superfamily | - |
| AipTIP4-2 | 294134 | 3.55E-67 | 206.946 | cl00200 | MIP superfamily | - |
| AipTIP5-1 | 294134 | 1.24E-124 | 354.98 | cl00200 | MIP superfamily | - |
| AipNIP1-1 | 294134 | 2.19E-101 | 295.665 | cl00200 | MIP superfamily | - |
| AipNIP1-2 | 294134 | 2.18E-130 | 370.394 | cl00200 | MIP superfamily | - |
| AipNIP1-3 | 294134 | 1.33E-104 | 305.407 | cl00200 | MIP superfamily | - |
| AipNIP1-4 | 294134 | 3.96E-112 | 325.052 | cl00200 | MIP superfamily | - |
| AipNIP1-5 | 294134 | 1.36E-120 | 345.083 | cl00200 | MIP superfamily | - |
| AipNIP2-1 | 294134 | 2.47E-72 | 225.897 | cl00200 | MIP superfamily | - |
| AipNIP3-1 | 177663 | 3.99E-171 | 475.891 | PLN00026 | PLN00026 | cl00200 |
| AipNIP3-2 | 294134 | 1.25E-36 | 130.367 | cl00200 | MIP superfamily | - |
| AipNIP3-3 | 177663 | 2.72E-138 | 440.838 | PLN00026 | PLN00026 | cl00200 |
| AipNIP4-1 | 294134 | 5.65E-80 | 238.218 | cl00200 | MIP superfamily | - |
| AipSIP1-1 | 294134 | 6.02E-17 | 76.5234 | cl00200 | MIP superfamily | - |
| AipSIP1-2 | 294134 | 5.60E-12 | 63.0414 | cl00200 | MIP superfamily | - |
| AipSIP2-1 | 294134 | 2.72E-08 | 52.1818 | cl00200 | MIP superfamily | - |
| AipXIP1-1 | 294134 | 3.49E-23 | 92.7656 | cl00200 | MIP superfamily | - |
| AipXIP1-2 | 294134 | 4.97E-06 | 46.1565 | cl00200 | MIP superfamily | - |
| AipXIP1-3 | 294134 | 0.000198 | 45.77 | cl00200 | MIP superfamily | - |
| AipXIP2-1 | 294134 | 4.29E-23 | 95.3981 | cl00200 | MIP superfamily | - |
