# Supplementary material for: Identification and characterization of aquaporin genes in Arachis duranensis and Arachis ipaensis genomes, the diploid progenitors of peanut
Source: BMC Genomics. 2019 Mar 18;20:222. doi: 10.1186/s12864-019-5606-4 (PMC6423786; doi:10.1186/s12864-019-5606-4)
Supplement: Supplementary file 2 — Number of aquaporins identified in Arachis duranensis, Arachis ipaensis and Arachis hypogea genome. (DOCX 13 kb) [file 12864_2019_5606_MOESM2_ESM.docx]

**Additional File 2**

Number of aquaporins identified in *Arachis duranensis*, *Arachis ipaensis* and *Arachis hypogea* genome.

|  | *Arachis duranensis* | *Arachis ipaensis* | *Arachis hypogea* |
| --- | --- | --- | --- |
| NIP1s | 5 | 5 | 10 |
| NIP2s | 1 | 1 | 3 |
| NIP3s | 2 | 3 | 7 |
| NIP4s | 0 | 1 | 3 |
| PIP1s | 5 | 5 | 7 |
| PIP2s | 4 | 4 | 8 |
| SIP2s | 2 | 2 | 4 |
| SIP2s | 1 | 1 | 2 |
| TIP1s | 4 | 3 | 10 |
| TIP2s | 3 | 3 | 6 |
| TIP3s | 1 | 1 | 2 |
| TIP4s | 2 | 2 | 4 |
| TIP5s | 1 | 1 | 2 |
| XIP1s | 0 | 3 | 3 |
| XIP2s | 1 | 1 | 2 |
| **Total number of AQPs** | **32** | **36** | **73** |
