# Supplementary material for: Identification and characterization of aquaporin genes in Arachis duranensis and Arachis ipaensis genomes, the diploid progenitors of peanut
Source: BMC Genomics. 2019 Mar 18;20:222. doi: 10.1186/s12864-019-5606-4 (PMC6423786; doi:10.1186/s12864-019-5606-4)
Supplement: Supplementary file 3 — Transmembrane domains in aquaporins identified in Arachis duranensis and Arachis ipaensis using TMHMM and SOSUI servers (DOCX 22 kb) [file 12864_2019_5606_MOESM3_ESM.docx]

**Additional file 3**

Transmembrane domains in aquaporins identified in *Arachis duranensis* using TMHMM and SOSUI servers

|  |  |  | **TMHMM** |  |  | **SOSUI** |  |  |
| --- | --- | --- | --- | --- | --- | --- | --- | --- |
| **gene** | **Length** | **ExpAA** | **First 60** | **predHel** |  | **protein type** | **helix** | **Max TM** |
| AduPIP1-1 | 288 | 129.96 | 3.55 | 6 |  | MP | 5 | **6** |
| AduPIP1-2 | 288 | 129.96 | 3.55 | 6 |  | MP | 5 | **6** |
| AduPIP1-3 | 215 | 104.97 | 26.61 | 4 |  | MP | 5 | **5** |
| AduPIP1-4 | 298 | 129.83 | 0.03 | 6 |  | MP | 5 | **6** |
| AduPIP1-5 | 289 | 127.56 | 4.43 | 6 |  | MP | 6 | **6** |
| AduPIP2-1 | 287 | 132.09 | 20.76 | 6 |  | MP | 5 | **6** |
| AduPIP2-2 | 287 | 135.6 | 20.76 | 6 |  | MP | 5 | **6** |
| AduPIP2-3 | 279 | 130.79 | 15.31 | 6 |  | MP | 6 | **6** |
| AduPIP2-4 | 289 | 129.09 | 18.08 | 6 |  | MP | 5 | **6** |
| AduTIP1-1 | 250 | 147.48 | 25.58 | 7 |  | MP | 6 | **7** |
| AduTIP1-2 | 301 | 122.4 | 0.06 | 5 |  | MP | 6 | **6** |
| AduTIP1-3 | 187 | 110.64 | 41.28 | 5 |  | MP | 4 | **5** |
| AduTIP1-4 | 252 | 142.99 | 26.02 | 7 |  | MP | 6 | **7** |
| AduTIP2-1 | 248 | 153.27 | 27.95 | 7 |  | MP | 7 | **7** |
| AduTIP2-2 | 248 | 139.85 | 28.85 | 6 |  | MP | 6 | **6** |
| AduTIP2-3 | 236 | 138.21 | 29.26 | 6 |  | MP | 6 | **6** |
| AduTIP3-1 | 255 | 130.82 | 17.47 | 6 |  | MP | 5 | **6** |
| AduTIP4-1 | 247 | 150.75 | 31.31 | 7 |  | MP | 7 | **7** |
| AduTIP4-2 | 258 | 150.78 | 31.11 | 7 |  | MP | 7 | **7** |
| AduTIP5-1 | 271 | 166.11 | 39.51 | 8 |  | MP | 8 | **8** |
| AduNIP1-1 | 271 | 133.45 | 19.61 | 6 |  | MP | 6 | **6** |
| AduNIP1-2 | 266 | 119.96 | 20.65 | 5 |  | MP | 6 | **6** |
| AduNIP1-3 | 263 | 133.23 | 16.55 | 6 |  | MP | 6 | **6** |
| AduNIP1-4 | 263 | 130.9 | 21.67 | 6 |  | MP | 6 | **6** |
| AduNIP1-5 | 278 | 119.54 | 13.3 | 5 |  | MP | 6 | **6** |
| AduNIP2-1 | 285 | 127.52 | 20.78 | 6 |  | MP | 6 | **6** |
| AduNIP3-1 | 310 | 129.6 | 0.02 | 6 |  | MP | 6 | **6** |
| AduNIP3-2 | 307 | 121.07 | 0.01 | 5 |  | MP | 6 | **6** |
| AduSIP1-1 | 245 | 134.11 | 33.83 | 6 |  | MP | 6 | **6** |
| AduSIP1-2 | 249 | 133.12 | 32.22 | 6 |  | MP | 5 | **6** |
| AduSIP2-1 | 240 | 112.82 | 40.05 | 4 |  | MP | 4 | **4** |
| AduXIP2-1 | 312 | 133.58 | 13.43 | 6 |  | MP | 6 | **6** |

ExpAA: The expected number of amino acids in transmembrane helices.

First60: The expected number of amino acids in transmembrane helices in the first 60 amino acids of the protein.

MP: Membrane protein

TMH: The number of predicted transmembrane helices.

MAX TMH: The maximum number among the TMH predicted by TMHMM and SOSUI

^#^ indicates sequences showing less than 6 transmembrane domains based on their sequence alignment.

Transmembrane domains in aquaporins identified in *Arachis ipaensis* using TMHMM and SOSUI servers

|  |  |  | **TMHMM** |  |  | **SOSUI** |  |  |
| --- | --- | --- | --- | --- | --- | --- | --- | --- |
| **gene** | **Length** | **ExpAA** | **First 60** | **predHel** |  | **protein type** | **helix** | **Max TM** |
| AipPIP1-1 | 289 | 127.83 | 4.42 | 6 |  | MP | 6 | 6 |
| AipPIP1-2 | 273 | 128.78 | 18.31 | 6 |  | MP | 5 | 6 |
| AipPIP1-3 | 294 | 129.26 | 1.2 | 6 |  | MP | 5 | 6 |
| AipPIP1-4 | 289 | 129.56 | 4.12 | 6 |  | MP | 5 | 6 |
| AipPIP1-5 | 288 | 129.98 | 3.52 | 6 |  | MP | 5 | 6 |
| AipPIP2-1 | 287 | 135.83 | 20.76 | 6 |  | MP | 5 | 6 |
| AipPIP2-2 | 287 | 132.1 | 20.76 | 6 |  | MP | 5 | 6 |
| AipPIP2-3 | 289 | 129.24 | 18.05 | 6 |  | MP | 5 | 6 |
| AipPIP2-4 | 310 | 129.64 | 15.33 | 6 |  | MP | 4 | 6 |
| AipTIP1-1 | 251 | 144.86 | 26.46 | 7 |  | MP | 6 | 7 |
| AipTIP1-2 | 197 | 110.88 | 25.92 | 5 |  | MP | 5 | 5 |
| AipTIP1-3 | 252 | 143.26 | 26.11 | 7 |  | MP | 7 | 7 |
| AipTIP2-1 | 248 | 151.62 | 29.46 | 6 |  | MP | 6 | 6 |
| AipTIP2-2 | 248 | 153 | 27.81 | 7 |  | MP | 7 | 7 |
| AipTIP2-3 | 248 | 139.88 | 28.83 | 6 |  | MP | 6 | 6 |
| AipTIP3-1 | 282 | 131.37 | 17.85 | 6 |  | MP | 5 | 6 |
| AipTIP4-1 | 247 | 142.56 | 30.84 | 7 |  | MP | 7 | 7 |
| AipTIP4-2 | 222 | 130.45 | 32.59 | 6 |  | MP | 6 | 6 |
| AipTIP5-1 | 271 | 164.44 | 38.99 | 8 |  | MP | 8 | 8 |
| AipNIP1-1 | 243 | 113.25 | 25.51 | 5 |  | MP | 5 | 5 |
| AipNIP1-2 | 263 | 133.48 | 16.64 | 6 |  | MP | 6 | 6 |
| AipNIP1-3 | 260 | 121.4 | 21.16 | 5 |  | MP | 6 | 6 |
| AipNIP1-4 | 274 | 120.54 | 17.25 | 5 |  | MP | 6 | 6 |
| AipNIP1-5 | 242 | 125.49 | 40.07 | 5 |  | MP | 6 | 6 |
| AipNIP2-1 | 337 | 147.3 | 20.56 | 6 |  | MP | 6 | 6 |
| AipNIP3-1 | 310 | 129.72 | 0.02 | 6 |  | MP | 6 | 6 |
| AipNIP3-2 | 242 | 79.23 | 0.1 | 4 |  | MP | 4 | 4 |
| AipNIP3-3 | 307 | 122.12 | 0.01 | 5 |  | MP | 6 | 6 |
| AipNIP4-1 | 176 | 120.49 | 38.19 | 5 |  | MP | 5 | 5 |
| AipSIP1-1 | 229 | 121.52 | 31.74 | 5 |  | MP | 6 | 6 |
| AipSIP1-2 | 249 | 133.26 | 32.18 | 6 |  | MP | 5 | 6 |
| AipSIP2-1 | 240 | 113.84 | 40.93 | 4 |  | MP | 5 | 5 |
| AipXIP1-1 | 226 | 106.32 | 24.14 | 5 |  | MP | 5 | 5 |
| AipXIP1-2 | 242 | 109.01 | 21.39 | 5 |  | MP | 5 | 5 |
| AipXIP1-3 | 288 | 85.23 | 0 | 3 |  | MP | 4 | 4 |
| AipXIP2-1 | 350 | 129.45 | 0.01 | 6 |  | MP | 6 | 6 |

ExpAA: The expected number of amino acids in transmembrane helices.

First60: The expected number of amino acids in transmembrane helices in the first 60 amino acids of the protein.

MP: Membrane protein

TMH: The number of predicted transmembrane helices.

MAX TMH: The maximum number among the TMH predicted by TMHMM and SOSUI

^#^ indicates sequences showing less than 6 transmembrane domains based on their sequence alignment.
