# Supplementary material for: Identification and characterization of aquaporin genes in Arachis duranensis and Arachis ipaensis genomes, the diploid progenitors of peanut
Source: BMC Genomics. 2019 Mar 18;20:222. doi: 10.1186/s12864-019-5606-4 (PMC6423786; doi:10.1186/s12864-019-5606-4)
Supplement: Supplementary file 4 — Predicted tertiary (3D) protein structure of Arachis duranensis and Arachis ipaensis aquaporins (DOCX 7066 kb) [file 12864_2019_5606_MOESM4_ESM.docx]

| 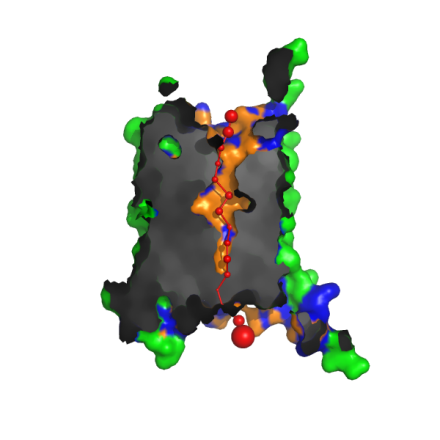 | 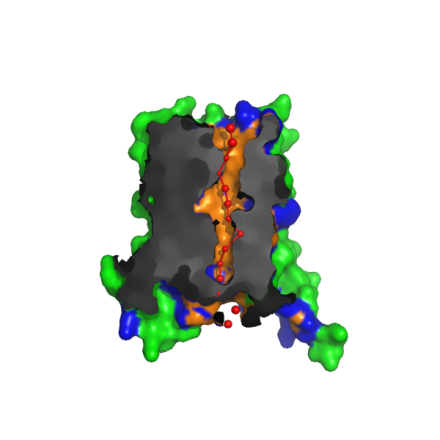 | 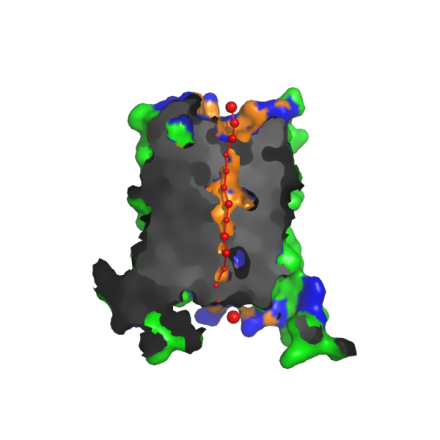 |
| --- | --- | --- |
| AduNIP1-1 | AduNIP1-2 | AduNIP1-3 |
| 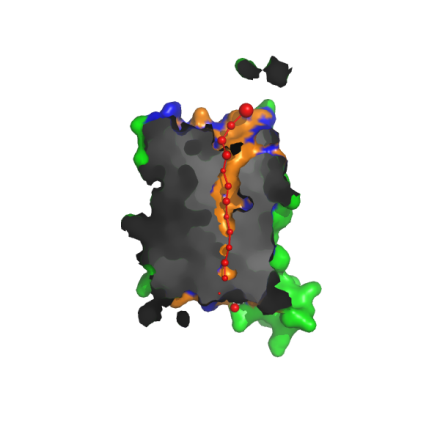 | 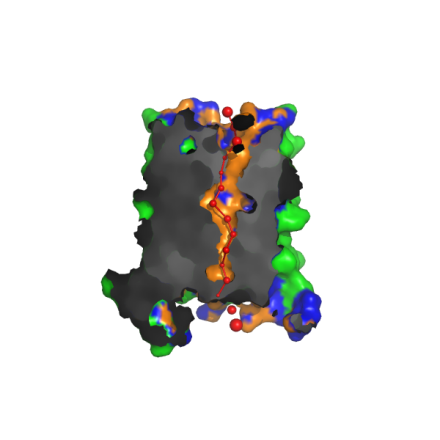 | 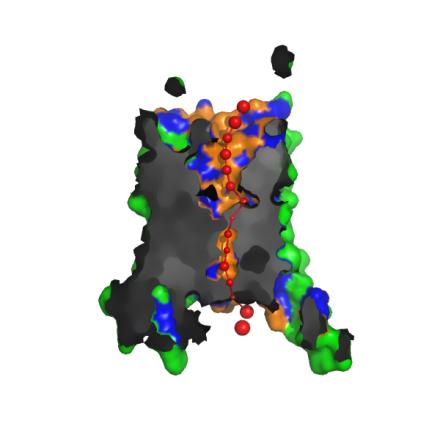 |
| AduNIP1-4 | AduNIP1-5 | AduNIP2-1 |
| 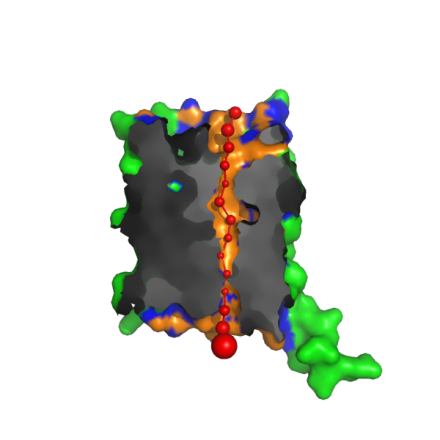 | 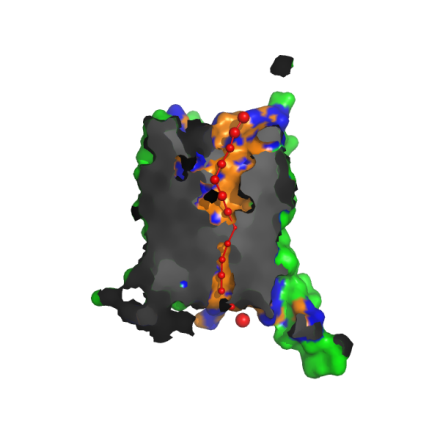 | 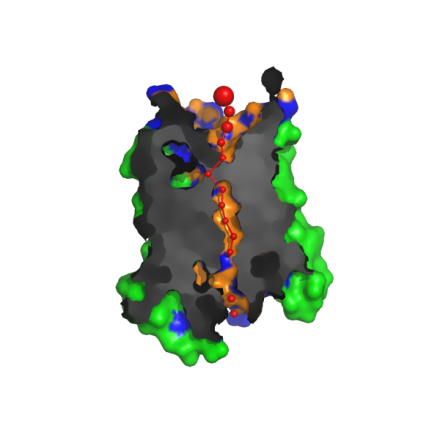 |
| AduNIP3-1 | AduNIP3-2 | AduPIP1-1 |

**Additional file 4:** Predicted tertiary (3D) protein structure of *Arachis duranensis* and *Arachis ipaensis* aquaporins

| 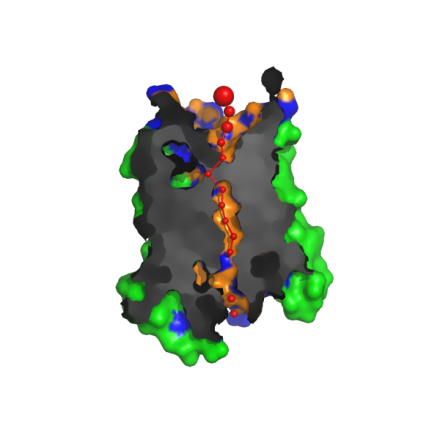 | 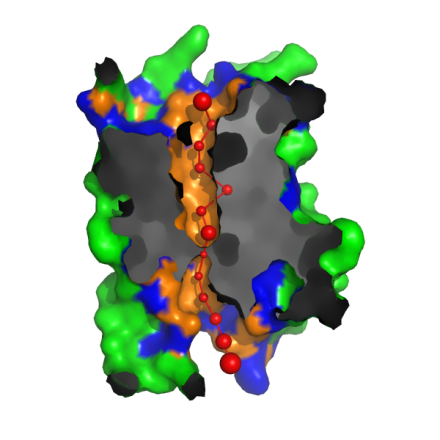 | 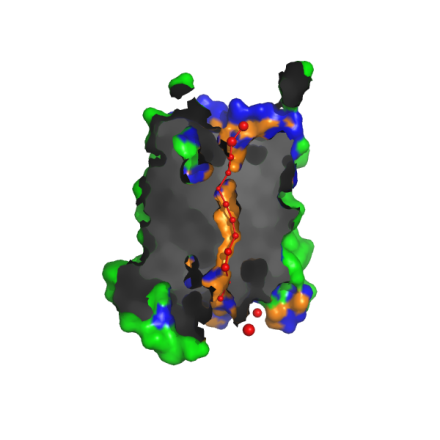 |
| --- | --- | --- |
| AduPIP1-2 | AduPIP1-3 | AduPIP1-4 |
| 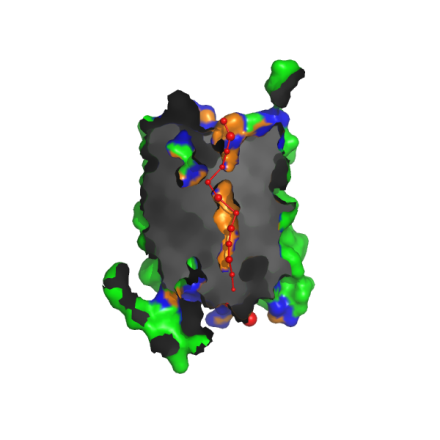 | 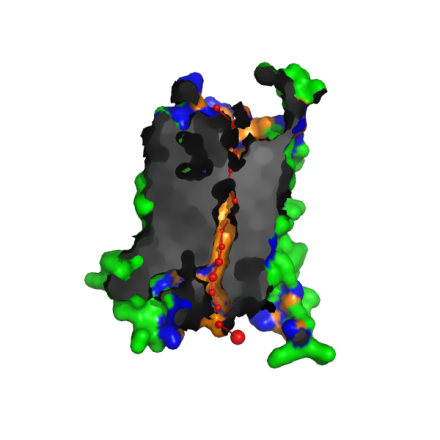 | 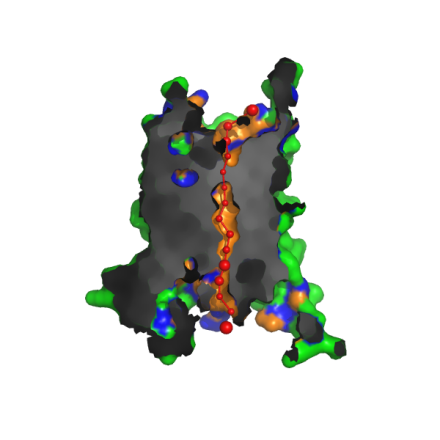 |
| AduPIP1-5 | AduPIP2-1 | AduPIP2-2 |
| 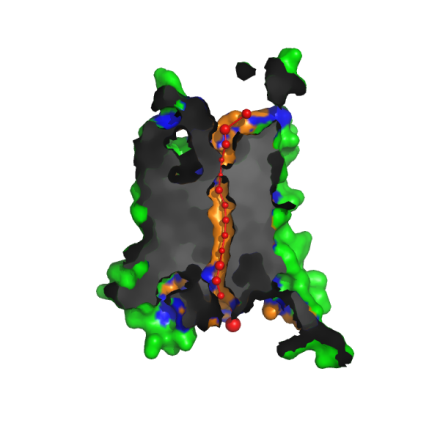 | 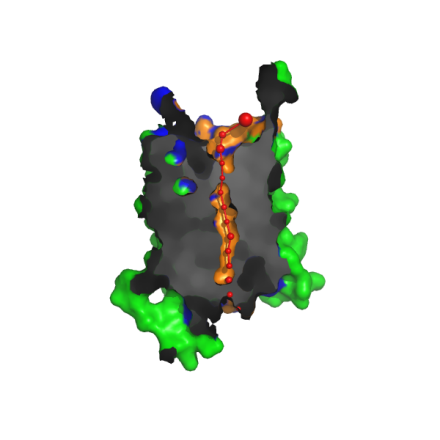 | 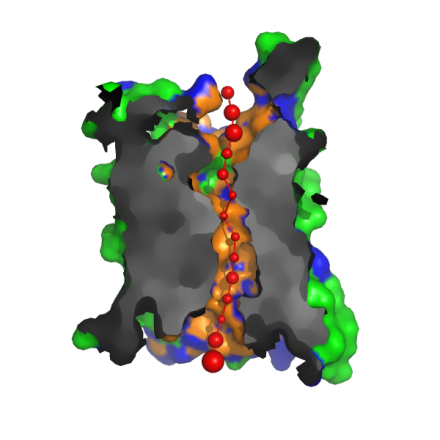 |
| AduPIP2-3 | AduPIP2-4 | AduSIP1-1 |

| 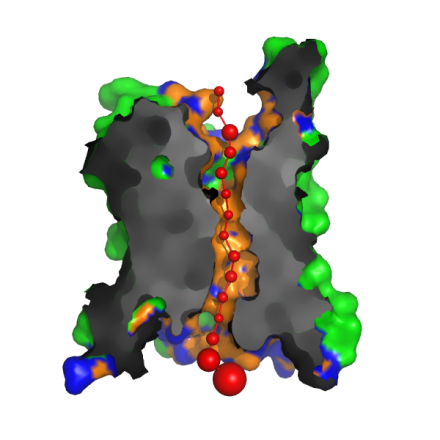 | 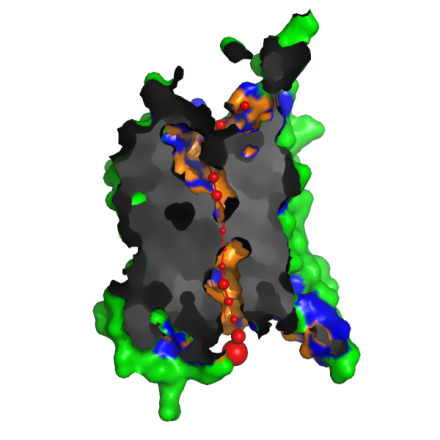 | 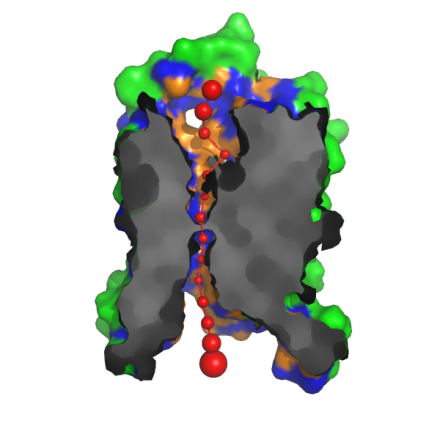 |
| --- | --- | --- |
| AduSIP1-2 | AduSIP2-1 | AduTIP1-1 |
| 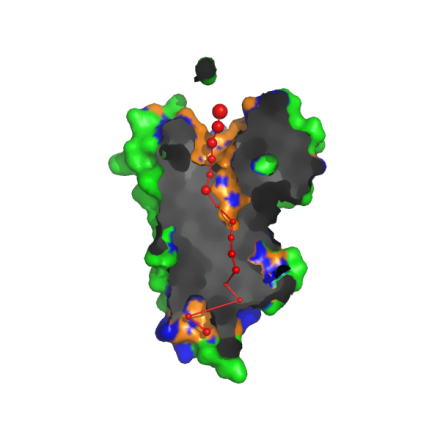 | 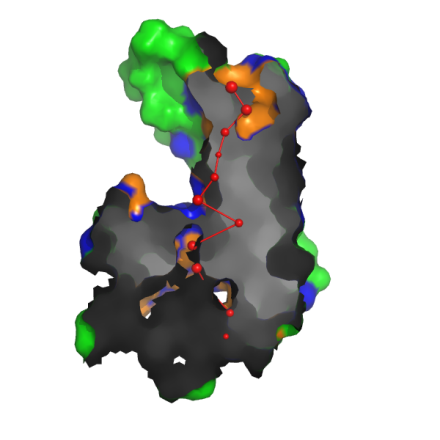 | 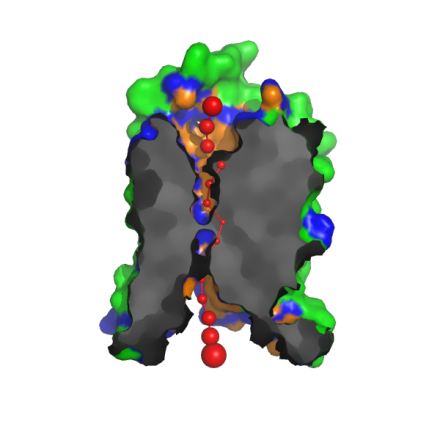 |
| AduTIP1-2 | AduTIP1-3 | AduTIP1-4 |
| 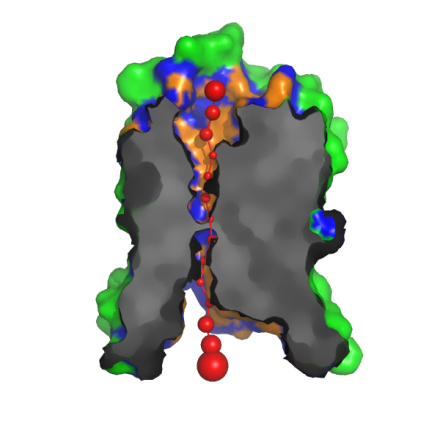 | 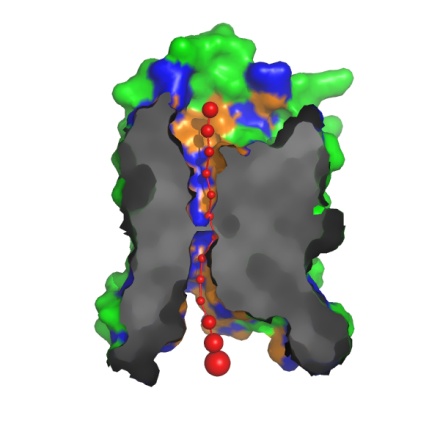 | 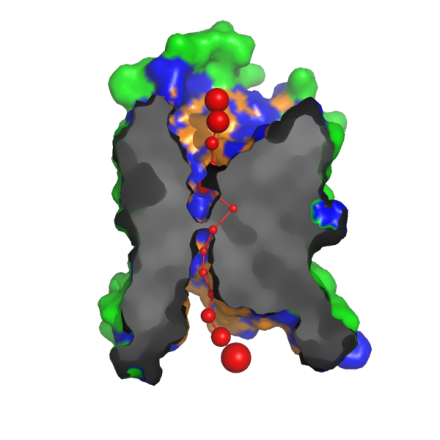 |
| AduTIP2-1 | AduTIP2-2 | AduTIP2-3 |

| 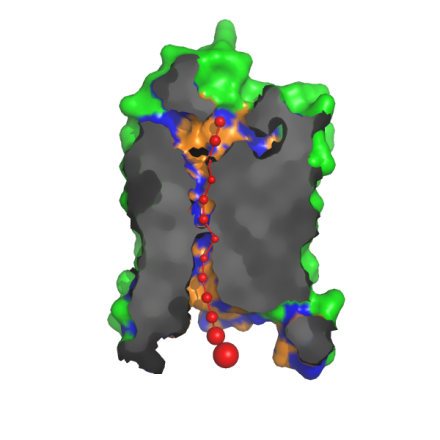 | 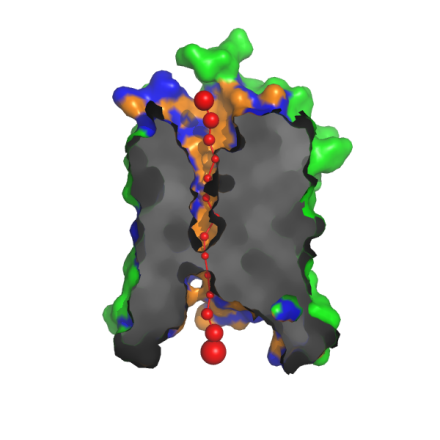 | 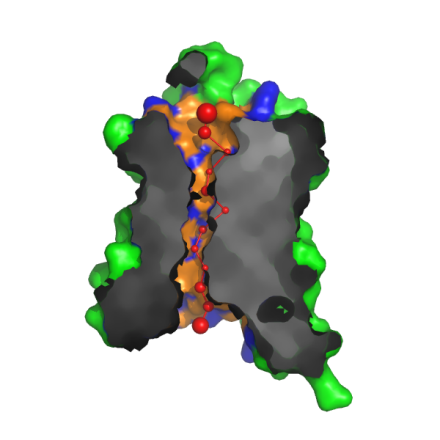 |
| --- | --- | --- |
| AduTIP3-1 | AduTIP4-1 | AduTIP4-2 |
| 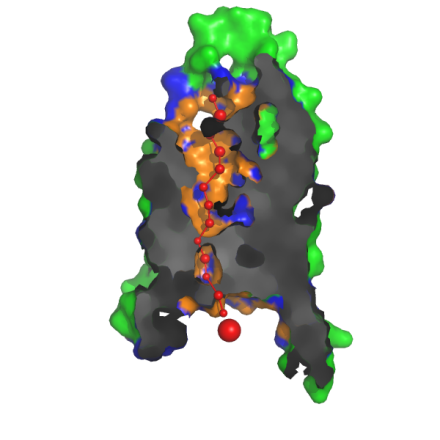 | 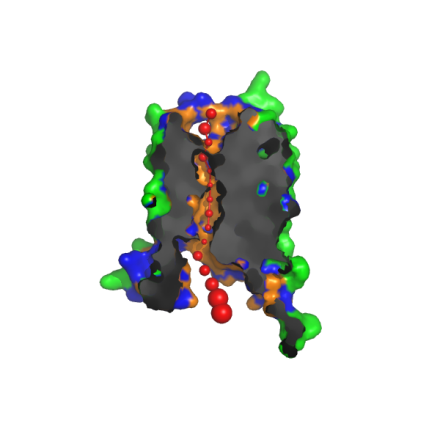 |  |
| AduTIP5-1 | AduXIP2-1 |  |

| 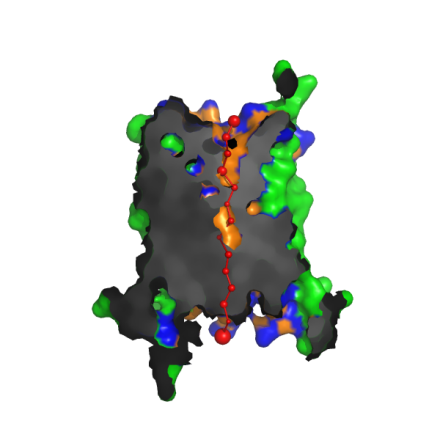 | 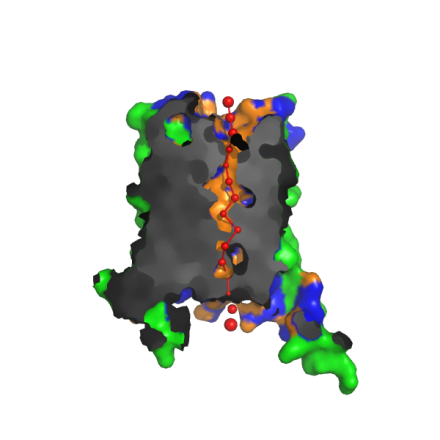 | 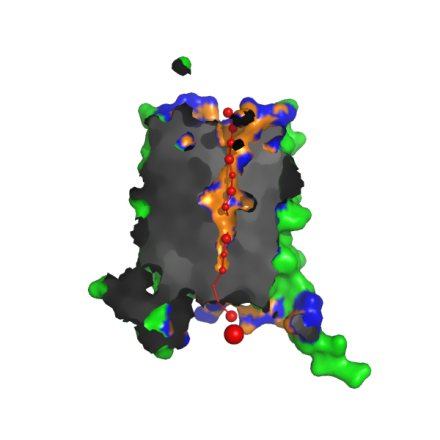 |
| --- | --- | --- |
| AipNIP1-1 | AipNIP1-2 | AipNIP1-4 |
| 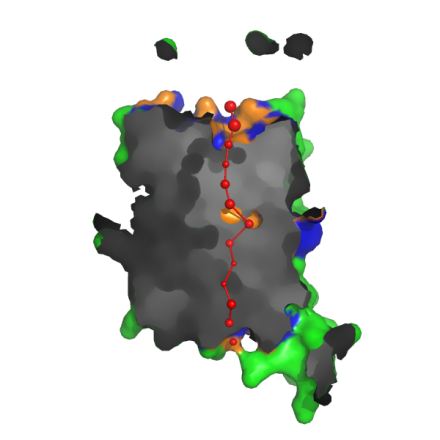 | 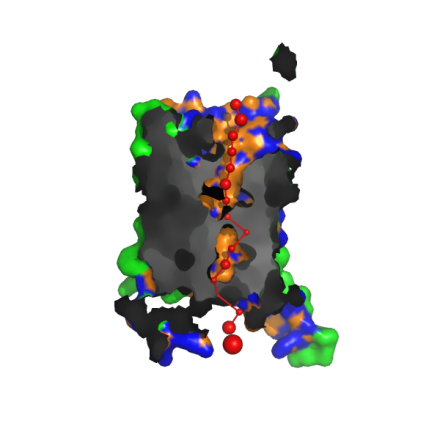 | 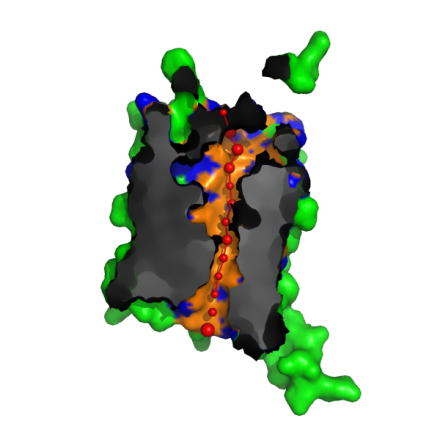 |
| AipNIP1-5 | AipNIP2-1 | AipNIP3-1 |
| 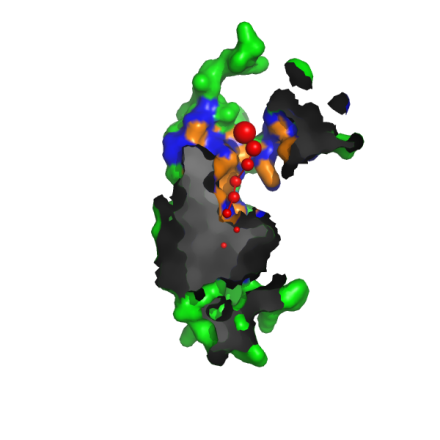 | 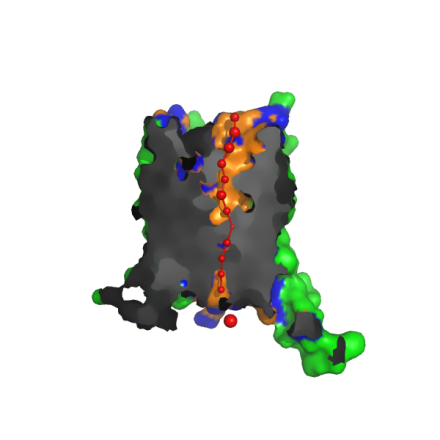 | 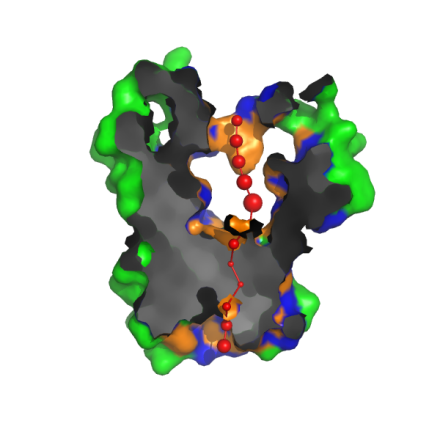 |
| AipNIP3-2 | AipNIP3-3 | AipNIP4-1 |

| 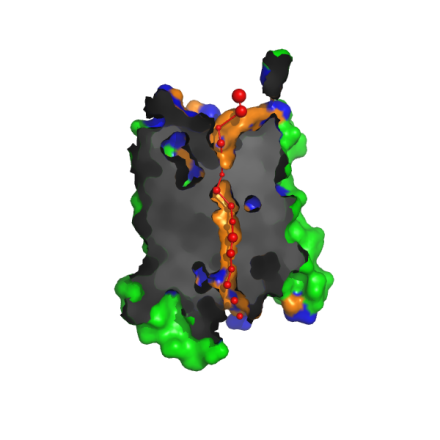 | 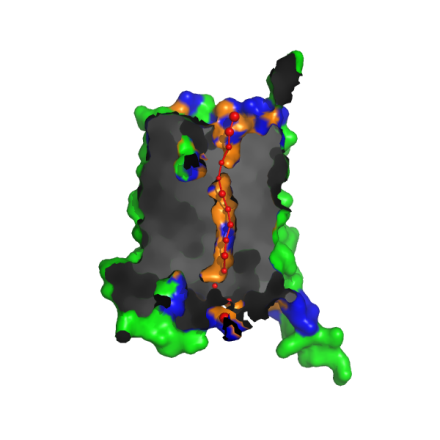 | 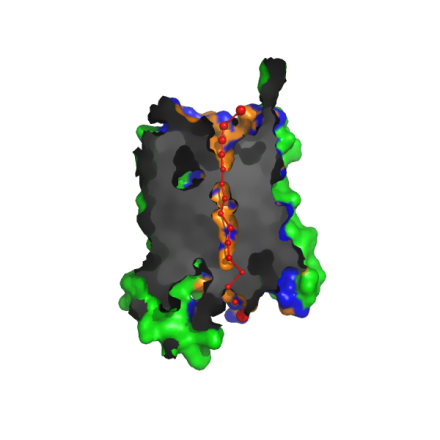 |
| --- | --- | --- |
| AipPIP1-1 | AipPIP1-2 | AipPIP1-3 |
| 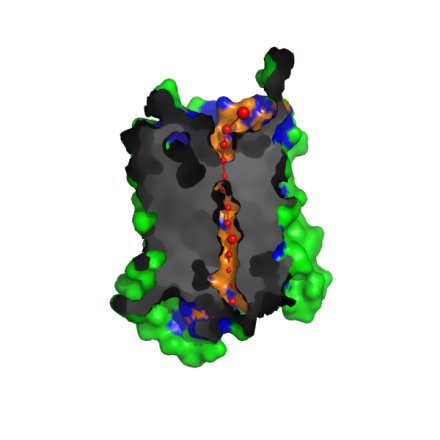 | 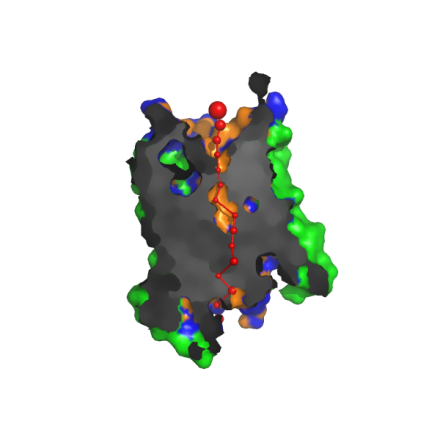 | 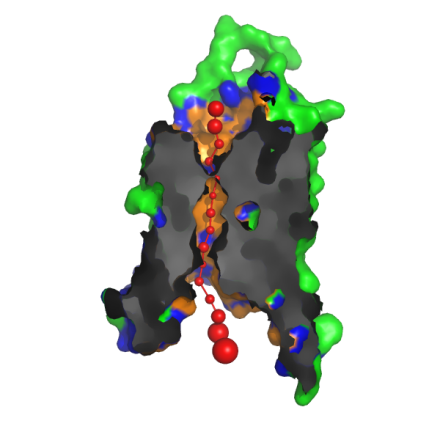 |
| AipPIP1-4 | AipPIP1-5 | AipPIP2-1 |
| 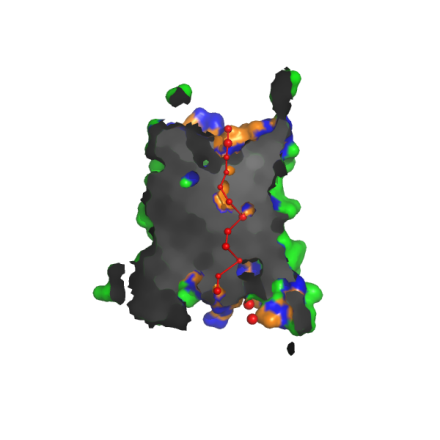 | 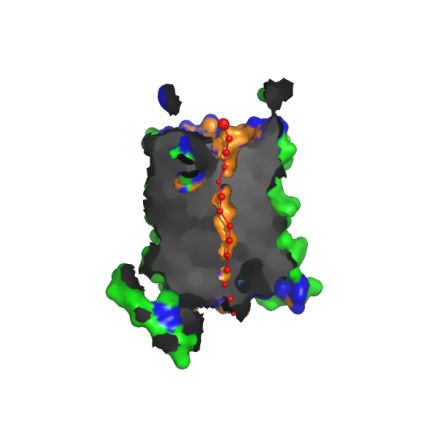 | 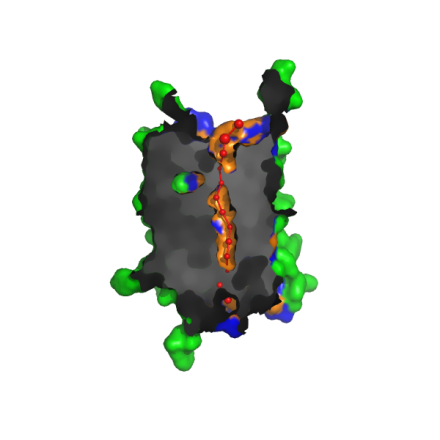 |
| AipPIP2-2 | AipPIP2-3 | AipPIP2-4 |

| 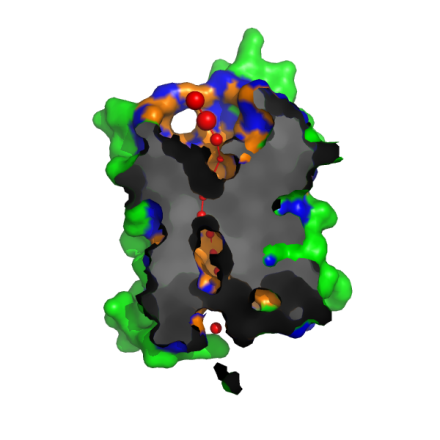 | 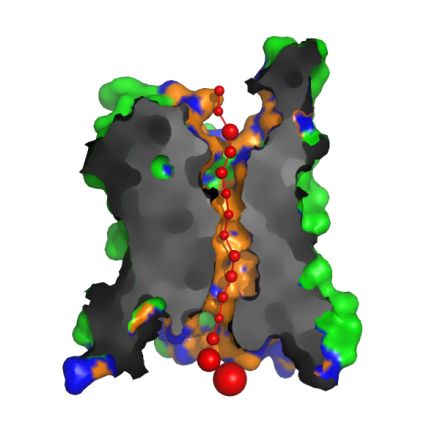 | 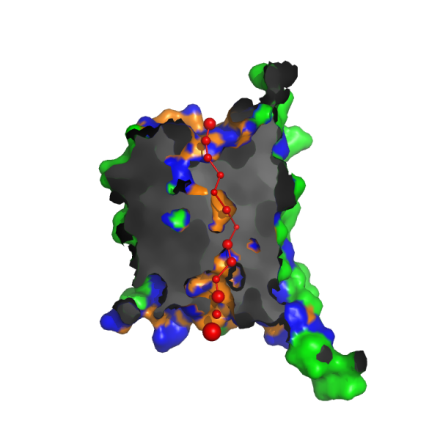 |
| --- | --- | --- |
| AipSIP1-1 | AipSIP1-2 | AipSIP2-1 |
| 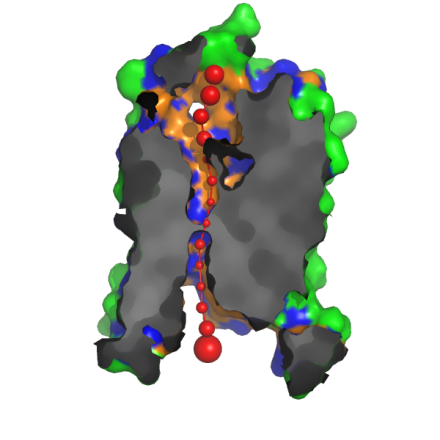 | 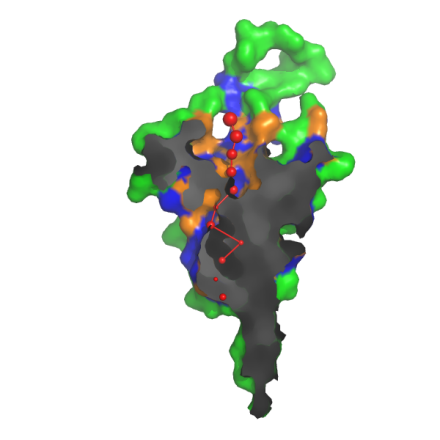 | 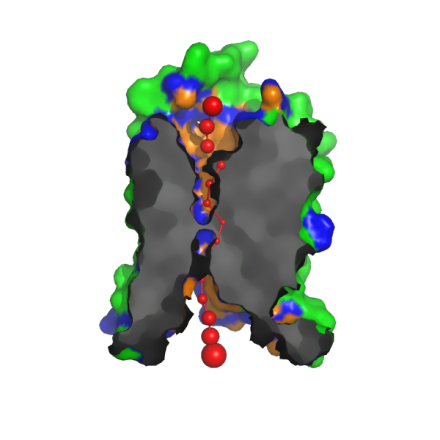 |
| AipTIP1-1 | AipTIP1-2 | AipTIP1-3 |
| 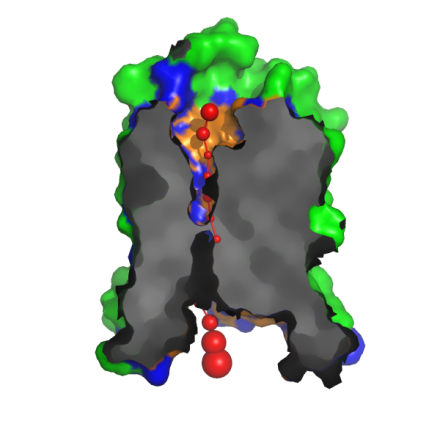 | 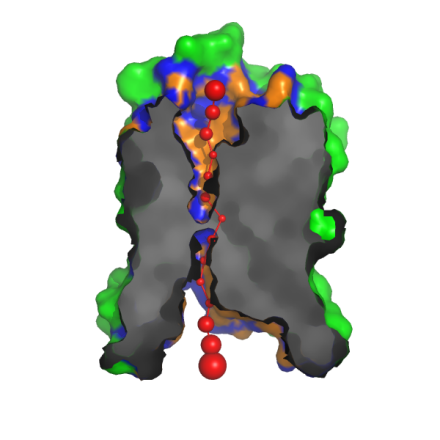 | 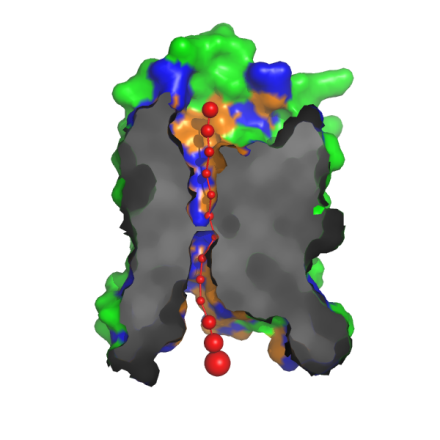 |
| AipTIP2-1 | AipTIP2-2 | AipTIP2-3 |

| 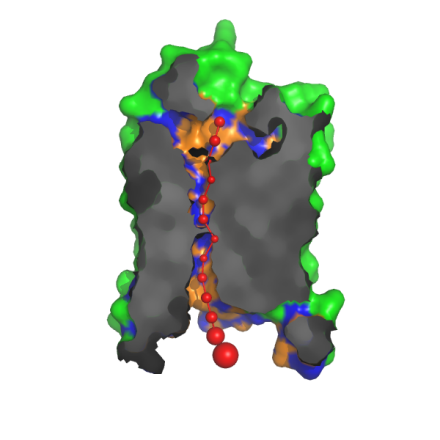 | 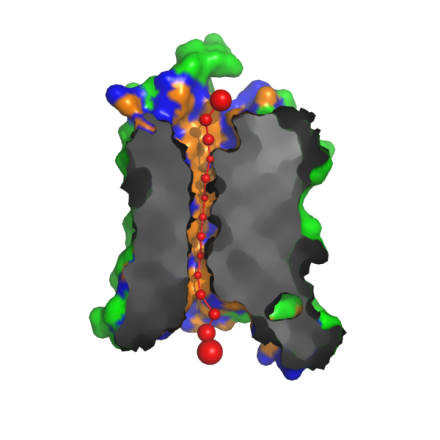 | 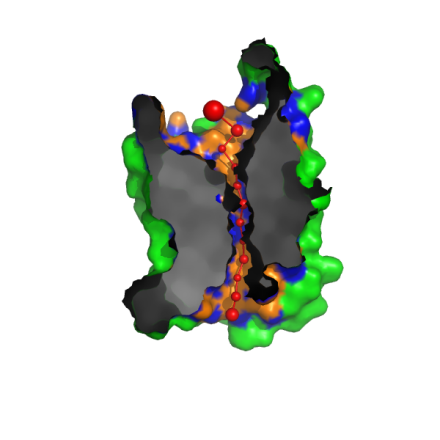 |
| --- | --- | --- |
| AipTIP3-1 | AipTIP4-1 | AipTIP4-2 |
| 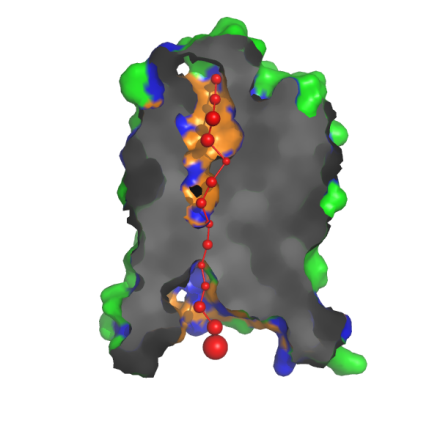 | 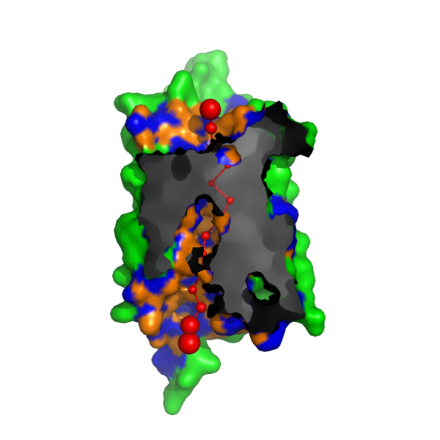 | 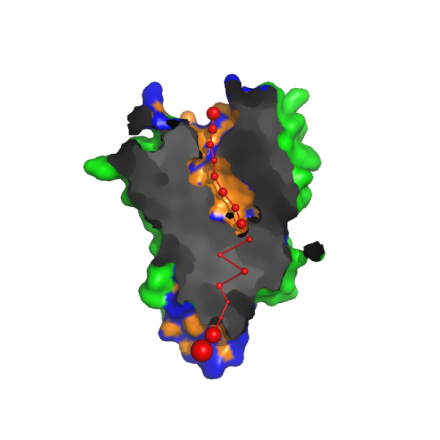 |
| AipTIP5-1 | AipXIP1-1 | AipXIP1-2 |
| 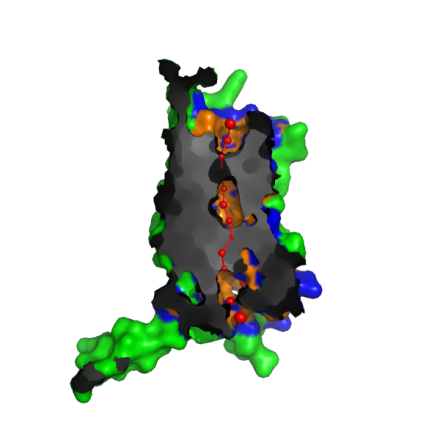 | 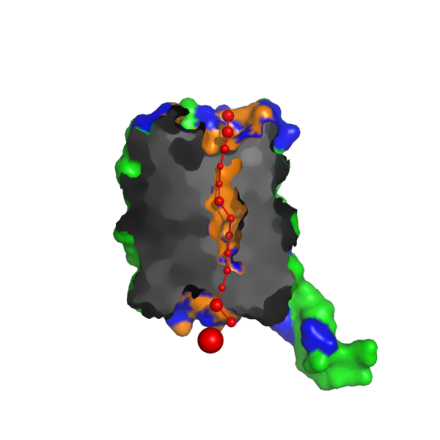 |  |
| AipXIP1-3 | AipXIP2-1 |  |
