# Supplementary material for: Identification and characterization of aquaporin genes in Arachis duranensis and Arachis ipaensis genomes, the diploid progenitors of peanut
Source: BMC Genomics. 2019 Mar 18;20:222. doi: 10.1186/s12864-019-5606-4 (PMC6423786; doi:10.1186/s12864-019-5606-4)
Supplement: Supplementary file 5 — Details of predicted sub-cellular location of Arachis duranensis aquaporins identified by using Wolfpsort server (DOCX 16 kb) [file 12864_2019_5606_MOESM5_ESM.docx]

**Additional file 5**

Details of predicted sub-cellular location of *Arachis duranensis* aquaporins identified by using Wolfpsort server

| **Gene_ID** | **Wolfpsort^1, 2^** |
| --- | --- |
| **AipPIP1-1** | plas: 7; cysk: 7 |
| **AipPIP1-2** | plas: 8; cyto: 4; cysk: 1.5; cysk_nucl: 1.5 |
| **AipPIP1-3** | plas: 7; cyto: 4; pero: 2 |
| **AipPIP1-4** | plas: 6; cysk: 3; mito: 2; cyto: 1; vacu: 1 |
| **AipPIP1-5** | plas: 8; cyto: 4; pero: 2 |
| **AipPIP2-1** | plas: 11; golg: 2 |
| **AipPIP2-2** | plas: 11; cyto: 1; pero: 1 |
| **AipPIP2-3** | plas: 12; E.R.: 1 |
| **AipPIP2-4** | plas: 10; golg: 2; cyto: 1 |
| **AipTIP1-1** | vacu: 6; plas: 4; chlo: 2; cyto: 2 |
| **AipTIP1-2** | vacu: 5; cyto: 3; chlo: 2; plas: 2; nucl: 1.5; cysk_nucl: 1.5 |
| **AipTIP1-3** | chlo: 6.5; cyto: 4; chlo_mito: 4; vacu: 2 |
| **AipTIP2-1** | vacu: 6; cyto: 4; nucl: 2.5; cysk_nucl: 2 |
| **AipTIP2-2** | plas: 5.5; cyto_plas: 4.5; vacu: 4; cyto: 2.5; extr: 1 |
| **AipTIP2-3** | vacu: 11; plas: 1; extr: 1 |
| **AipTIP3-1** | cyto: 3; vacu: 3; chlo: 2; mito: 2; pero: 2; plas: 1 |
| **AipTIP4-1** | vacu: 10; plas: 4 |
| **AipTIP4-2** | vacu: 6; cyto: 4; chlo: 2; nucl: 1 |
| **AipTIP5-1** | chlo: 10; mito: 2; plas: 1 |
| **AipNIP1-1** | plas: 6; vacu: 4; E.R.: 2; golg: 2 |
| **AipNIP1-2** | plas: 6; vacu: 3; E.R.: 2; nucl: 1; cyto: 1 |
| **AipNIP1-3** | vacu: 8; plas: 3; golg: 2 |
| **AipNIP1-4** | plas: 8.5; cyto_plas: 5; golg: 3; vacu: 1 |
| **AipNIP1-5** | vacu: 9; cyto: 3; plas: 2 |
| **AipNIP2-1** | plas: 7.5; E.R._plas: 4.5; cyto: 2; nucl: 1; mito: 1; vacu: 1 |
| **AipNIP3-1** | plas: 11; extr: 1; vacu: 1 |
| **AipNIP3-2** | plas: 6; E.R.: 3; cyto: 2; golg: 2 |
| **AipNIP3-3** | plas: 7; vacu: 4; cyto: 1; extr: 1 |
| **AipNIP4-1** | vacu: 5; cyto: 4; plas: 4 |
| **AipSIP1-1** | plas: 9; vacu: 3; cyto: 1 |
| **AipSIP1-2** | plas: 6; chlo: 4; golg: 2; vacu: 1 |
| **AipSIP2-1** | cyto: 6; vacu: 6; nucl: 1 |
| **AipXIP1-1** | nucl: 8; cyto: 4; vacu: 1 |
| **AipXIP1-2** | extr: 4; plas: 3; chlo: 2; cyto: 2; nucl: 1; vacu: 1 |
| **AipXIP1-3** | chlo: 4; cyto: 3; plas: 3; nucl: 2; vacu: 1 |
| **AipXIP2-1** | cyto: 7; nucl: 2; vacu: 2; chlo: 1; plas: 1 |

^1^https://www.genscript.com/wolf-psort.html?src=leftbar

^2^ Abbreviations of protein localization sites in the dataset are as follows: nucl: nucleus; chlo: chloroplast; cyto: cytosol; cysk: cytoskeleton; chlo_mito: chloroplast and mitochondria; plas: plastids; cyto_nucl: cytosol and nucleus; mito: mitochondria; pero: peroxisomes; and nucl_ plas: nucleus and plastids. The numbers mentioned against localization site indicate prior possible protein localization sites of the aquaporins.

Details of predicted sub-cellular location of *Arachis ipaensis* aquaporins identified by using Wolfpsort server

| **Gene_ID** | **Wolfpsort^1, 2^** |
| --- | --- |
| **AduPIP1-1** | plas: 8; cyto: 3; pero: 2 |
| **AduPIP1-2** | plas: 8; cyto: 3; pero: 2 |
| **AduPIP1-3** | extr: 4; chlo: 3; cyto: 3; vacu: 2; nucl: 1 |
| **AduPIP1-4** | mito: 7.5; chlo_mito: 6; chlo: 3.5; cyto: 1; plas: 1 |
| **AduPIP1-5** | plas: 9; cysk: 4 |
| **AduPIP2-1** | plas: 10; cyto: 2; pero: 1 |
| **AduPIP2-2** | plas: 12; golg: 2 |
| **AduPIP2-3** | plas: 10; golg: 2; cyto: 1 |
| **AduPIP2-4** | plas: 12; E.R.: 1 |
| **AduTIP1-1** | cyto: 10; vacu: 3 |
| **AduTIP1-2** | chlo: 7; plas: 3; mito: 2; cyto: 1 |
| **AduTIP1-3** | vacu: 11; plas: 2 |
| **AduTIP1-4** | cyto: 6; chlo: 4.5; vacu: 3; chlo_mito: 3 |
| **AduTIP2-1** | plas: 7.5; vacu: 5; cyto_plas: 4.5 |
| **AduTIP2-2** | vacu: 13 |
| **AduTIP2-3** | vacu: 6; plas: 3; nucl: 2.5; cyto: 2; cysk_nucl: 2 |
| **AduTIP3-1** | mito: 4; chlo: 3; cyto: 3; vacu: 3 |
| **AduTIP4-1** | vacu: 9; plas: 3; cyto: 1 |
| **AduTIP4-2** | vacu: 7; plas: 3; cyto: 2; chlo: 1 |
| **AduTIP5-1** | chlo: 11; mito: 2 |
| **AduNIP1-1** | vacu: 11; plas: 1; extr: 1 |
| **AduNIP1-2** | plas: 8; golg: 3; vacu: 2 |
| **AduNIP1-3** | plas: 5; vacu: 4; cyto: 2; E.R.: 2 |
| **AduNIP1-4** | plas: 6.5; cyto_plas: 4; vacu: 3; golg: 3 |
| **AduNIP1-5** | plas: 9; golg: 3; vacu: 1 |
| **AduNIP2-1** | plas: 8; vacu: 3; golg: 3 |
| **AduNIP3-1** | plas: 11; extr: 1; vacu: 1 |
| **AduNIP3-2** | plas: 11; E.R.: 2 |
| **AduSIP1-1** | plas: 8; vacu: 4; cyto: 1 |
| **AduSIP1-2** | chlo: 5; plas: 4; extr: 2; vacu: 2 |
| **AduSIP2-1** | cyto: 6; vacu: 3; E.R.: 3; mito: 1 |
| **AduXIP2-1** | cyto: 6; plas: 4; E.R.: 3 |

^1^https://www.genscript.com/wolf-psort.html?src=leftbar

^2^ Abbreviations of protein localization sites in the dataset are as follows: nucl: nucleus; chlo: chloroplast; cyto: cytosol; cysk: cytoskeleton; chlo_mito: chloroplast and mitochondria; plas: plastids; cyto_nucl: cytosol and nucleus; mito: mitochondria; pero: peroxisomes; and nucl_ plas: nucleus and plastids. The numbers mentioned against localization site indicate prior possible protein localization sites of the aquaporins.
