# Supplementary material for: Identification and characterization of aquaporin genes in Arachis duranensis and Arachis ipaensis genomes, the diploid progenitors of peanut
Source: BMC Genomics. 2019 Mar 18;20:222. doi: 10.1186/s12864-019-5606-4 (PMC6423786; doi:10.1186/s12864-019-5606-4)
Supplement: Supplementary file 6 — Amino acid sequences of aquaporins from Arabidopsis thaliana, Glycine max and Oryza sativa used for BLASTp search. Sequence names of A. thaliana, G. max and O. sativa are preceded by the prefixes At, Gm and Os respectively. (DOCX 27 kb) [file 12864_2019_5606_MOESM6_ESM.docx]

**Additional file 6**

Amino acid sequences of aquaporins from *Arabidopsis thaliana*, *Glycine max* and *Oryza sativa* used for BLASTp search. Sequence names of *A. thaliana*, *G. max* and *O. sativa* are preceded by the prefixes At, Gm and Os respectively.

>OsNIP1-1

MAGGDNNSQTTNGGSGHEQRAMEEGRKQEEFAADGQGCGLAFSVPFIQKIIAEIFGTYFLIFAGCGAVTINQSKNGQITFPGVAIVWGLAVMVMVYAVGHISGAHFNPAVTLAFATCRRFPWRQVPAYAAAQMLGATLAAGTLRLMFGGRHEHFPGTLPAGSDVQSLVLEFIITFYLMFVISGVATDNRAIGELAGLAVGATILLNVLIAGPISGASMNPARSLGPAMIGGEYRSIWVYIVGPVAGAVAGAWAYNIIRFTNKPLREITKSGSFLKSMNRMNSST

>OsNIP1-2

MAVVVDGVSPPWSKEAVVHLLSEFSSPDHVSISVTAHQPCLLKNGLEETETTRASGLPNLRNDSINRVLIESLVERLIALPLSQGERWLGERMEQQQEPWKKGKTARRSNFQRTLIADTDPWCMCMNKNLLILAEILGTYFMIFAGCGAVVVNQSTGGAVTFPGICAVWGLVVMVLVYTVSHISGAHFNPAVTVAFATCGRFRWKQVPSYVVAQVLGSTMASLTLRVVFGGGGGGARGEHLFFGTTPAGSMAQAAALEFVISFFLMFVVSGVATDNRAIGELAGLAVGATVAVNVLFAGPVTGASMNPARSLGPAMVAGRYGGVWVYVAAPVSGTVCGAWAYNLLRFTDKPLRDIANTASFLRRSSRRS

>OsNIP1-3

MAGGEHGVNGQHEETRAMEEGSRDHQARCENSEQDGGSKSSSNNHPMFSVQFAQKVIAEILGTFFLIFAGCAAVAVNKRTGGTVTFPGICITWGLAVMVMVYSVGHISGAHLNPAVTLAFATCGRFPWRRVPAYAAAQVAGSAAASAALRALFGGAPEHFFGTAPAGSDVQSLAMEFIITFYLMFVVSGVATDNRAIGELAGLAVGATVLVNVLFAGPISGASMNPARTIGPAIILGRYTGIWVYIAGPVFGAVAGAWAYNLIRFTDKPLREITMTASFIRSTRRN

>OsNIP1-4

MARREVDDSYTNGSVVEVVSIEEGSKMDKEDDHQNPQAPDGGDVVVCGMPMSFTFLQMLLAEFLATFFLMFAGLGAITVEEKKGAVTFPGVAVAWGAAVMAMVYAVGHVSGAHLNPAVTLGFAVAGRFPWRRAPAYALAQTAAATAASVVLRLMFGGRHAPVPATLPGGAHAQSLVIEFVITFYLMFVIMAVATDDQAVGHMAGVAVGGTIMLNVLFAGPVSGASMNPARSIGPALVGSKYTALWVYILGPFAGAAAGAWAYSLIRLTGDRTD

>OsNIP2-1

MASNNSRTNSRANYSNEIHDLSTVQNGTMPTMYYGEKAIADFFPPHLLKKVVSEVVATFLLVFMTCGAAGISGSDLSRISQLGQSIAGGLIVTVMIYAVGHISGAHMNPAVTLAFAVFRHFPWIQVPFYWAAQFTGAICASFVLKAVIHPVDVIGTTTPVGPHWHSLVVEVIVTFNMMFVTLAVATDTRAVGELAGLAVGSAVCITSIFAGAISGGSMNPARTLGPALASNKFDGLWIYFLGPVMGTLSGAWTYTFIRFEDTPKEGSSQKLSSFKLRRLRSQQSIAADDVDEMENIQV

>OsNIP2-2

MASTTAPSRTNSRVNYSNEIHDLSTVQSVSAVPSVYYPEKSFADIFPPNLLKKVISEVVATFLLVFVTCGAASIYGEDMKRISQLGQSVVGGLIVTVMIYATGHISGAHMNPAVTLSFAFFRHFPWIQVPFYWAAQFTGAMCAAFVLRAVLYPIEVLGTTTPTGPHWHALVIEIVVTFNMMFVTCAVATDSRAVGELAGLAVGSAVCITSIFAGPVSGGSMNPARTLAPAVASNVYTGLWIYFLGPVVGTLSGAWVYTYIRFEEAPAAAGGAAPQKLSSFKLRRLQSQSMAADEFDNV

>OsNIP3-1

MEMAAPNGGGAAGMSSPVNGASAPATPGTPAPLFAGPRVDSLSYERKSMPRCKCLPAAVAEAWAPSAHGCVVEIPAPDVSLTRKLGAEFVGTFILIFFATAAPIVNQKYGGAISPFGNAACAGLAVTTIILSTGHISGAHLNPSLTIAFAALRHFPWLQVPAYVAVQVLGSICAGFALKGVFHPFLSGGVTVPDPTISTAQAFFTEFIITFNLLFVVTAVATDTRAVGELAGIAVGAAVTLNILIAGPTTGGSMNPVRTLGPAVAAGNYRQLWIYLIAPTLGAVAGAGVYTAVKLRDENGETPRPQRSFRR

>OsNIP3-3

MAENMVMVSSSDENHNQVAIDLCSASPVDRSLSAAAGGSTTPRSPGFSMVVVPVESPEKTTGKPQTDDHDQQQGRAKEVPLVKKAAAEFIGTFILVFTVLSTVVMDARHGGAETLVGVAASAGLAVVAVVLSVVHISGSHLNPAVSLAMAALGHLPPAHLLPYAAVQTAASLAAAFLAKGVYRPARPAVMATVPAAGVGAGEAFVVESKELVAIAIAAAIMMNALVGGPSTGPSMNPARTIGAAVATGEYRQMWIYLVAPPLGAIAGAATYTLIKP

>OsNIP3-2a

MEGGKMSSMGMDAASASVTVPPMQMQAGDQSNRIAIIISPRAGSSKILPFELVNGAANAGSQRHADPAESTPEAHHHLWHPVDLPKIKPPVPLVKKVGAEFFGTFTLIFTVLSTIIMDEQHKGVESLLGIATSAGLAVTVLVLSLIHISGCHLNPAVSIAMTVFGHLPPAHLLPYIAAQILGSITASFAVKGMYHPVNPGIVTVPKVGTVEAFFLEFVTTFVLLFIITALATDPNAVKELIAVAVGATIMMNALVAGPSTGASMNPARTLGPAIATGRYTQIWVYLVATPLGAVAGEGFYFAIKL

>OsNIP3-2b

MKNKKPTNSEKKKIQLAITVHIGIGYYTCVCMSGSKTHSASKKMDEGSSPASTSATAAAAAANLESTSFDDGRSHSSKITPIELVVVNPEEPPPASRSRGHGPRRRSWRRRPCPPLAKKAAAEFVGTFILIFAMLSTIVTDAQRGGVEGLVGVAASIGLAVAVLVMSLAHVSGAHINPAVSVAMAAFGRLQPAHLLPYAAAQVLGAVAAAAAVDGIFHPASRGWMVSVPKVGTVEAFFLEFVTTFVLLFVITAVSADPNAVKELIAVAVGGTAMMNVLVAGPSTGASMNPARTLGTAIVAGNYTQIWVYMVSTPLGAIAGTGAYFAIKL

>OsNIP4-1

MTTDHAGKKVDVVVVGNVDGEHVGVEQARHDLHEEAAAAAAADHHATRGLAIGFLIREVMVEGLASFLVVFWSCVAALMQEMYGTLTFPMVCLVVAMTVAFVLSWLGPAHFNPAVTITFAAYRRFPVWPKLPLYVAAQLAGSLLACLSVNAVMRPRHDHFYGTAPVVVHGTRLPFLMEFLASAVLMIVIATVATDGTAGKTVGGIAIGAAVGGLGLVIGPVSGGSMNPARTLGPAIVLGRYDGVWIYVVAPVAGMLVGALCNRAVRLSHRIVAFLCGTSVGIAGSP

>OsPIP1-1

MEGKEEDVRLGANRYSERQPIGTAAQGAGDDKDYKEPPPAPLFEPGELKSWSFYRAGIAEFVATFLFLYITILTVMGVSKSSSKCATVGIQGIAWSFGGMIFALVYCTAGISGGHINPAVTFGLFLARKLSLTRAIFYIVMQCLGAICGAGVVKGFQQGLYMGNGGGANVVASGYTKGDGLGAEIVGTFILVYTVFSATDAKRNARDSHVPILAPLPIGFAVFLVHLATIPITGTGINPARSLGAAIIYNKDHAWNDHWIFWVGPFVGAALAAIYHQVIIRAIPFKSRS

>OsPIP1-2

MEGKEEDVRLGANKFSERQPIGTAAQGSDDKDYKEPPPAPLFEPGELKSWSFYRAGIAEFMATFLFLYITVLTVMGVNNSTSKCATVGIQGIAWSFGGMIFALVYCTAGISGGHINPAVTFGLFLARKLSLTRALFYMVMQCLGAICGAGVVKGFQKGLYETTGGGANVVAPGYTKGDGLGAEIVGTFILVYTVFSATDAKRNARDSHVPILAPLPIGFAVFLVHLATIPITGTGINPARSLGAAIIYNRGHAWDDHWIFWVGPFIGAALAAIYHQVVIRAIPFKSRS

>OsPIP1-3

MEGKEEDVRLGANRYTERQPIGTAAQGAEEKDYREPPAAPVFEVEELTSWSFYRAGIAEFVATFLFLYISILTVMGVNKSASKCATVGIQGIAWSFGGMIFALVYCTAGISGGHINPAVTFGLFLARKLSLTRAVFYMAMQCLGAICGAGVVKGFQRGLYMGSGGGANAVNPGYTKGDGLGAEIVGTFVLVYTVFSATDAKRNARDSHVPILAPLPIGFAVFLVHLATIPITGTGINPARSLGAAIVYNRAHAWHDHWIFWVGPFIGAALAAIYHVVVIRAIPFKSRD

>OsPIP2-1

MGKDEVMESGGAAGEFAAKDYTDPPPAPLIDAAELGSWSLYRAVIAEFIATLLFLYITVATVIGYKHQTDASASGADAACGGVGVLGIAWAFGGMIFILVYCTAGISGGHINPAVTFGLFLARKVSLVRAILYIVAQCLGAICGVGLVKAFQSAYFNRYGGGANTLAAGYSKGTGLAAEIIGTFVLVYTVFSATDPKRNARDSHVPVLAPLPIGFAVFMVHLATIPITGTGINPARSIGAAVIFNNEKAWHNHWIFWVGPFVGAAIAAFYHQYILRAGAIKALGSFRSNA

>OsPIP2-2

MAKDIEASAPEGGEFSAKDYTDPPPAPLIDVEELTKWSLYRAVIAEFIATLLFLYITVATVIGYKHQSDATVNTTDAACSGVGILGIAWAFGGMIFILVYCTAGISGGHINPAVTFGLFLARKVSLIRAVLYIIAQCLGAICGVGLVKGFQSSYYARYGGGANELSDGYSKGTGLGAEIIGTFVLVYTVFSATDPKRNARDSHIPVLAPLPIGFAVFMVHLATIPITGTGINPARSLGTAVIYNKDKAWDDQWIFWVGPLIGAAIAAAYHQYVLRASAAKLGSYRSNA

>OsPIP2-3

MAKDIEAAAAAEGGEYMAKDYSDPPPAPLIDAEELTKWSLYRAVIAEFVATLLFLYITVATVIGYKHQSDPGANAADAACSGVGILGIAWAFGGMIFILVYCTAGVSGGHINPAVTFGLFLARKVSLVRAVLYIVAQSLGAICGVGLVKGFQSAFYVRYGGGANELSDGYSKGTGLAAEIIGTFVLVYTVFSATDPKRNARDSHVPVLAPLPIGFAVFMVHLATIPITGTGINPARSLGAAVIYNQHKAWHDHWIFWVGPLIGAAIAAAYHQYVLRASAAKLGSSSSFRG

>OsPIP2-4

MGKEVDVSTLEAGGARDYIDPPPAPLVDVDELGKWSLYRALIAEFVATLLFLYVTVATVIGYKHQTDAAVNGADAACGGVGVLGIAWAFGGMIFILVYCTAGVSGGHINPAVTLGLFLARKVSLVRALLYMAAQCLGAICGVALVKGFQSSLYDRYGGGANELAAGYSTGTGLAAEIIGTFVLVYTVFSATDPKRNARDSHVPVLAPLPIGFAVFMVHLATIPITGTGINPARSLGVAVVYNNNKAWSDQWIFWVGPFIGAAIAALYHQVILRASARGYGSFRSNA

>OsPIP2-5

MGKEADVEAGGVRDYEDPPPAPLVDIDELGRWSLYRAVIAEFVATLLFLYVTVATVIGYKHQTDASASGDDAACGGVGVLGIAWAFGGMIFILVYCTAGISGGHINPAVTFGLFLARKVSLVRAILYIVAQCLGAVCGVALVKGFQSSFYDRYGGGANELAAGYSKGTGLAAEIIGTFVLVYTVFSATDPKRNARDSHVPVLAPLPIGFAVFMVHLATIPVTGTGINPARSLGAAVVYNNSKAWSDQWIFWVGPFIGAAIAALYHQIVLRASARGYGSFRSNA

>OsPIP2-6

MSKEVSEEPEHVRPKDYTDPPPAPLFDVGELRLWSFYRALIAEFIATLLFLYITVATVIGYKVQSSADQCGGVGTLGIAWAFGGMIFILVYCTAGISGGHINPAVTFGLLLARKVSVIRAVMYIVAQCLGGIVGVGIVKGIMKHQYNANGGGANMVASGYSTGTALGAEIIGTFVLVYTVFSATDPKRNARDSHVPVLAPLPIGFAVFMVHLATIPITGTGINPARSIGAAVIYNQKKAWDDHVSCSPLTATLCSCVHFTKCLCLHMLNCSGSSGRGRSSERWRRRRITSTSSGRQPSRRWAPSGATPATELRWPWLLCCCWPGWTWSYRLHVRECV

>OsPIP2-7

MASKEEVAVETVEGGAAAAKAPYWDPPPAPLLDTSELGKWSLYRALIAEFMATLIFLYVSIATVIGYKNQRATVDACTGVGYLGVAWSFGATIFVLVYCTGGVSGGHINPAVTLGLFFGRKLSLVRTVLYVVAQCLGAIAGAGIVKGIMKRPYDALGGGANTVSDGYSAAGALGAEIVGTFILVYTVFSATDPKRTARDSFIPVLVPLPIGFAVFVVHLATIPITGTGINPARSLGAAVLYNQHAAWKDHWIFWVGPVIGAFLAAAYHKLVLRGEAAKALSSFRSTSVTA

>OsPIP2-8

MAAGSGSGSNPKDYQDPPPAPLVDTGELGKWSLYRAAIAEFTATLLLVCISVSTVIGEKRQSGEGGAGVLGIAWAFGGLIFVLVYCTAGISGGHMNPAVTFAMVLARRVSLPRAALYTMAQCVGAVCGAGLARAMHGGGQYARHGGGANELAAGYSAGAGVVAEMVGTFVLVYTVFSATDPKRKARDSHVPVLAPLPIGLAVLVVHLATIPITGTGINPARSLGPALVLGLGTTKAWSHLWIFWVGPFAGAAAAMIYHHYILRGAAAKAFASSSYRSPHF

>OsSIP1-1

MAVAAVRAAAADAAVTFLWVLCVSTLGASTAAVTSYLRIHEGIHYALLVTVSLLSVLLFAFNLLCDALGGASFNPTALAAFHAAGLSSPRHSSLFPLALRFPAQAAGAVGGAMAISELMPEQYKHMLGGPSLKVDLHTGAAAELVLTFVITLAVLWIIVKGPRNPIVKTWMLSISTVCLVLTGAAYTGPSMNPANAFGWAYVNNRHNTWEQFYVYWICPFVGAVLAAWVFRAVFPPPAPKPKAKKA

>OsSIP2-1

MSPAPPPSRGRIRPWLVVGDLVVAAMWVCAGALVKLAVYGVLGLGGRPEADAVKVALSLVYMFFFAWLEGFTGGASYNPLTVLAGALASRAGPSLYLFAAFVRMPAQVFGSILGVKLIRAALPKVGKGAPLSVGVHHGALAEGLATFMVVIVSVTLKKKEMKGFFMKTWISSIWKMTFHLLSSDITGGVMNPASAFAWAYARGDHTTFDHLLVYWLAPLQATLLGVWVVTLLTKPKKIEEEADESKTKKE

>OsTIP1-1

MPIRNIAVGSHQEVYHPGALKAALAEFISTLIFVFAGQGSGMAFSKLTGGGATTPAGLIAAAVAHAFALFVAVSVGANISGGHVNPAVTFGAFVGGNITLFRGLLYWIAQLLGSTVACFLLRFSTGGLATGTFGLTGVSVWEALVLEIVMTFGLVYTVYATAVDPKKGSLGTIAPIAIGFIVGANILVGGAFDGASMNPAVSFGPALVSWSWESQWVYWVGPLIGGGLAGVIYEVLFISHTHEQLPTTDY

>OsTIP1-2

MPVSRIAVGAPGELSHPDTAKAAVAEFISMLIFVFAGSGSGMAFSKLTDGGGTTPSGLIAASLAHALALFVAVAVGANISGGHVNPAVTFGAFVGGNISLVKAVVYWVAQLLGSVVACLLLKIATGGAAVGAFSLSAGVGAWNAVVFEIVMTFGLVYTVYATAVDPKKGDLGVIAPIAIGFIVGANILAGGAFDGASMNPAVSFGPAVVTGVWDNHWVYWLGPFVGAAIAALIYDIIFIGQRPHDQLPTADY

>OsTIP2-1

MVKLAFGSLGDSFSATSVKAYVAEFIATLLFVFAGVGSAIAYGQLTNGGALDPAGLVAIAIAHALALFVGVSVAANISGGHLNPAVTFGLAVGGHITILTGLFYWIAQLLGASIACLLLKFVTHGKAIPTHGVAGISELEGVVMEIVITFALVYTVYATAADPKKGSLGTIAPIAIGFIVGANILAAGPFSGGSMNPARSFGPAVAAGNFAGNWVYWVGPLIGGGLAGLVYGDVFIGSYQPVADQDYA

>OsTIP2-2

MSGNIAFGRFDDSFSAASLKAYVAEFISTLVFVFAGVGSAIAYTKLTGGAPLDPAGLVAVAVCHGFGLFVAVAIGANISGGHVNPAVTFGLALGGQITILTGVFYWIAQLLGAIVGAVLVQFCTGVATPTHGLSGVGAFEGVVMEIIVTFGLVYTVYATAADPKKGSLGTIAPIAIGFIVGANILVAGPFSGGSMNPARSFGPAVASGDYTNIWIYWVGPLVGGGLAGLVYRYVYMCGDHAPVASSEF

>OsTIP3-1

MSTAAARPGRRFTVGRSEDATHPDTIRAAISEFLATAIFVFAAEGSILSLGKLYQDMSTPGGLVAVSLAHALALAVAVAVAVNISGGHVNPAITFGALLGGRLSLIRALFYWLAQLLGAVVATLLLRLTTGGMRPPGFALASGVGDWHAVLLEATMTFGLMYAYYATVIDPKRGHVGTIAPLAVGFLLGANMLAGGPFDGAGMNPARVFGPALVGWRWRHHWVYWLGPFVGAGLAGLLYEYLVIPSADAAPHGGAHQPLAPEDY

>OsTIP3-2

MLPGRHTPRRADAAAAAAAMEPLVPGATRAALSEFVATAVFVFAAEGSVYGLWKMYRDTGTLGGLLVVAVAHALALAAAVAVSRNASGGHVNPAVTFGVLVGRRISFARAALYWAAQLLGAVLAVLLLRLASGGMRPMGFTLGHRIHERHALLLEVVMTFGLVYTVYATAVDRRSGGGDIAPLAIGLVAGANILAGGPFDGAAMNPARAFGPALVGWNWRHHWVYWLGPLIGAGMAGALYEFVMAEQPEPPAAADTRLPVAAEDY

>OsTIP4-1

MAKEVDPCDHGEVVDAGCVRAVLAELVLTFVFVFTGVAATMAAGVPEVAGAAMPMAALAGVAIATALAAGVLVTAGFHVSGGHLNPAVTVALLARGHITAFRSALYVAAQLLASSLACILLRYLTGGMATPVHTLGSGIGPMQGLVMEIILTFSLLFVVYATILDPRSSVPGFGPLLTGLIVGANTIAGGNFSGASMNPARSFGPALATGVWTHHWIYWLGPLIGGPLAGLVYESLFLVKRTHEPLLDNSF

>OsTIP4-2

MPLLPMTKLELGHRGEAWEPGCLRAVAGELLFTFLFVFIGVASTITAGKAAGGAGEAAAVTAAAMAQALVVAVLATAGFHVSGGHLNPAVTLSLAVGGHITLFRSALYVAAQLAGSSLACLLLRCLTGGAATPVHALADGVGPVQGVAAEAVFTFTLLLVICATILDPRRAAPPGTGPLLTGLLVGANTVAGGALTGASMNPARSFGPALATGEWAHHWVYWVGPLAGGPLAVVAYELLFMDVEDAGGAHQPLPQE

>OsTIP4-3

MAKLALGHHREATDPGCLRAVVAELLLTFLFVFSGVGSAMAAAKLGGGGDTIMGLTAVAAAHALVVAVMVSAGLHVSGGHINPAVTLGLAAGGHITLFRSALYAAAQLLGSSLACLLLAALTGGEEAVPVHAPAPGVGAARAVAMEAVLTFSLLFAVYATVVDRRRAVGALGPLLVGLVVGANILAGGPYSGASMNPARSFGPALAAGEWADHWIYWVGPLIGGPLAGLVYEGLFMGPPGHEPLPRNDGDF

>OsTIP5-1

MANICANMKRCFSPPALRAYFAEFFSTFLFVFIAVGSTISARMLTPDETSDASSLMATAVAQAFGLFAAVFIAADVSGGHVNPAVTFAYAIGGHITVPSAIFYWASQMLGSTFACLVLHYISAGQAVPTTRIAVEMTGFGAGILEGVLTFMVVYTVHVAGDPRGGGFGGRKGPAATALGALVVGAVTGACVLAAGSLTGASMNPARSFGPAVVSGHYSNQAVYWAGPMVGAAVAALVHQALVFPTVPEPAPAPATNESARHGSVQTVVV

>GmNIP1-1

MEENGGNIHADSTFCGSPAVVQVIQKVIAELIGTYFLIFAGCCSVIINNAEETKGRITFPGICLVWGFSVTILVYSLAHVSGAHFNPAVTLSFAIYRHFPLRLAYIKSTVPLYFIAQVLGSFLASGTLYLLFEVNEKTYFGTIPSGSYIQSLVFEILTSFLLMFVVCAVSTDNRAIGKLGGIAVGMTIIVNVFIAGPISGASMNPARSLGPALVMWVYNGIWIYVVGPFVGAILGATCYNLIRYTDKPLREIGASSKIFKTSACTSAT

>GmNIP1-2

MAEVIGTYFVVFAGCGSVAVNKIYGSVTFPGVCVTWGLIVMVMIYSLRRISGAHFNPAVTITLAIFRRFSYKEVPLYIFAQLLGSILASGTLALMLDVTPKAYFGTVPVGSNGQSLVAEIIITFLLMFVISAVSTDDRAVGDFAGVAVGMTIMLNVFIAGPVSGASMNPARSIGPALIKHVYKGLWVYVVGPVVGSIAGALAYYFLRSIDKSSSE

>GmNIP1-3

MAAKSEGIQEEMPSMEEGVSSPSPSRTCNVSHNCCSNHVVALAQKVFAEVIGTYFVVFAGCGSVAVNKIYGSVTFPGVCVTWGLIVMVMIYSLRHISGAHFNPAVTITLAIFRRFSYKQVPLYIFAQLLGSILASGTLALMLDVTPKAYFGTVPVGSNGQSLVAEVIITFLLMFVISAVSTDDKAVGDFAGVAVGMTIMLNVFIAGPVSGASMNPARSIGPALIKHVYQGLWIYVVGPIVGSIAGALAYNFLRSPYKPPSE

>GmNIP1-4

MEPSDSFVSVPFLQKLVAEVVGTYFLIFAGCASVVVNKNNDNVVTLPGIAIAWGLVVTVLVYTVGHISGAHFNPAVTIAFASTRRFPLMQVPAYVAAQLLGSTLASGTLKLLFMGKHDQFSGTLPNGTNLQAFVFEFIITFLLMFVISGVATDNRAVTSLTLLPLLKFVHTSWPVTGASMNPVRSLGPAIVHGEYRGIWIYLLAPVVGAIAGALVYNTIRYTDKPLREITKSASFLKGRGGST

>GmNIP1-5

MADYSAGTESQEVVVNVTKNTSETIQRSDSLVSVPFLQKLVAEAVGTYFLIFAGCASLVVNENYYNMITFPGIAIVWGLVLTVLVYTVGHISGGHFNPAVTIAFASTRRFPLIQVPAYVVAQLLGSILASGTLRLLFMGNHDQFSGTVPNGTNLQAFVFEFIMTFFLMFVICGVATDNRAVGELAGIAIGSTLLLNVIIGGPVTGASMNPARSLGPAFVHGEYEGIWIYLLAPVVGAIAGAWVYNIVRYTDKPLSEITKSASFLKGRAASK

>GmNIP1-6

MDENSATNGTHEVVLDVNRDVSRTTQASRSCVNVSFLQKLVAEVVGTYFLIFAGSASVVVNKNNNNVVTLPGISIVWGLVVMVLVYSVGHISGAHFNPAVTIAFASTKRFPLKQVPVYVVAQVVGSTLASGTLRLLFSGKEAQFSGTLPSGSNLQAFVIEFLITFFLMFVVSGVATDNRAIGELAGIAVGSTVLLNVMFAGPITGASMNPARSIGPAIVHKEYRGIWIYLVSPTLGAVAGAWVYNSIRYTDKPLREITKSASFLKGVASR

>GmNIP1-7

MDENSATNGTHEVILDVNKDVSRTTQPSRSCVNVSFLQKLVAEVVGTYFLIFAGCASVVVNKNNNNVVTHPGISIVWGLVVMVLVYSVGHISGAHFNPAVTIAFASTRRFPLKQVPVYVVAQVVGSTLASATLRLLFSGKETQFSGTLPSGSNLQAFVIEFLITFFLMFVISGVATDDRAIGELAGIAVGSTVLLNVMFAGPITGASMNPARSIGPAILHNEYRGIWIYIVSPTLGAVAGTWVYNTIRYTDKPLREITKSTSFLKGVGRSGSSR

>GmNIP1-8

MSVVADNSANNGSHQVVLNVNGDAPKKCDDSANQDCVPLLQKLVAEVVGTYFLIFAGCASVVVNLDKDKVVTQPGISIVWGLTVMVLVYSVGHISGAHFNPAVTIAHATTKRFPLKQVPAYVIAQVVGATLASGTLRLIFNGKNDHFAGTLPSGSDLQSFVVEFIITFYLMFVISGVATDNRAIGELAGLAVGSTVLLNVMFAGPITGASMNPARSLGPAIVHHEYRGIWIYLVSPTLGAVAGTWAYNFIRYTNKPVREITKSASFLKGSEAE

>GmNIP1-9

MYTNNGSHQVVLNVNGDASKKCDDSSNQDCVPLLQKLVAEVVGTYFLIFAGCASVVVNLDKDKVVTQPGISIVWGLTVMVLVYSVGHISGAHFNPAVTIAHATTKRFPLKQVPAYVIAQVVGATLASGTLRLIFNGKSDHFTGTLPGGSDLQSFVVEFIITFYLMFVISGVATDNRAIGELAGLAVGSTVLLNVMFAGPITGASMNPARSLGPAIVHNEYKGIWIYLVSPTLGAVAGTWAYNFIRYTNKPVREITKSASFLKGGEAE

>GmNIP2-1

MEGTSSQSTFAFIPSTIETPSPSIPEISSSSSPGSLARIAQSYPPGFPRKVLAEIIGTFLLVFVGSGSAGLSKIDERMVSKLGASLAGGLIVTVMIYSIGHISGAHMNPAVSLAFTAVRHLPWPQLPFYIAAQLTGAISASYTLRELLRPSNEIGGTSPAGSHIQALIMEMVTTYTMVFISMAVATDSNATGQLSGVAVGSSVCIASIVAGPISGGSMNPARTLGPAIATSYYKGLWVYFVGPITGAVLAAWSYNVIRDTEHPGFPISLSSISSKVRQSIGGTEQKSDQRCLV

>GmNIP2-2

MEGTTSQSTFTFIPSTIETPSPSIPEISSSSPSPGGSLARVAQSYPPGFPRKVFAEVIGTFLLVFVGSGSAGLSKIDESMVSKLGASLAGGLIVTVMIYSIGHISGAHMNPAVSLAFTAVRHLPWPQLPFYVAAQLTGAISASYTLRELLRPSDEIGGTSPAGSHIQALIMEMVSTYTMVFISMAVATDSNATGQLSGVAVGSSVCIASIVAGPISGGSMNPARTLGPAIATSYYKGLWVYFVGPITGAVLAAWSYNVIRDTEHPGFPISLSSISSKVRQSIGGTEQKSDQRCLV

>GmNIP3-1

MDNNEEIPSTPATPGTPGAPLFGGFSNGRNNNSKKSLLKSCRCFSVEEWSLEDGGLPAVSCSLPLPSPPPVVPLARKIGAEFIGTFILMFAGTAAAIVNQKTNGSETLIGCAATTGLAVMIVILATGHISGAHLNPAVTISFAALKHFPWKHVPMYIGAQVLASICAGFALKGVYHPFMSGGVTVPSGGYGQSFALEFIIGFNLMFVVTAVATDTRAVGELAGIAVGATVMLNILIAGPVSGGSMNPVRTLGPAVAANNYKAIWVYLVAPILGALAGAGTYTAVKLPEEDDDAKAKTSISSFRR

>GmNIP3-2

MNNEEVPSLPSTSATPGTPGAPLFGGLRFEKPNGSVVRKSSFLKSCKCFSVAEWTLEDGAMPRVSCSLPSPHIPLAKKIGAEFIGTFILMFAAIGTAIVNQKTHGSETLIGCAAANGLAVMIIIFSTGHISGAHLNPAVTISFAALKHFPWKNVPVYIGTQVLASVSAAFALKVVFHPFMSGGVTVPSVGYGQAFATEFIVSFILMFVVTAVATDTRAVGELAGIAVGATVMLNILIAGPTTGSSMNPVRTLGPAIAANNYKGIWVYLIAPILGTLCGAGAYTVVKLPEEEATKTPSSAPNGSFRR

>GmNIP3-3

MPESETGTPTAASVPATPDTPGGPLFTSLRVDSLSHERDSFAMARCKCLPTKGHICFTDFSVGVPLPNVSLTQKVGAEFVGTFILIFAATAGPIVNNKYNGVETLMGNAACAGLTVMFIILSIGHISGAHLNPSLTIAFAAFRHFPWAHVPAYIAAQVSASICACYALKGNWWCYAVQVGELAGIAVGATVLLNILISGPTSGGSMNPVRTLGPAVAAGNYKHIWIYLVAPTLGALAGAGVYTLVKLRDNEAEPPRQVRSFRR

>GmNIP4-1

MTDIFEKHQSSDSSNYASSSGLCEEDKEIGYRAATSKHRYVLANNSALKFIPIKIDLNCARMVMAEVVGTFILMFCVCGITASTRFQNGAVGLLEYAATAGLTVVVIIFSIGPISCAHVNPAVTIAFATIGQFPWLKVPVYIIAQTVGSMSATYVGSLVYGIKSDAMMTMPLQGCNSAFWVEVIATFIIMFLVAALTSESQSVGHLSGFVAGMAIGLAVLITGPVSGGSMNPARSLGPAILSWKFKNIWIYMVAPSGGAIAGAAMFRFLRLRDQHSSTLSSPNIIDVGRSIPFCSRRSGPMILLVENNWSLSYGRVEGFRQRYVLRTKGISDGVYHKFPL

>GmNIP4-2

MEYEVTAELVGTFILMFCVCGITASTRFQNGAVGLLEYAAIAGLTVVVIIFSIGPISCAHVNPAVTIAFATIGQFPWFKVPVYIIAQTVGSMSATYIGSLVYGIKSEAMMTMPLQGCNSAFWVEVIATFIIMFLIAALTSESQSVGHLSGFVAGMAIGLAVLITGPVSGGSMNPARSLGPAILSWKFKNIWIYMVAPSGGAVAGAAMFRFLRLRDQHSSILSSPNISDVGRSLPFCSRRSGPMILLVKKNWSSFSERVEGFRQRCVLRTKGISEGVYHKLPL

>GmNIP5-1

MADSLSVNFDSSIKSEFSTEQAHKTTHEAKHSPSNIQKAIAEVVGTYILIFAGCGAALVNEKLPLTIVGIAMVSGLGLTVATYSVGHVSGGHFNPAVTIALAAVRKVQFKLMMGATLAPLTLKVLYHDKADIGVTVTKYLSSTSDLEAIVWEFITTSILMLTIRGVATDHRGSKDLTGVAIGISVLINVIIAGPITGASMNPARSLGPAIVSGDYKNIWVYIISPILGAVSASTLYKFLEGRPSGGGIWSSMHGVLVQTSLRPLYPEKPSNYTLPCT

>GmPIP1-1

MEGKEEDVRVGANRYGERQPIGTAAQAKDYREPPSAPLFEPGELSSWSFYRAGIAEFVATFLFLYITVLTVMGVFKSKSKCSTVGIQGIAWAFGGMIFALVYSTAGISGGHINPAVTFGLFLARKLSLTRAIFYIIMQCLGAICGAGVVKGFEPHLYERLGGGANTIAKGYTNSAGLGAEIVGTFVLVYTVFSATDAKRNARDSHVPILAPLPIGFAVFLVHLATIPVTGTGINPARSLGAAIIFNKDQAWDDHWIFWVGPFIGAALAALYHQIVIRAIPFSSK

>GmPIP1-2

MEGRDEDVRVGANRYGERQPIGTAAQTQDAKDYREAPPAPLFEPRELTSWSFYRAGIAEFVATFLFLYVTVLTVMGVAKSPSKCSTVGVQGIAWSFGGMIFALVYCTAGISGGHINPAVTFGLFLARKLSLTRTVFYMIMQCLGAICGAAVVKGFQSNQYERLGGGANTLSKGYSKGDGLGAEIVGTFILVYTVFSATDAKRNARDSHVPILAPLPIGFAVFLVHLATIPITGTGINPARSLGAALVYNKDQAWDNHWIFWVGPFIGAALAALYHQIVLRAIPFKSK

>GmPIP1-3

MEREEDVKVGAQKFSERQALGTGAKSDKDYKEAPPAPLFEPGELKSWSFYRAGIAEFVATFLFLYITVLTVMGVNRAPNKCSSVGIQGIAWAFGGMIFALVYCTAGISGGHINPAVTFGLFLARKLSLTRAVFYIVMQCLGAICGAGVVKGFEGNARYELFKGGANFVSHGYTKGDGLGAEIVGTFILVYTVFSATDAKRNARDSHVPILAPLPIGFAVFLVHLATIPITGTGINPARSLGAAIIYNRDHAWDDHWIFWVGPFIGAALAAVYHQIVIRAIPFKTRG

>GmPIP1-4

MEREEDVKVGAQKFSERQALGTGAQGDKDYKEAPPAPLFEPGELKSWSFYRAGIAEFVATFLFLYITVLTVMGVNRAPNKCSSVGIQGIAWAFGGMIFALVDCTAGISGGHINPAVTFGLFLARKLSLTRALFYIVMQCLGAICGAGVVKGFEGNARYELFKGGANFVSHGYTKGDGLGAEIVGTFILVYTVFSATDAKRNARDSHVPILAPLPIGFAVFLVHLATIPITGTGINPARSLGAAIIYNRDHAWDDHWIFWVGPFIGAALAALYHQIVIRAIPFKTRG

>GmPIP1-5

MESKEEDVRVGATKFSERQPIGTAAQGDKDYKEPPPAPLFEPGELKSWSFYRAGIAEFVATFLFLYITILTVMGVNRSPSKCASVGIQGIAWAFGGMIFALVYCTAGISGGHINPAVTFGLFLARKLSLTRALFYIIMQCLGAICGAGVVKGFEGNARYEMFKGGANFVNSGYTKGDGLGAEIVGTFVLVYTVFSATDAKRNARDSHVPILAPLPIGFAVFLVHLATIPITGTGINPARSLGAAIIYNRDHAWDDQWIFWVGPFIGAALAAVYHQIVIRAIPFKTRA

>GmPIP1-6

MESKEEDVNVGANKFSERQPIGTAAQGGGDKDYKEAPPAPLFEPGELKSWSFYRAGIAEFVATFLFLYITILTVMGVNRSPSKCASVGIQGIAWAFGGMIFALVYCTAGISGGHINPAVTFGLFLARKLSLTRALFYIIMQCLGAICGAGVVKGFEGNANYELFKGGANFVNSGYTKGDGLGAEIVGTFVLVYTVFSATDAKRNARDSHVPILAPLPIGFAVFLVHLATIPITGTGINPARSLGAAIIYNRDHAWDDQWIFWVGPFIGAALAAVYHQIVIRAIPFKTRA

>GmPIP1-7

MEGKEEDVSLGANKFSERQPIGTAAQSQDDGKDYTEPPPAPLFEPSELTSWSFYRAGIAEFVATFLFLYITILTVMGVNRSSSKCATVGIQGIAWAFGGMIFALVYCTAGISGGHINPAVTFGLFLARKLSLTRALFYMVMQVLGAIVGAGVVKGFEGKTFYGQHNGGANFVAPGYTKGDGLGAEIVGTFILVYTVFSATDAKRSARDSHVPILAPLPIGFAVFLVHLATIPITGTGINPARSLGAAIIFNKDLGWDDHWIFWVGPFVGAALAALYHQVVIRAIPFKSS

>GmPIP1-8

MEGKEQDVSLGANKFPERQPIGTAAQSQDDGKDYQEPAPAPLVDPTEFTSWSFYRAGIAEFVATFLFLYITVLTVMGVAGAKSKCSTVGIQGIAWAFGGMIFALVYCTAGISGGHINPAVTFGLFLARKLSLPRAIFYIVMQCLGAICGAGVVKGFEGKTKYGALNGGANFVAPGYTKGDGLGAEIVGTFILVYTVFSATDAKRSARDSHVPILAPLPIGFAVFLVHLATIPITGTGINPARSLGAAIIFNKDLGWDEHWIFWVGPFIGAALAALYHQVVIRAIPFKSK

>GmPIP2-1

MAKHDVEGGSFSAKDYHDPPPAPLIDAEELTQWSFYRALIAEFIATMLFLYITVLTVIGYKSQSDVKAGGDVCGGVGILGIAWAFGGMIFILVYCTAGISGGHINPAVTFGLFLARKVSLIRAIMYMVAQCLGAICGVGLVKAFQKAYYNRYGGGANELSEGYSTGVGLGAEIIGTFVLVYTVFSATDPKRNARDSHVPVLAPLPIGFAVFMVHLATIPVTGTGINPARSLGAAVMYNQQKAWDDHWIFWVGPFIGAAIAAFYHQFILRAGAAKALGSFRSNPAI

>GmPIP2-2

MAKHDVEGGSFAAKDYHDPPPAPLIDAEELTQWSFYRALIAEFIATMLFLYITVLTVIGYKSQSDVKAGGDVCGGVGILGIAWAFGGMIFILVYCTAGISGGHINPAVTFGLFLARKVSLIRAIMYMVAQCLGAICGVGLVKAFQKAYYNRYGGGANELSEGYSTGVGLGAEIIGTFVLVYTVFSATDPKRNARDSHVPVLAPLPIGFAVFMVHLATIPVTGTGINPARSLGAAVMYNQQKAWDDHWIFWVGPFIGAAIAAFYHQFILRASAAKALGSFRSNPTI

>GmPIP2-3

MAKHDVEGGSFSAKDYHDPPPAPLIDAEELTQWSFYRALIAEFIATLLFLYITVLTVIGYKSQSDVKAGGDVCGGVGILGIAWAFGGMIFILVYCTAGISGGHINPAVTFGLFLARKVSLIRAIMYMVAQCLGAICGVGLVKAFQKAYYNRYGGGANELSEGYSTGVGLGAEIIGTFVLVYTVFSATDPKRNARDSHVPVLAPLPIGFAVFMVHLATIPVTGTGINPARSLGAAVMYNQQKAWDDHWIFWVGPFIGAAIAAFYHQFILRAGAAKALGSFRSNPTI

>GmPIP2-4

MAKHDVEGGSFSAKDYHDPPPAPLIDAEELTQWSFYRALIAEFIATLLFLYITVLTVIGYKSQSDVKAGGDVCGGVGILGIAWAFGGMIFILVYCTAGISGGHINPAVTFGLFLARKVSLIRAIMYMVAQCLGAMCGVGLVKAFQKAYYNRYGGGANELSEGYSTGVGLGAEIIGTFVLVYTVFSATDPKRNARDSHVPVLAPLPIGFAVFMVHLATIPVTGTGINPARSFGAAVMYNQKKAWDDQWIFWVGPFIGAAIAAFYHQFILRASAAKAVGSFRSNPTI

>GmPIP2-5

MSVFWQEGGMAKDVEVAERGSFSGKDYQDPPPAPLIDAEELTKWSFYRALIAEFIATLLFLYITVLTVIGYNHQTDLKENGEICGGVGILGIAWAFGGMIFILVYCTAGISGGHINPAVTFGLFLARKVSLIRAIMYMVAQCLGAICGVGLVKAFQKSYFNKYGGGANSLAAGYSTGTGLGAEIIGTFVLVYTVFSATDPKRNARDSHVPVLAPLPIGFAVFMVHLATIPVTGTGINPARSLGAAVIYNQDKPWDDHWIFWVGPFIGAAIAAFYHQFILRAGAAKALGSFRSNPHN

>GmPIP2-6

MAKDVEVAERGSFSGKDYQDPPPAPLIDAEELTKWSFYRALIAEFIATLLFLYITVLTVIGYKHQTDHADACGGVGILGIAWAFGGMIFILVYCTAGISGGHINPAVTFGLFLARKVSLIRAIMYMVAQCLGAICGVGLVKAFQKSYFNKYGGGANSLADGYSTGTGLGAEIIGTFVLVYTVFSATDPKRNARDSHVPVLAPLPIGFAVFMVHLATIPVTGTGINPARSLGAAVIYNQDKPWDDHWIFWVGPFIGAAIAAFYHQFILRAGAAKALGSFRSNPHN

>GmPIP2-7

MAKDVEVQEQGGEYSAKDYHDPPPAPLFDPEELTQWSFYRALIAEFIATLLFLYVTVLTIIGYKRQTDATLGGTECDGVGILGIAWAFGGMIFILVYCTAGISGGHINPAVTFGLFLGRKVSLIRALLYMVAQCAGAICGTGLAKGFQKSYYNRYGGGANSVADGYNNGTALGAEIIGTFVLVYTVFSATDPKRNARDSHVPVLAPLPIGFAVFMVHLATIPITGTGINPARSFGAAVIYNKDKIWDDQWIFWVGPIVGAAVAAFYHQYILRAAAIKALGSFRSNA

>GmPIP2-8

MAKDVEVQEQGGEYSAKDYHDPPPAPLFDPEELTQWSFYRALIAEFIATLLFLYVTVLTIIGYKRQTDTTVGGTDCDGVGILGIAWAFGGMIFILVYCTAGISGGHINPAVTFGLFLGRKVSLIRALLYMVAQCAGAICGTGLAKGFQKAYYNRYGGGANSVADGYNNGTALGAEIIGTFVLVYTVFSATDPKRNARDSHVPVLAPLPIGFAVFMVHLATIPITGTGINPARSFGAAVIYNEDKIWDDQWIFWVGPIVGAAVAAFYHQYILRAAAIKALGSFRSNA

>GmPIP2-9

MAKDVEQVTEQGEYSAKDYHDPPPAPLIDPDELTKWSLYRAAIAEFIATLLFLYITVLTIIGYKRQSDTKIPGNTECDGVGILGIAWAFGGMIFILVYCTAGISGGHINPAVTFGLFLGRKVSLVRALLYMIAQCAGAICGAGLAKGFQKSYYNRYGGGVNTVSDGYNKGTALGAEIIGTFVLVYTVFSATDPKRSARDSHVPVLAPLPIGFAVFMVHLATIPVTGTGINPARSFGPAVIFNNDKAWDDQWIYWVGPFVGAAVAAFYHQYILRAAAIKALGSFRSNT

>GmPIP2-10

MAKDVEQVTEQGEYSAKDYHDPPPAPLIDPDELTKWSLYRAAIAEFIATLLFLYITVLTIIGYKRQSDTKIPGNTECDGVGILGIAWAFGGMIFILVYCTAGISGGHINPAVTFGLFLGRKVSLVRALLYMIAQCAGAICGAGLAKGFQKSFYNRYGGGVNTVSDGYNKGTALGAEIIGTFVLVYTVFSATDPKRNARDSHVPVLAPLPIGFAVFMVHLATIPVTGTGINPARSFGPAVIFNNDKAWDDQWIYWVGPFVGAAVAAIYHQYILRGSAIKALGSFRSNA

>GmPIP2-11

MAKDIETEVQSGLPHKDYHDPPAAAFYDPAELRKWSFYRALIAEFVATLLFLYVTILTVIGYNHQTATGSPDLCNGVGVLGIAWAFGGMIFVLVYCTAGISGGHINPAVTFGLFLARKVSLIRAVGYMVAQVLGAISGVGLVKALQKSYYNRYNGGVNMLADGYSKGTGLGAEIIGTFILVYTVFSATDPKRVARDSHVPVLAPLPIGFAVFIVHLATIPITGTGINPARSLGPAVIFNNEKAWDDQWIFWVGPFIGAAIAAFYHQSVLRAQAAKALGSFRSSSNL

>GmPIP2-12

MAKDLETEIQSGLPHKDYHDPPPAPFYDPAELRKWSFFRALIAEFVATLLFLYVTILTVIGYNHQTATAAEPCSGVGVLGIAWAFGGMIFVLVYCTAGISGGHINPAVTFGLFLARKVSLTRAVGYMVAQVLGAISGVGLVKALQKSYYNRYKGGVNMLADGYSKGTGLGAEIIGTFILVYTVFSATDPKRVARDSHVPVLAPLPIGFAVFMVHLATIPITGTGINPARSLGPAVIFNNEKAWDDQWIFWVGPFIGAALAAFYHQSVLRAQAAKALGSFRSSSNL

>GmPIP2-13

MSKEVSQQRKDYVDPPPAPLIDLAEIKLWSFYRALIAEFIATLLFLYVTVATVIGHKKQTGPCDGVGLLGIAWAFGGMIFVLVYCTAGISGGHINPAVTFGLFLARKVSLIRALFYMVAQCLGAICGVGLVKAFMKHSYNSLGGGANSVSAGYNKGSALGAEIIGTFVLVYTVFSATDPKRSARDSHIPVLAPLPIGFAVFMVHLATIPITGTGINPARSFGAAVIYNNGKVWDDHWIFWVGPFVGALAAAAYHQYILRAAAIKALGSFRSNPTN

>GmPIP2-14

MSKEVSQEGLQRKDYVDPPPAPLFDLAEIKLWSFYRALIAEFIASLLFLYVTVATIIGHKKQTGPCDGVGLLGIAWSFGGMIFVLVYCTAGISGGHINPAVTFGLFLARKVSLIRAVFYMVAQCLGAICGVGLVKAFMKHSYNSLGGGANSVSAGYNKGSALGAEIIGTFVLVYTVFSATDPKRSARDSHVPVLAPLPIGFAVFMVHLATIPITGTGINPARSLGAAVIYNNGKVWDEHWIFWVGPLVGALAAAAYHQYILRAGAIKALGSFRSNPTN

>GmSIP1-1

MVSAIKAAIGDLVLTFLWVFFSSMLGLATNTITTALDLHHVSYNGFDYPSAVIITSLIFILVTIFTFVGNALGGASFNPTANASSYAAGLGSDSLFSMALRFPAQALGSVGGVLAVMEVMPPKYRHLIGGPSLKVSLHTGAIAEGVLTFVITFVVLLIMIRGPRSEAVKTWLMAISTVVLITAGSAYTGPAMNPAFAFGWAYFENWHNTWDQFYVYWICPFFGAILAAWLFRIVIPPAPRVVKQKKA

>GmSIP1-2

MASAIKAAIGDLVLTFLWVFFSSMLGLVTNAITTALDLHHVSYNGFDYPSAVIITSLIFILVTIFTFVGNALGGASFNPTGNASSYAVGLGSDTLFSMALRFPAQALGSVGGVLAVMEVMPPKYRHLIGGPSLKVSLHTGAIAEGVLTFVITFVVLLIMIRGPRSEAVKTLLMAISTVVLITAGSAYTGPAMNPAFAFGWAYFENWHNTWDQFYVYWICPFFGAILAAWLFRIVFPPRVVKQKKA

>GmSIP1-3

MVGAIKAAIGDAVLTFMWVFCSSVLGIASGYITNALNLQHITYNGFPYPSFLVTTTLVFVLVFLFTIIGNVLGGASFNPTGTASFYAVGLGSDTLFSMALRFPAQAAGAAGGALAIMEVIPAKYRHMIGGPSLKVDLHTGAVAEGVLTFVITFVVLLIFLKGPRSDLLKTWLLATATVVLVMVGSAYTGPAMNPANAFGWAYINNWHNTWDQFYVYWICPFAGAILAAWLFRAVFPPPSPPEVKQKKA

>GmSIP1-4

MVGAIKAAIGDAVLTFMWVFCSSVLGIASGYITNALNLQHITYNGFPYASFLVTTTLVFVLVFLFTIIGNVLGGASFNPTGTASFYAVGLGSDTLFSMALRFPAQAAGAAGGAMAIMEVIPAKYRHMIGGPSLKVDLHTGAVAEGVLTFVITFAVLLIFLRGPRSDLLKTWLLATATVVLVMVGSAYTGPAMNPANAFGWAYLNNWHNTWDQFYVYWICPFTGAILAAWLFRAVFPPPPPPEVKQKKA

>GmSIP1-5

MGLIKAAIGDGVLTSMWVFIISTLRIVTTEVALFLGLQPLSLAGLIISTILNSFYVLTISFIGRILGGANFNPSTSLSFYTAGLRPDSSLSSMAVRFPVQAYGGAVGVKTLLLVMPSKYNDMLKGPFLKVDLHSGAVAEGVLTFTHNMAIFFVMFKGPRNPFVKVYLLSVTTAVLAILGGGFTGPSMNPANAFGWAFVNNKHNTWEQFYVYWICPFIGASSAALIFRSMFMPPIKQKKA

>GmSIP1-6

MGWIKAAIGDAILTSMWVFIISTLRIVTTEITVFLGLQPFFLAGLIISTILNSIYVLTISFIGRILGGVSFNPSTSLSFYTAGLRPDSSLSSMAVRFPAQAYGGAVGIKTLLLVMPSHYKDMLKGPFLKVDLHSGAVAEGLLTFIHNMAIFFVMFKGPRNPFVKVYLLSVTTAALAILGGGFTGPSMNPANAFGWAFVNNKHNTWEQFYVYWIGPFIGASSAALIFRSMFMPPIKQKKA

>GmSIP2-1

MGRARLLVSDFVLSFMWVWSGVLLRILVFKHLGFAHGPLGEVIKTTFSVANMFFFAFLVKVTRGAAYNPLTVLADAITGDFNTFLYCVGARIPAQVVGSIVGVKLLIDTIPEVGVGPRLNVDIHQGSLTEGLLTFAIVTISLGLATKIRENFFMKTWISSLSKLTLHILGSDLTGGCMNPASVMGWAYARGDHITKEHFLVYWLAPIEATIFAVWTSKFLVQPGKEHKKA

>GmSIP2-2

MGRARLLVSDFVLSFMWVWSGVLLRIIVFNHLGFAHGPLGEVIKTTFSIANMFFFAFLVKVTRGGAYNPLTVLADAISRDFNNFLYCAGARIPTQVVGSIVGVKLLIDTIPEVGLGPRLNVDIHRGALTEGLLTFAIVTISLGLASKIRENFFMKTWISSLSKLTLHILGSDLTGGCMNPASVMGWAYARGDHITKEHFLVYWLAPIEATILAVWTFKFLVQPGKEDKSTSKSKSD

>GmTIP1-1

MAYRSAIVRRAQEASHRDTWRAALSEFISTLIFVFAGSGSSVAVNKLTVDKPSALVVAAVAHAFALFVAVSVSTNISGGHVNPAVTFGAFVGGNLTLLRCVLFWIAQILGSVIACLLLKFITGGQDVPVFKLSSGVGVGNAVVLEMVMTFGLVYTVYATTVDPRSRRGSLGVMAPIVIGFIVGANVLVGGPFDGASMNPAASFGPAVVGWSWKNHWVYWVGPLVGGGLAGFMYELIFVSHSRQRFRRSYY

>GmTIP1-2

MPIRNIAVGRPEEATHPDTLKAALAEFISTFIFVFAGSGSGIAYNKLTDNGAATPAGLISASIAHAFALFVAVSVGANISGGHVNPAVTFGAFVGGNITFLRGIVYVIAQLLGSIVASLLLAFVTASTVPAFGLSAGVGVGNALVLEIVMTFGLVYTVYATAIDPKKGNLGIIAPIAIGFIVGANILLGGAFSGAAMNPAVTFGPAVVSWTWTNHWIYWAGPLIGGGIAGLVYEVVFISHTHEQLPTTDY

>GmTIP1-3

MPIRNIAIGRPEEATHPDTLKAGLAEFISTLIFVFAGSGSGIAYNKLTDNGAATPAGLISASIAHAFALFVAVSVGANISGGHVNPAVTFGAFVGGNITLLRGIVYVIAQLLGSIVASLLLAFVTASPVPAFGLSAGVGVGNALVLEIVMTFGLVYTVYATAVDPKKGNLGIIAPIAIGFIVGANILLGGAFSGAAMNPAVTFGPAVVSWTWTNHWIYWAGPLIGGGIAGLIYEVVFISHTHEQLPSTDY

>GmTIP1-4

MPISRIAIGNPSEFGQADALKAALAEFISMLIFVFAGEGSGMAYNKLTDNGSATPAGVVAASLSHAFALFVAVSVGANISGGHVNPAVTFGAFIGGHISLLRGILFWIAQLLGSVVACLLLKFATVGLSPGVGAANALVFEIVMTFGLVYTVYATAVDPKKGKLGIIAPIAIGFIVGANILAGGTFSGASMNPAVSFGPAVVSGTWANHWVYWAGPLIGSAIAAVVYETFFITPNSYEQLPVTDY

>GmTIP1-5

MPISRIAIGNPSEFGQADALKAALAEFISMLIFVFAGEGSGMAYNKLTDNGSATPAGVVAASLSHAFALFVAVSVGANISGGHVNPAVTFGAFIGGHISLLRGILYWIAQLLGSVVACLLLKFATGGLETSAFSLSPGVGAANALVFEIVMTFGLVYTVYATAVDPKKGNLGIIAPIAIGFIVGANILAGGAFDGASMNPAVSFGPAVVSGTWANHWVYWVGPLIGSAIAAIIYETFFITPNSYEHLPVTDY

>GmTIP1-6

MPISRIAIGNSSELNQSDALKAALAEFISMLIFVFAGEGSGMAYNKLTNNGSATPAGLVAASLSHAFALFVAVSVGANISGGHVNPAVTFGAFVGGHITLFRSILYWIAQLLGSVVACLLLKFATGGLETSAFALSPGVEAGNALVFEIVMTFGLVYTVYATAVDPKKGDLGIIAPIAIGFIVGANILAGGAFDGASMNPAVSFGPAVVSWTWSNHWVYWVGPFAGAAIAAVVYEIFFISPNTHEQLPVTDY

>GmTIP1-7

MAVYRIAIGSPREASNPAAIRAAFAEFFSMLIFVFAGQGSGMAYSKLTGNGPATPGGLVVASLSHTFGLFVAVAVGANISGGHVNPAVTFGAFIGGNITLLRSILYWIAQLLGSVVACILLKVATGGMETSAFSLSSGVSVWNALVFEIVMTFGLVHTVYATTVDPKKGNVGVIGPIAIGSIVGANILVGGAFDGASMNPAVCFGPALINWSWTHHWVYWLGPFIGSATAAILYDNIFIGDDGHEPLSNSDF

>GmTIP1-8

MAMHRIAIGTPGEAAQPDAIRAAFAEFFCMIIFVFAGEGSGMAYSKLTNNGPATPAGLIAASLSHAFGLFVAVSVGANISGGHVNPAVTFGAFIGGNITLLRSILYWIAQLFGSVVACILLKHATGGMETSGFSLSPGVSVWNALVFEIVMTFGLVYTVYATAVDPKKGNAGVVAPIAIGFIVGANILVGGAFDGASMNPAVSFGPAVVTWSWTHHWVYWVGPFIGAAIAAIIYDNIFIGDDGHEPLSSSDF

>GmTIP1-9

MAIYRIAIGTPGEAGQPDAIRAAFAEFFSMIIFVFAGEGSGMAYSKLTNNGPATPAGLIAASLSHAFGLFVAVSVGANISGGHVNPAVTFGAFIGGNITLLRSILYWIAQLLGSVVACILLKSATGGMETTGFSLSPGVSVWNALVFEIVMTFGLVYTVYATAVDPKKGNVGVVAPIAIGFIVGANILVGGAFDGASMNPAVSFGPAVVTWSWTHHWVYWVGPFIGAAIAAVIYDNIFIGDDGHEPLSSSDF

>GmTIP2-1

MAGIAFGSFNDSVSFASIKAYIAEFISTLLFVFAGVGSAIAYAKLTSDAALDPTGLVAVAICHGFALFVAVSVGANISGGHVNPAVTFGLALGGHITILTGLFYWIAQLLGSIVASLLLKFVTGYDTPIHSVAAGIGAGEGVVTEIIITFGLVYTVYATAADPKKGSLGTIAPIAIGFIVGANILAAGPFSGGSMNPARSFGPAVVSGDFHDNWIYWVGPLIGGGLAGLIYTYAFIPTNHAPLATEF

>GmTIP2-2

MAGIAFGSFNDSFSLASIKAYIAEFISTLLFVFAGVGSAIAYAKLTSDAALDPTGLVAVAICHGFALFVAVSVGANISGGHVNPAVTFGLALGGHITILTGLFYWIAQLLGSIVASLLLKFVTGYDTPIHSVAAGVGAGEGVVTEIIITFGLVYTVYATAADPKKGSLGTIAPIAIGFIVGANILAAGPFSGGSMNPARSFGPAVVSGDFHDNWIYWVGPLIGGGLAGLIYTYAFIPTQHAPLATDF

>GmTIP2-3

MAGIAFGNFNDSVSFASIKAYIAEFISTLLFVFAGVGSAIAYAKLTSDAALDPTGLVAVAICHGFALFVAVSVGANISGGHVNPAVTFGLALGGHITILTGLFYWIAQLLGSIVASLLLKFVTGYDTPIHSVAAGIGAGEGVVTEIIITFGLVYTVYATTADPKKGSLGTIAPIAIGFIVGANILAAGPFSGGSMNPARSFGPAVVSGDFHDNWIYWVGTLIGGGLAGLIYTYAFIM

>GmTIP2-4

MGGIAFGRFDDSFSLTSIKAYIAEFHSTLLFVFAGVGSAIAYGKLTSDAALDPAGLLAVAICHGFALFVAVSVGANISGGHVNPAVTFGLALGGHITILTGFFYWIAQLLGSIVACFLLNYVTGGLPTPIHSVASGVGAVEGVVTEIIITFGLVYTVYATAADPKKGSLGIIAPIAIGFIVGANILAAGPFSGGSMNPARSFGPAVVSGDFHDNWIYWVGPLIGGGLAGLIYGNVFIRSDHAPLSSEF

>GmTIP2-5

MGGIAFGRLDDSFSLTSIKAYIAEFHSTLLFVFAGVGSAIAYGKLTSDAALDPAGLLAVAICHGFALFVAVSVGANISGGHVNPAVTFGLALGGHITILTGFFYWIAQLLGSIVACFLLNYVTGGLPTPIHSVASGVGAVEGVVTEIIITFGLVYTVYATAADPKKGSLGTIAPIAIGFIVGANILAAGPFSGGSMNPARSFGPAVVSGDFHDNWIYWVGPLIGGGLAGLIYGNVFIRSDHAPLSSEF

>GmTIP2-6

MVKITLGTFDDSFGVASLKAYLAEFHATLIFVFAGVGSAIAYNELTKDAALDPTGLVAVAVAHAFALFVGVSVAANISGGHLNPAVTFGLAIGGNITLITGFLYWIAQLLGSIVACLLLNFITAKSIPSHAPATGVNDFQAVVFEIVITFGLVYTVYATAADPKKGSLGIIAPIAIGFVVGANILAAGPFSGGSMNPARSFGPAVVSGDFAANWIYWVGPLIGGGLAGLIYGDVFIGSYAAVPASETYP

>GmTIP2-7

MVKIALGTLDDSFSAASLKAYFAEFHATLIFVFAGVGSAIAYNELTKDAALDPTGLVAVAVAHAFALFVGVSVAANISGGHLNPAVTFGLAIGGNITLITGFLYWIAQLLGSIVACLLLNLITAKSIPSHSPANGVNDLQAVVFEIVITFGLVYTVYATAVDPKKGSLGIIAPIAIGFVVGANILAAGPFSGGSMNPARSFGPAVVSGDLAANWIYWVGPLIGGGLAGLIYGDVFIGSYAPVPASETYP

>GmTIP3-1

MATRRYEFGRMNEASHPDSIRAALVEFLSTCIFVFAGEGSALALRQIYKEPGSSAGELVVIALAHAFALFAAISASMHVSGGHVNPAVTFGALLGGRISVLRALYYWVAQLLGSIVAALLLRLVTNNMRPQGFSVSIGLGAFHGLILEIALTFGLMYTVYATAIDPKRGSIGSIAPLAIAFVVGANILAGGPFDGACMNPARAFGPAMVGWRWHYHWIFWVGPLIGAALAALLYEYVMVPIEPPHHQPLAGVDY

>GmTIP3-2

MATRRYEFGRMNEASHPDSIRAALAEFLSTCIFVFAGEGSALALRQIYKEPGSSAGELVVIALAHAFALFAAISASMHVSGGHVNPAVTFGALLGGRISVLRAVYYWVAQLLGSIVAALLLRLVTNNMRPQGFSVSIGLGAFHGLVLEIALTFGLMYTVYATAIDPKRGSIGSIAPLAIGFVVGANILAGGPFDGACMNPARAFGPAMVGWRWHYHWIFWVGPFIGAALAALLYEYVMVPNEPPHHQPLAAEDY

>GmTIP3-3

MATRRYSFGRADEATHPDSMRATLAEFASTFIFVFAGEGSSLALVKIYQDSAFSAGELLAVALAHAFALFAAVSSSMHVSGGHVNPAVTFGALIGGRISVLRAVYYWIAQILGAIVAALVLRLVTNNMRPSGFHVGQGVGVGHMLILEIIMTFGLMYTVYGTAIDPKRGSVSNIAPLAIGLIVGANILVGGPFDGACMNPALAFGPSLVGWRWHQHWIFWVGPLIGAALAALVYEYVVIPTEPPHQHQPLAPEDY

>GmTIP3-4

MATRRYAFGRADEATHPDSMRATLAEFVSTFIFVFAGEGSGLALVKIYQDSAFSAGELLAVALAHGFALFAAVSASMHVSGGHVNPAVTFGALIGGRISVLRAVYYWIAQILGAIVAALVLRLVTNNMRPSGFHVGQGVGVGHMLILEIVMTFGLMYTVYGTAIDPKRGAVSNIAPLAIGLIVGANILVGGPFDGACMNPALAFGPSLVGWRWHQHWIFWVGPLIGAALAALVYEYVVIPTEPPHQHQPLAPEDY

>GmTIP4-1

MARIALGSTREATQPDCIQALIVEFIATFLFVFVGVASSMVVDKLGGDALVGLFAVAVAHALVVAVMISAAHISGGHLNPAVTLGLLAGGHITIFRSLLYWIDQLVAAAAASYLLYYLSGGQATPVHTLASGVGYGQGVVWEIVLTFSLLFTVYATMVDPKKGALAGLGPTLVGFVVGANILAGGAYSAASMNPARSFGPALVTGNWTDHWVYWVGPLIGGGLAGFIYETFFIDRSHVPLPRDEES

>GmTIP4-2

MAKIALGSTREATQPDCIQALIVEFIATFLFVFVGVGSSMVVDKLGGDALVGLFAVAVAHALVVAVMISAAHISGGHLNPAVTLGLLAGGHITIFRSMLYWIDQLVAAATASYLLYYLSGGQATPVHTLASGVGYGQGVVWEIVLTFSLLFTVYATMVDPKKGALAGLGPTLVGFVVGANILAGGAYSAASMNPARSFGPALVAGNWTDHWVYWVGPLIGGGLAGYIYETFFIDRSHVPLPRDEEN

>GmTIP5-1

MAPSSVTVTSRFHESVTRNALRSYLSEFISTFFYVFLVIGAGMSSRKLMPDASLNPTSLVVVGIGSAFALSSVLYIAWDISGGHVNPAVTFAMAVGGHISVPTALFYWVAQLIASVMACLVLRVIVVGMHVPTYTIAEEMTGFGASVLEGTLTFVLVYTVYAARDPRRGPMSSTGILVVGLIAGASVLASGPFSGGSMNPACAFGSAAIAGSFRNQAVYWVGPLIGATIAGLLYDNELGSKLCSVMQ

>GmXIP1-1

MDDQFSTVHSHSREPMPRGLVHEKSSEPKFLAYIGAHEFFTIETWKAALVELIATAALMFTLTSCNIACLESQDVNPKLILPFAVFIIVFLFLIVIVPLSGGHMNPIFTFIAALKGVVTLSRALLYVSAQCIGSIIGFFVLKSVMEPKLADTYSLGGCALGDKGQSSGLRPQDALLLEFSCTFLVLFVGLTLAFDKKRCKELGLPMVCLVVAASLALAVFVSITVTGRPGYAGAGLSPARCLGPALLHGGPLWNGHWVFWLGPFLACIIYYSVSINLPKKGLNWVDGEYDVLRLALGSCRTISNNTDLNDLS

>GmXIP2-1

MWKAALTELTATASLMFTLTTSIIACLDSHEIDPKLLVPFAVFTIAFLFLIVTVPLTGGHMSPVFTFIAALKGVVTLTRALIYVLAQCIGSIIGFFILKCVMDPKLAYTYSLGGCAISGQGVINSSSGGIKPLDALLVEFTCTFVVLFVGVTLAFDKKRSRDLGLPMVCLVVAGAMALAVFVSITVTGRAGYAGVGLNPARCLGPALLHGGLLWEGHWVFWLGPFLACGLVWVDGEYDVLKLALGSCGNVYNTSVSKDHQLEEPSAGFQV

>AtNIP1-1

MADISGNGYGNAREEVVMVNLKDEVEHQQEMEDIHNPRPLKKQDSLLSVSVPFLQKLIAEFLGTYFLVFTGCASVVVNMQNDNVVTLPGIAIVWGLTIMVLIYSLGHISGAHINPAVTIAFASCGRFPLKQVPAYVISQVIGSTLAAATLRLLFGLDHDVCSGKHDVFIGSSPVGSDLQAFTMEFIVTFYLMFIISGVATDNRAIGELAGLAIGSTVLLNVLIAAPVSSASMNPGRSLGPALVYGCYKGIWIYLVAPTLGAIAGAWVYNTVRYTDKPLREITKSGSFLKTVRIGST

>AtNIP1-2

MAEISGNGGDARDGAVVVNLKEEDEQQQQQQAIHKPLKKQDSLLSISVPFLQKLMAEVLGTYFLIFAGCAAVAVNTQHDKAVTLPGIAIVWGLTVMVLVYSLGHISGAHFNPAVTIAFASCGRFPLKQVPAYVISQVIGSTLAAATLRLLFGLDQDVCSGKHDVFVGTLPSGSNLQSFVIEFIITFYLMFVISGVATDNRAIGELAGLAVGSTVLLNVIIAGPVSGASMNPGRSLGPAMVYSCYRGLWIYIVSPIVGAVSGAWVYNMVRYTDKPLREITKSGSFLKTVRNGSSR

>AtNIP2-1

MDDISVSKSNHGNVVVLNIKASSLADTSLPSNKHESSSPPLLSVHFLQKLLAELVGTYYLIFAGCAAIAVNAQHNHVVTLVGIAVVWGIVIMVLVYCLGHLSAHFNPAVTLALASSQRFPLNQVPAYITVQVIGSTLASATLRLLFDLNNDVCSKKHDVFLGSSPSGSDLQAFVMEFIITGFLMLVVCAVTTTKRTTEELEGLIIGATVTLNVIFAGEVSGASMNPARSIGPALVWGCYKGIWIYLLAPTLGAVSGALIHKMLPSIQNAEPEFSKTGSSHKRVTDLPL

>AtNIP3-1

MAEISDITTQTQTVVLDIENYQSIDDSRSSDLSAPLVSVSFVQKLIGEFVGTFTMIFAGCSAIVVNETYGKPVTLPGIALVWGLVVTVMIYSIGHVSGAHFNPAVSIAFASSKKFPFNQVPGYIAAQLLGSTLAAAVLRLVFHLDDDVCSLKGDVYVGTYPSNSNTTSFVMEFIATFNLMFVISAVATDKRATGSFAGIAIGATIVLDILFSGPISGASMNPARSLGPALIWGCYKDLWLYIVSPVIGALSGAWTYGLLRSTKKSYSEIIRPNCNKVSSRDRQEASQDEICVLRVVDPANQNYFICSSPTDINGKCNVTCKLA

>AtNIP4-1

MSSHSDEIEEEQISRIEKGKGKDCQGGIETVICTSPSIVCLTQKLIAEMIGTYFIVFSGCGVVVVNVLYGGTITFPGICVTWGLIVMVMIYSTGHISGAHFNPAVTVTFAIFRRFPWHQVPLYIGAQFAGSLLASLTLRLMFKVTPEAFFGTTPADSPARALVAEIIISFLLMFVISGVATDNRAVGELAGIAVGMTIMVNVFVAGPISGASMNPARSLGPALVMGVYKHIWVYIVGPVLGVISGGFVYNLIRFTDKPLRELTKSASFLRAVSPSHKGSSSKT

>AtNIP4-2

MTSHGEEIEDEQISRIEKGNCKDSQGGMETAICSSPSIVCLTQKLIAEMIGTYFIIFSGCGVVVVNVLYGGTITFPGICVTWGLIVMVMIYSTGHISGAHFNPAVTVTFAVFRRFPWYQVPLYIGAQLTGSLLASLTLRLMFNVTPKAFFGTTPTDSSGQALVAEIIISFLLMFVISGVATDSRATGELAGIAVGMTIILNVFVAGPISGASMNPARSLGPAIVMGRYKGIWVYIVGPFVGIFAGGFVYNFMRFTDKPLRELTKSASFLRSVAQKDNASKSDG

>AtNIP5-1

MAPPEAEVGAVMVMAPPTPGTPGTPGGPLITGMRVDSMSFDHRKPTPRCKCLPVMGSTWGQHDTCFTDFPSPDVSLTRKLGAEFVGTFILIFTATAGPIVNQKYDGAETLIGNAACAGLAVMIIILSTGHISGAHLNPSLTIAFAALRHFPWAHVPAYIAAQVSASICASFALKGVFHPFMSGGVTIPSVSLGQAFALEFIITFILLFVVTAVATDTRAVGELAGIAVGATVMLNILVAGPSTGGSMNPVRTLGPAVASGNYRSLWVYLVAPTLGAISGAAVYTGVKLNDSVTDPPRPVRSFRR

>AtNIP6-1

MDHEEIPSTPSTPATTPGTPGAPLFGGFEGKRNGHNGRYTPKSLLKSCKCFSVDNEWALEDGRLPPVTCSLPPPNVSLYRKLGAEFVGTLILIFAGTATAIVNQKTDGAETLIGCAASAGLAVMIVILSTGHISGAHLNPAVTIAFAALKHFPWKHVPVYIGAQVMASVSAAFALKAVFEPTMSGGVTVPTVGLSQAFALEFIISFNLMFVVTAVATDTRAVGELAGIAVGATVMLNILIAGPATSASMNPVRTLGPAIAANNYRAIWVYLTAPILGALIGAGTYTIVKLPEEDEAPKERRSFRR

>AtNIP7-1

MNGEARSRVVDQEAGSTPSTLRDEDHPSRQRLFGCLPYDIDLNPLRIVMAELVGTFILMFSVCGVISSTQLSGGHVGLLEYAVTAGLSVVVVVYSIGHISGAHLNPSITIAFAVFGGFPWSQVPLYITAQTLGATAATLVGVSVYGVNADIMATKPALSCVSAFFVELIATSIVVFLASALHCGPHQNLGNLTGFVIGTVISLGVLITGPISGGSMNPARSLGPAVVAWDFEDLWIYMTAPVIGAIIGVLTYRSISLKTRPCPSPVSPSVSSLLR

>AtPIP1-1

MEGKEEDVRVGANKFPERQPIGTSAQSDKDYKEPPPAPFFEPGELSSWSFWRAGIAEFIATFLFLYITVLTVMGVKRSPNMCASVGIQGIAWAFGGMIFALVYCTAGISGGHINPAVTFGLFLARKLSLTRALYYIVMQCLGAICGAGVVKGFQPKQYQALGGGANTVAHGYTKGSGLGAEIIGTFVLVYTVFSATDAKRNARDSHVPILAPLPIGFAVFLVHLATIPITGTGINPARSLGAAIIYNKDHSWDDHWVFWVGPFIGAALAALYHVVVIRAIPFKSRS

>AtPIP1-2

MEGKEEDVRVGANKFPERQPIGTSAQSDKDYKEPPPAPLFEPGELASWSFWRAGIAEFIATFLFLYITVLTVMGVKRSPNMCASVGIQGIAWAFGGMIFALVYCTAGISGGHINPAVTFGLFLARKLSLTRAVYYIVMQCLGAICGAGVVKGFQPKQYQALGGGANTIAHGYTKGSGLGAEIIGTFVLVYTVFSATDAKRNARDSHVPILAPLPIGFAVFLVHLATIPITGTGINPARSLGAAIIFNKDNAWDDHWVFWVGPFIGAALAALYHVIVIRAIPFKSRS

>AtPIP1-3

MEGKEEDVRVGANKFPERQPIGTSAQTDKDYKEPPPAPFFEPGELSSWSFYRAGIAEFIATFLFLYITVLTVMGVKRAPNMCASVGIQGIAWAFGGMIFALVYCTAGISGGHINPAVTFGLFLARKLSLTRAVFYIVMQCLGAICGAGVVKGFQPNPYQTLGGGANTVAHGYTKGSGLGAEIIGTFVLVYTVFSATDAKRSARDSHVPILAPLPIGFAVFLVHLATIPITGTGINPARSLGAAIIYNKDHAWDDHWIFWVGPFIGAALAALYHQLVIRAIPFKSRS

>AtPIP1-4

MEGKEEDVRVGANKFPERQPIGTSAQSTDKDYKEPPPAPLFEPGELSSWSFYRAGIAEFIATFLFLYITVLTVMGVKRAPNMCASVGIQGIAWAFGGMIFALVYCTAGISGGHINPAVTFGLFLARKLSLTRAVFYMIMQCLGAICGAGVVKGFQPTPYQTLGGGANTVAHGYTKGSGLGAEIIGTFVLVYTVFSATDAKRSARDSHVPILAPLPIGFAVFLVHLATIPITGTGINPARSLGAAIIYNKDHSWDDHWIFWVGPFIGAALAALYHQIVIRAIPFKSKS

>AtPIP1-5

MEGKEEDVNVGANKFPERQPIGTAAQTESKDYKEPPPAPFFEPGELKSWSFYRAGIAEFIATFLFLYVTVLTVMGVKRAPNMCASVGIQGIAWAFGGMIFALVYCTAGISGGHINPAVTFGLFLARKLSLTRALFYIVMQCLGAICGAGVVKGFQPGLYQTNGGGANVVAHGYTKGSGLGAEIVGTFVLVYTVFSATDAKRSARDSHVPILAPLPIGFAVFLVHLATIPITGTGINPARSLGAAIIYNKDHAWDDHWIFWVGPFIGAALAALYHQIVIRAIPFKSKT

>AtPIP2-1

MAKDVEAVPGEGFQTRDYQDPPPAPFIDGAELKKWSFYRAVIAEFVATLLFLYITVLTVIGYKIQSDTDAGGVDCGGVGILGIAWAFGGMIFILVYCTAGISGGHINPAVTFGLFLARKVSLPRALLYIIAQCLGAICGVGFVKAFQSSYYTRYGGGANSLADGYSTGTGLAAEIIGTFVLVYTVFSATDPKRSARDSHVPVLAPLPIGFAVFMVHLATIPITGTGINPARSFGAAVIYNKSKPWDDHWIFWVGPFIGAAIAAFYHQFVLRASGSKSLGSFRSAANV

>AtPIP2-2

MAKDVEGPEGFQTRDYEDPPPTPFFDADELTKWSLYRAVIAEFVATLLFLYITVLTVIGYKIQSDTKAGGVDCGGVGILGIAWAFGGMIFILVYCTAGISGGHINPAVTFGLFLARKVSLIRAVLYMVAQCLGAICGVGFVKAFQSSYYDRYGGGANSLADGYNTGTGLAAEIIGTFVLVYTVFSATDPKRNARDSHVPVLAPLPIGFAVFMVHLATIPITGTGINPARSFGAAVIYNKSKPWDDHWIFWVGPFIGAAIAAFYHQFVLRASGSKSLGSFRSAANV

>AtPIP2-3

MAKDVEGPDGFQTRDYEDPPPTPFFDAEELTKWSLYRAVIAEFVATLLFLYVTVLTVIGYKIQSDTKAGGVDCGGVGILGIAWAFGGMIFILVYCTAGISGGHINPAVTFGLFLARKVSLIRAVLYMVAQCLGAICGVGFVKAFQSSHYVNYGGGANFLADGYNTGTGLAAEIIGTFVLVYTVFSATDPKRNARDSHVPVLAPLPIGFAVFMVHLATIPITGTGINPARSFGAAVIFNKSKPWDDHWIFWVGPFIGATIAAFYHQFVLRASGSKSLGSFRSAANV

>AtPIP2-4

MAKDLDVNESGPPAARDYKDPPPAPFFDMEELRKWPLYRAVIAEFVATLLFLYVSILTVIGYKAQTDATAGGVDCGGVGILGIAWAFGGMIFVLVYCTAGISGGHINPAVTVGLFLARKVSLVRTVLYIVAQCLGAICGCGFVKAFQSSYYTRYGGGANELADGYNKGTGLGAEIIGTFVLVYTVFSATDPKRNARDSHVPVLAPLPIGFAVFMVHLATIPITGTGINPARSFGAAVIYNNEKAWDDQWIFWVGPMIGAAAAAFYHQFILRAAAIKALGSFGSFGSFRSFA

>AtPIP2-5

MTKEVVGDKRSFSGKDYQDPPPEPLFDATELGKWSFYRALIAEFIATLLFLYVTIMTVIGYKSQTDPALNPDQCTGVGVLGIAWAFGGMIFILVYCTAGISGGHINPAVTFGLLLARKVTLVRAVMYMVAQCLGAICGVALVKAFQSAYFTRYGGGANGLSDGYSIGTGVAAEIIGTFVLVYTVFSATDPKRSARDSHVPVLAPLPIGFAVFIVHLATIPITGTGINPARSLGAAIIYNKDKAWDHHWIFWVGPFAGAAIAAFYHQFVLRAGAIKALGSFRSQPHV

>AtPIP2-6

MTKDELTEEESLSGKDYLDPPPVKTFEVRELKKWSFYRAVIAEFIATLLFLYVTVLTVIGFKSQTDINAGGGACASVGLLGISWAFGGMIFILVYCTAGISGGHINPAVTFGLFLASKVSLVRAVSYMVAQCLGATCGVGLVKVFQSTYYNRYGGGANMLSDGYNVGVGVGAEIIGTFVLVYTVFSATDPKRNARDSHIPVLAPLPIGFSVFMVHLATIPITGTGINPARSFGAAVIYNNQKAWDDQWIFWVGPFVGAAIAAFYHQFVLRAGAMKAYGSVRSQLHELHA

>AtPIP2-7

MSKEVSEEGKTHHGKDYVDPPPAPLLDMGELKSWSFYRALIAEFIATLLFLYVTVATVIGHKKQTGPCDGVGLLGIAWAFGGMIFVLVYCTAGISGGHINPAVTFGLFLARKVSLVRALGYMIAQCLGAICGVGFVKAFMKTPYNTLGGGANTVADGYSKGTALGAEIIGTFVLVYTVFSATDPKRSARDSHIPVLAPLPIGFAVFMVHLATIPITGTGINPARSFGAAVIYNNEKAWDDQWIFWVGPFLGALAAAAYHQYILRASAIKALGSFRSNATN

>AtPIP2-8

MSKEVSEEGRHGKDYVDPPPAPLLDMAELKLWSFYRAIIAEFIATLLFLYVTVATVIGHKNQTGPCGGVGLLGIAWAFGGMIFVLVYCTAGISGGHINPAVTFGLFLARKVSLPRAVAYMVAQCLGAICGVGLVKAFMMTPYKRLGGGANTVADGYSTGTALGAEIIGTFVLVYTVFSATDPKRSARDSHVPVLAPLPIGFAVFMVHLATIPITGTGINPARSFGAAVIYNNEKAWDDHWIFWVGPFVGALAAAAYHQYILRAAAIKALASFRSNPTN

>AtSIP1-1

MMGVLKSAIGDMLMTFSWVVLSATFGIQTAAIISAGDFQAITWAPLVILTSLIFVYVSIFTVIFGSASFNPTGSAAFYVAGVPGDTLFSLAIRLPAQAIGAAGGALAIMEFIPEKYKHMIGGPSLQVDVHTGAIAETILSFGITFAVLLIILRGPRRLLAKTFLLALATISFVVAGSKYTGPAMNPAIAFGWAYMYSSHNTWDHIYVYWISSFVGALSAALLFRSIFPPPRPQKKKQKKA

>AtSIP1-2

MSAVKSALGDMVITFLWVILSATFGIQTAAIVSAVGFHGITWAPLVISTLVVFVSISIFTVIGNVLGGASFNPCGNAAFYTAGVSSDSLFSLAIRSPAQAIGAAGGAITIMEMIPEKYKTRIGGKPSLQFGAHNGAISEVVLSFSVTFLVLLIILRGPRKLLAKTFLLALATVSVFVVGSKFTRPFMNPAIAFGWAYIYKSHNTWDHFYVYWISSYTGAILSAMLFRIIFPAPPLVQKKQKKA

>AtSIP2-1

MGRIGLVVTDLVLSFMWIWAGVLVNILVHGVLGFSRTDPSGEIVRYLFSIISMFIFAYLQQATKGGLYNPLTALAAGVSGGFSSFIFSVFVRIPVEVIGSILAVKHIIHVFPEIGKGPKLNVAIHHGALTEGILTFFIVLLSMGLTRKIPGSFFMKTWIGSLAKLTLHILGSDLTGGCMNPAAVMGWAYARGEHITKEHLLVYWLGPVKATLLAVWFFKVVFKPLTEEQEKPKAKSEFCNVFFFYVANSTVSTSSIKSEI

>AtTIP1-1

MPIRNIAIGRPDEATRPDALKAALAEFISTLIFVVAGSGSGMAFNKLTENGATTPSGLVAAAVAHAFGLFVAVSVGANISGGHVNPAVTFGAFIGGNITLLRGILYWIAQLLGSVVACLILKFATGGLAVPAFGLSAGVGVLNAFVFEIVMTFGLVYTVYATAIDPKNGSLGTIAPIAIGFIVGANILAGGAFSGASMNPAVAFGPAVVSWTWTNHWVYWAGPLVGGGIAGLIYEVFFINTTHEQLPTTDY

>AtTIP1-2

MPTRNIAIGGVQEEVYHPNALRAALAEFISTLIFVFAGSGSGIAFNKITDNGATTPSGLVAAALAHAFGLFVAVSVGANISGGHVNPAVTFGVLLGGNITLLRGILYWIAQLLGSVAACFLLSFATGGEPIPAFGLSAGVGSLNALVFEIVMTFGLVYTVYATAVDPKNGSLGTIAPIAIGFIVGANILAGGAFSGASMNPAVAFGPAVVSWTWTNHWVYWAGPLIGGGLAGIIYDFVFIDENAHEQLPTTDY

>AtTIP1-3

MPINRIAIGTPGEASRPDAIRAAFAEFFSMVIFVFAGQGSGMAYGKLTGDGPATPAGLVAASLSHAFALFVAVSVGANVSGGHVNPAVTFGAFIGGNITLLRAILYWIAQLLGAVVACLLLKVSTGGMETAAFSLSYGVTPWNAVVFEIVMTFGLVYTVYATAVDPKKGDIGIIAPLAIGLIVGANILVGGAFDGASMNPAVSFGPAVVSWIWTNHWVYWVGPFIGAAIAAIVYDTIFIGSNGHEPLPSNDF

>AtTIP2-1

MAGVAFGSFDDSFSLASLRAYLAEFISTLLFVFAGVGSAIAYAKLTSDAALDTPGLVAIAVCHGFALFVAVAIGANISGGHVNPAVTFGLAVGGQITVITGVFYWIAQLLGSTAACFLLKYVTGGLAVPTHSVAAGLGSIEGVVMEIIITFALVYTVYATAADPKKGSLGTIAPLAIGLIVGANILAAGPFSGGSMNPARSFGPAVAAGDFSGHWVYWVGPLIGGGLAGLIYGNVFMGSSEHVPLASADF

>AtTIP2-2

MVKIEIGSVGDSFSVASLKAYLSEFIATLLFVFAGVGSALAFAKLTSDAALDPAGLVAVAVAHAFALFVGVSIAANISGGHLNPAVTLGLAVGGNITVITGFFYWIAQCLGSIVACLLLVFVTNGESVPTHGVAAGLGAIEGVVMEIVVTFALVYTVYATAADPKKGSLGTIAPIAIGFIVGANILAAGPFSGGSMNPARSFGPAVVSGDFSQIWIYWVGPLVGGALAGLIYGDVFIGSYAPAPTTESYP

>AtTIP2-3

MVKIEVGSVGDSFSVSSLKAYLSEFIATLLFVFAGVGSAVAFAKLTSDGALDPAGLVAIAIAHAFALFVGVSIAANISGGHLNPAVTLGLAIGGNITLITGFFYWIAQCLGSIVACLLLVFVTNGKSVPTHGVSAGLGAVEGVVMEIVVTFALVYTVYATAADPKKGSLGTIAPIAIGFIVGANILAAGPFSGGSMNPARSFGPAVVSGDLSQIWIYWVGPLVGGALAGLIYGDVFIGSYEAVETREIRV

>AtTIP3-1

MATSARRAYGFGRADEATHPDSIRATLAEFLSTFVFVFAAEGSILSLDKLYWEHAAHAGTNTPGGLILVALAHAFALFAAVSAAINVSGGHVNPAVTFGALVGGRVTAIRAIYYWIAQLLGAILACLLLRLTTNGMRPVGFRLASGVGAVNGLVLEIILTFGLVYVVYSTLIDPKRGSLGIIAPLAIGLIVGANILVGGPFSGASMNPARAFGPALVGWRWHDHWIYWVGPFIGSALAALIYEYMVIPTEPPTHHAHGVHQPLAPEDY

>AtTIP3-2

MATSARRAYGFGRADEATHPDSIRATLAEFLSTFVFVFAGEGSILALDKLYWDTAAHTGTNTPGGLVLVALAHALALFAAVSAAINVSGGHVNPAVTFAALIGGRISVIRAIYYWVAQLIGAILACLLLRLATNGLRPVGFHVASGVSELHGLLMEIILTFALVYVVYSTAIDPKRGSIGIIAPLAIGLIVGANILVGGPFDGASMNPARAFGPALVGWRWSNHWIYWVGPFIGGALAALIYEYMIIPSVNEPPHHSTHQPLAPEDY

>AtTIP4-1

MKKIELGHHSEAAKPDCIKALIVEFITTFLFVFAGVGSAMATDSLVGNTLVGLFAVAVAHAFVVAVMISAGHISGGHLNPAVTLGLLLGGHISVFRAFLYWIDQLLASSAACFLLSYLTGGMGTPVHTLASGVSYTQGIIWEIILTFSLLFTVYATIVDPKKGSLDGFGPLLTGFVVGANILAGGAFSGASMNPARSFGPALVSGNWTDHWVYWVGPLIGGGLAGFIYENVLIDRPHVPVADDEQPLLN

>AtTIP5-1

MRRMIPTSFSSKFQGVLSMNALRCYVSEFISTFFFVLAAVGSVMSSRKLMAGDVSGPFGVLIPAIANALALSSSVYISWNVSGGHVNPAVTFAMAVAGRISVPTAMFYWTSQMIASVMACLVLKVTVMEQHVPIYKIAGEMTGFGASVLEGVLAFVLVYTVFTASDPRRGLPLAVGPIFIGFVAGANVLAAGPFSGGSMNPACAFGSAMVYGSFKNQAVYWVGPLLGGATAALVYDNVVVPVEDDRGSSTGDAIGV
